# Barriers, facilitators, and other factors associated with health behaviors in childhood, adolescent, and young adult cancer survivors: A systematic review

**DOI:** 10.1002/cam4.7361

**Published:** 2024-06-21

**Authors:** Ismay A. E. de Beijer, Eline Bouwman, Renée L. Mulder, Philippa Steensma, Morven C. Brown, Vera Araújo‐Soares, Magdalena Balcerek, Edit Bardi, Jeanette Falck Winther, Line Elmerdahl Frederiksen, Marloes van Gorp, Sara Oberti, Rebecca J. van Kalsbeek, Tomas Kepak, Katerina Kepakova, Hannah Gsell, Anita Kienesberger, Raphaële van Litsenburg, Luzius Mader, Gisela Michel, Monica Muraca, Selina R. van den Oever, Helena J. H. van der Pal, Katharina Roser, Roderick Skinner, Iridi Stolman, Anne Uyttebroeck, Leontien C. M. Kremer, Jacqueline Loonen, Elvira C. van Dalen, Saskia M. F. Pluijm, the PanCareFollowUp Consortium, Leontien C. M. Kremer, Helena J. H. van der Pal, Renée L. Mulder, Saskia M. F. Pluijm, Rebecca J. van Kalsbeek, Selina R. van den Oever, Lars Hjorth, Cecilia Follin, Lill Eriksson, Thomas Relander, Jacob Engellau, Karin Fjordén, Karolina Bogefors, Anna S. Holmqvist, Riccardo Haupt, Monica Muraca, Brigitte Nicolas, Francesca Bagnasco, Marina Benvenuto, Anna Aulicino, Luca Laudisi, Vera Araújo‐Soares, Tomas Kepak, Katerina Kepakova, Hana Hrstkova, Viera Bajciova, Marta Holikova, Lucie Strublova, Anne Uyttebroeck, Marleen Renard, Sandra Jacobs, Heidi Segers, Monique van Helvoirt, Jeanette F. Winther, Luzius Mader, Line E. Frederiksen, Elisabeth A. W. Andersen, Gisela Michel, Stefan Boes, Katharina Roser, Jacqueline Loonen, Rosella Hermens, Irene Göttgens, Eline Bouwman, Iridi Stollman, Adriaan Penson, Dionne Breij, Roderick Skinner, Morven C. Brown, Samira Essiaf, Anne Blondeel, William Sciberras, Joke Korevaar, Mieke Rijken, Anita Kienesberger, Jaap den Hartogh, Hannah Gsell, Carina Schneider, Edit Bardi, Jeroen te Dorsthorst

**Affiliations:** ^1^ Princess Máxima Center for Pediatric Oncology Utrecht The Netherlands; ^2^ Department of Hematology Radboud University Medical Center, Radboud Institute for Health Sciences Nijmegen The Netherlands; ^3^ Population Health Sciences Institute, Centre for Cancer Newcastle University Newcastle upon Tyne UK; ^4^ Center for Preventive Medicine and Digital Health, Department for Prevention, Medical Faculty Mannheim Heidelberg University Mannheim Germany; ^5^ Department of Pediatric Oncology and Hematology Charité – Universitätsmedizin Berlin, corporate member of Freie Universität Berlin and Humboldt Universität zu Berlin Berlin Germany; ^6^ St Anna Children's Hospital Vienna Austria; ^7^ Department of Pediatric and Adolescent Medicine Kepler University Clinic Linz Austria; ^8^ Childhood Cancer Research Group, Danish Cancer Society Research Center Copenhagen Denmark; ^9^ Department of Clinical Medicine and Faculty of Health Aarhus Universitet Aarhus Denmark; ^10^ DOPO clinic, Department of Hematology/Oncology IRCCS Istituto Giannina Gaslini Genoa Italy; ^11^ International Clinical Research Center St. Anne's University Hospital Brno Brno Czech Republic; ^12^ Childhood Cancer International Europe Vienna Austria; ^13^ Institute of Social and Preventive Medicine, University of Bern Bern Switzerland; ^14^ Faculty of Health Sciences and Medicine University of Lucerne Lucerne Switzerland; ^15^ PanCare Bussum The Netherlands; ^16^ Great North Children's Hospital, Royal Victoria Infirmary Newcastle Upon Tyne UK; ^17^ Translational and Clinical Research Institute, Wolfson Childhood Cancer Research Centre Newcastle Upon Tyne UK; ^18^ Department of Oncology, Paediatric Oncology, KU Leuven, Department of Paediatric Haematology and Oncology University Hospitals Leuven Leuven Belgium; ^19^ Department of Paediatrics Emma Children's Hospital Amsterdam The Netherlands; ^20^ Faculty of Medicine Utrecht University and Utrecht Medical Center Utrecht The Netherlands

**Keywords:** barriers, CAYA cancer survivors, facilitators, factors, health behaviors, healthy lifestyle, pediatric oncology, systematic review

## Abstract

**Background:**

Healthy behaviors are paramount in preventing long‐term adverse health outcomes in childhood, adolescent, and young adult (CAYA) cancer survivors. We systematically reviewed and synthesized existing literature on barriers, facilitators, and other factors associated with health behaviors in this population.

**Methods:**

MEDLINE and PsycInfo were searched for qualitative and quantitative studies including survivors aged 16–50 years at study, a cancer diagnosis ≤25 years and ≥2 years post diagnosis. Health behaviors included physical activity, smoking, diet, alcohol consumption, sun exposure, and a combination of these behaviors (defined as health behaviors in general).

**Results:**

Barriers, facilitators, and other factors reported in ≥2 two studies were considered relevant. Out of 4529 studies, 27 were included (*n* = 31,905 participants). Physical activity was the most frequently examined behavior (*n* = 12 studies), followed by smoking (*n* = 7), diet (*n* = 7), alcohol (*n* = 4), sun exposure (*n* = 4), and health behavior in general (*n* = 4). Relevant barriers to physical activity were fatigue, lack of motivation, time constraints, and current smoking. Relevant facilitators were perceived health benefits and motivation. Influence of the social environment and poor mental health were associated with more smoking, while increased energy was associated with less smoking. No relevant barriers and facilitators were identified for diet, alcohol consumption, and sun exposure. Barriers to healthy behavior in general were unmet information needs and time constraints whereas lifestyle advice, information, and discussions with a healthcare professional facilitated healthy behavior in general. Concerning other factors, women were more likely to be physically inactive, but less likely to drink alcohol and more likely to comply with sun protection recommendations than men. Higher education was associated with more physical activity, and lower education with more smoking.

**Conclusion:**

This knowledge can be used as a starting point to develop health behavior interventions, inform lifestyle coaches, and increase awareness among healthcare providers regarding which survivors are most at risk of unhealthy behaviors.

## 1 INTRODUCTION

The number of childhood, adolescent, and young adult (CAYA) cancer survivors is increasing due to survival rates of up to approximately 80% in high‐income countries.^1^, ^2^ Unfortunately, 75% of long‐term CAYA cancer survivors experience adverse health outcomes later in life, such as cardiovascular and musculoskeletal disease, metabolic syndrome, cancer‐related fatigue, anxiety, and depression.^3^, ^4^, ^5^, ^6^, ^7^, ^8^ These health outcomes can lead to hospitalization, disability, reduced quality of life, and premature mortality.^9^

Unhealthy behaviors—including insufficient physical activity, smoking, an unbalanced diet, excessive alcohol consumption, and unprotected sun exposure—are associated with an increased risk of developing adverse health outcomes in CAYA cancer survivors.^10^, ^11^, ^12^, ^13^, ^14^, ^15^, ^16^ Health behavior change interventions are effective and feasible in reducing these risks.^17^, ^18^, ^19^, ^20^, ^21^ Consequently, the adoption and maintenance of healthy behaviors has become paramount in the prevention of long‐term adverse health outcomes in CAYA cancer survivors.

However, CAYA cancer survivors may face specific barriers and facilitators when trying to adopt healthy behaviors. For example, physical limitations resulting from cancer treatment, chronic pain, and fatigue may hinder engagement in physical activity.^22^, ^23^ On the contrary, survivors might be more aware of their elevated health risks, which may increase their motivation to change their behavior.^24^ Positive or negative attitudes and beliefs also play an important role in shaping survivors' willingness to adopt healthy behaviors.^25^, ^26^

In order to develop targeted interventions tailored to the individual needs and preferences of CAYA cancer survivors, more knowledge is needed about the barriers, facilitators, and other factors associated with their health behaviors. In addition, a comprehensive understanding of the barriers, and facilitators that influence the different health behaviors of CAYA cancer survivors can inform guidelines and help healthcare providers (HCPs) involved in survivorship care to promote healthy habits. Knowledge about other factors, such as sociodemographic, treatment‐ and clinical factors, can be used to raise awareness among CAYA cancer survivors at high risk of unhealthy lifestyle behaviors. Therefore, this systematic review aimed to synthesize the existing evidence on the relevant barriers, facilitators, and other factors associated with health behaviors in CAYA cancer survivors.

## 2 METHODS

### 2.1 Inclusion and exclusion criteria

#### 2.1.1 Study designs

All study designs were eligible for inclusion except narrative and systematic reviews and case reports. We only included quantitative studies that reported multivariable models, as these provide more robust analyses and better control for confounding variables, increasing the reliability of the results. In addition, only English‐language studies published after 2000 were included, as earlier research largely overlooked the impact of health behavior on late adverse health outcomes.

#### 2.1.2 Participants

To ensure inclusivity without compromising reliability and to account for differences in international definitions of age thresholds for childhood cancer, studies were eligible if ≥75% of the study population was diagnosed with cancer <25 years of age, ≥50% of the population was ≥2 years after their primary cancer diagnosis, and participants were aged 16–50 years at the time of study. Studies including participants still undergoing cancer treatment were excluded. Studies with mixed samples (e.g., survivors aged < and ≥25 years at diagnosis) were included if results allowed separation.

#### 2.1.3 Outcomes

Outcomes were barriers, facilitators, and other factors associated with physical activity, smoking, diet, alcohol consumption, sun exposure, and a combination of health behaviors (health behavior in general). Barriers were interpreted as influencing the persistence of unhealthy behaviors or hindering/limiting healthy behaviors, whereas facilitators were interpreted as factors supporting engagement in healthier behaviors. In addition, barriers and facilitators were interpreted as potentially modifiable, for example, through lifestyle interventions. Other variables that were associated with health behavior and behavior change but were considered non‐modifiable or very difficult to modify were categorized separately as other factors, that is, sociodemographic and clinical characteristics. Factors that influence the persistence of unhealthy behaviors or hinder/limit healthy behaviors were termed “risk factors”, while factors that support engagement in healthier behaviors were termed “supportive factors”.

We distinguished between outcomes derived from qualitative and/or (semi‐)quantitative survey studies and those derived from quantitative studies, including observational or (semi)experimental studies with measures of association as outcomes. All suboutcomes, such as—in the case of smoking—smoking cessation, smoking rate, and quit attempts, were aggregated to the primary health behavior of interest, that is, smoking.

### 2.2 Search strategy for identification of studies

We conducted a systematic literature search in MEDLINE (Ovid until 15 April 2021 and PubMed from 15 April 2021 to 26 April 2023 for the updated search) and PsycInfo until 15 April 2021 (Appendix B). Reference lists of included studies and reviews were searched for studies not included in the electronic database searches. All authors were asked to identify any missing studies that had not been identified in the previous searches.

### 2.3 Study selection

After removing duplicates, two independent reviewers assessed the titles and abstracts using Rayyan (https://rayyan.ai). Studies meeting the inclusion criteria were retrieved for full‐text review. The reviewers discussed discrepancies that arose at either stage. Third party arbitration was not required. Two studies by Emmons et al. partially overlapped: Emmons et al. (2003) described the baseline data collection and intervention design of the Partnership for Health Study, and Emmons et al. (2005) described the outcomes of the intervention.^20^, ^27^ As both studies met the inclusion criteria, we included them in our review.

### 2.4 Data extraction

Standardized evidence tables (Appendix S1) were created to ensure the accuracy and consistency of data collection. These evidence tables included information on study design, participant characteristics, and outcomes. The tables were prepared by one author and checked by another author. In case of discrepancies or disagreements, the authors agreed by discussion.

### 2.5 Data synthesis

We have summarized the results in two separate tables: one for barriers and facilitators from qualitative and (semi‐)quantitative studies (Table 1) and one for significant barriers, facilitators, and other factors from quantitative studies (Table 2). These tables contain information about the study design, the participants and a summary of findings. Additionally, we created two overview tables (Tables 3 and 4) for all the barriers, facilitators, and other factors extracted from Tables 1 and 2. We categorized the barriers, facilitators, and other factors outlined in Tables 3 and 4 based on their content to enhance clarity and readability. Furthermore, we presented tables 3 and 4 using a three‐level color scheme. Barriers/risk factors preventing changes from unhealthy to healthy behaviors are shown in red, while facilitators/supportive factors associated with healthier behavior are shown in green. Darker colors indicate higher frequencies of specifically identified barriers, facilitators, or other factors on a 3‐point scale. Barriers, facilitators, and other factors reported in at least two studies were considered relevant and are described in the results.

**TABLE 1:** Qualitative and semi‐quantitative survey studies examining barriers and facilitators to health behaviors.

Study (design) and participants (*N*)	Barriers and facilitators
Physical activity	
Keats et al. 2007 (elicitation survey, 59 adolescent cancer survivors)	*Adopting a physically active lifestyle* Barriers: Feeling lazy or unmotivated to be physically active Being too busy or not having enough time Experiencing physical limitations, for example, being unfit, lack of stamina or strength, poor balance, fear of injury, treatment‐related side effects Experiencing fatigue or soreness, lack of ability, skill or confidence Lack of money or access to resources, for example, fitness facilities Negative parental influence, i.e. overprotective or not encouraging physical activity Facilitators: Staying physically fit, strong, and look good Staying healthy Staying busy and stay connected with friends Feeling normal Weight management Increased energy Having fun, increasing self‐confidence, and feeling a sense of accomplishment Reducing stress, relieving frustrations and relaxing Recovering from treatment
Arroyave et al. 2008 (cross‐sectional single‐center survey study, 118 CCS)	*Increasing exercise* Barriers (as indicated by descriptive statistics): Feeling too tired Being too busy Not belonging to a gym
Le et al. 2017 (pilot intervention study, 19 CCS)	*Adopting a physically active lifestyle* Barriers (as indicated by descriptive statistics): Lack of time Lack of support or companionship from family and friends Lack of energy Lack of motivation Lack of knowledge Lack of access to exercise resources or facilities Fear of injury Facilitators (as indicated by descriptive statistics): Regular exercise helps with some of the long‐term side effects of cancer treatment Participating in more exercise can help maintaining survivor's health
Dugan et al. 2021 (qualitative concept elicitation survey study, 17 CCS)	*Physical activity* Barriers: Health problems (e.g., sickness, fatigue, pain) Too little time Low motivation Having a baby Sedentary profession Distance too long Finance problems Facilitators: Time (e.g., no homework deadlines) Self‐motivation Peer support Family support Proximity to classes, parks, gyms, etc. Having adaptive equipment Obligatory classes at school
Marchak et al. 2023 (Cross‐sectional survey study, 27 CCS)	*Physical activity* Barriers (as indicated by descriptive statistics): Fatigue Decreased strength Low motivation for exercise Exercise limitations due to physical changes Too much screen time Fears related to injury Weight gaining leading to trouble with being physically active Doctors continuing to limit physical activities
Diet	
Arroyave et al. 2008 (cross‐sectional single‐center survey study, 118 CCS)	*Eating more fruits and vegetables* Barriers (as indicated by descriptive statistics): Hard to get when dining out Not liking the taste Not available at home *Eating more whole grains* Barriers (as indicated by descriptive statistics): Hard to get when dining out Not liking the taste Family does not like them *Eating more high‐calcium foods* Barriers (as indicated by descriptive statistics): Hard to get when dining out Hurting stomach Not available at home *Limiting high‐fat foods* Barriers (as indicated by descriptive statistics): Commercials make high‐fat foods tempting Hard to get low‐fat foods when dining out Friends eat a lot of high‐fat foods
Alexander et al. 2022 (Cross‐sectional survey study, 27 young adult cancer survivors)	*Acquirement of healthy nutrition habits* Facilitators (as indicated by descriptive statistics): Support of a nutritionist Support of another survivor Self‐help Use of digital/print materials
Marchak et al. 2023 (cross‐sectional survey study, 27 CCS)	*Healthy nutrition* Barriers (as indicated by descriptive statistics): Picky eating Increased unhealthy foods or snacks Eating too little or getting full easily Relying on fast food or take out instead of cooking at home Limited willingness to eat fruits or vegetables Using unhealthy food as a reward Eating too much or hungry all the time Drinking sugary beverages
Health behavior in general	
Mayes et al. 2016 (semi‐structured interviews, 51 CAYA cancer survivors)	*Adopting a healthy lifestyle* Facilitators: Lifestyle advice and information provided on the internet, school, magazines and TV, friends and family, or spoken/written information from hospital staff Health promotion conversation initiated and provided by consultant pediatric/adolescent oncologist The possibility to contact LTFU care providers regarding lifestyle choices
Pugh et al. 2018 (individual interviews and focus groups, 13 CAYA cancer survivors)	*Health behavior change* (*including physical activity, diet, smoking, alcohol consumption, and sun safety*) Barriers: Resource unavailability Family influence (negative) Cancer‐related physical changes leading to less confidence and self‐efficacy toward being active Unmet lifestyle information needs Confusion/uncertainty from unclear or lack of advice Geographical barriers (distance) Financial barriers (traveling to support groups, paying for gyms) Time‐related barriers (preparing or cooking healthy meals) Facilitators: Self‐efficacy Confidence Knowledge and skills Family influence (positive) Peer support Health behavior information: specific CAYA cancer lifestyle related, non‐judgmental, accessible, attractive, age‐appropriate, concise, and preferably online or mobile application‐based
Bouwman et al. 2023 (focus groups and semi‐structured interviews, 32 CCS)	*Healthy lifestyle knowledge* Barriers: Healthcare professionals providing insufficient knowledge on importance of health behaviors in childhood cancer survivors Facilitators: Knowledge of importance health behaviors for childhood cancer survivor population Healthcare professionals providing knowledge on importance of health behaviors for childhood cancer survivors Healthcare professionals providing knowledge on how to engage in healthy behaviors Knowledge of family/friends/yourself on healthy behaviors *Consequences* Facilitators: Physical health benefits as consequences of healthy behaviors Long‐term health benefits as consequences of healthy behaviors *Environmental context and resources* Barrier: Lack of available time for healthy behaviors Facilitators: Available professional support to stimulate healthy behaviors Work environment stimulating healthy behaviors Social environment positively influencing healthy behaviors *Social influences* Barriers: Unhealthy behaviors by people in close environment Lack of social support in adopting healthy behaviors (Social) Media stimulating unhealthy behaviors Facilitators: Healthy behaviors of people in close environment Social support by people in close environment stimulating healthy behaviors (Social) Media stimulating healthy behaviors Dealing with negative influences from people in social environment *Beliefs about capabilities* Facilitators: Physical health benefits as consequences of healthy behaviors Long‐term health benefits as consequences of healthy behaviors *Reinforcement* Barrier: Lower motivation to engage in healthy behaviors by personal related aspects Facilitators: Positive reinforcement by personal‐related incentives Positive reinforcement by social/societal incentives Positive reinforcement by sport activity‐related incentives Positive reinforcement by distal rewarding of health behaviors Positive reinforcement by proximal rewarding of health behaviors *Memory, attention and decision processes* Facilitators: Healthy behaviors due to conscious decision‐making Healthy behaviors embedded in memory *Skills* Facilitator: Learning how to deal with physical limitations when wanting to engage in physical activity *Emotion* Barrier: Stress negatively affecting healthy behaviors *Behavioral regulation* Facilitator: Good planning to maintain healthy behaviors

Abbreviations: CAYA, childhood, adolescent, and young adult, CCS, childhood cancer survivors, LTFU, long‐term follow‐up.

*Note*: Barriers contribute to the persistence of unhealthy behaviors, while facilitators support the transition to healthier choices.

**TABLE 2:** Quantitative studies examining significant barriers, facilitators, and other factors associated with health behaviors.

Study (design) and participants (*N*)	Barriers, facilitators, and other factors significantly associated with health behaviors
Physical activity (PA)	
Florin et al. 2007 (cross‐sectional multi‐center survey study, 2648 CCS)	Not meeting physical activity recommendations Female sex (vs. male sex), OR 1.2, 95% CI (1.1–1.2) Ethnicity (vs. White Non‐Hispanic): being Black Non‐Hispanic, OR 1.5, 95% CI (1.5–1.6); Being Other Non‐Hispanic, OR 1.2, 95% CI (1.1–1.2); being Hispanic (vs. White Non‐Hispanic), OR 1.4, 95% CI (1.4–1.5) Lower income < $20,000 (vs. ≥ $20,000), OR 1.2, 95% CI (1.2–1.3) Education (vs. college graduate); some high school education, OR 1.5, 95% CI (1.4–1.6); graduated high school, OR 1.3 (1.2–1.3); some college or vocational school, OR 1.1, 95% CI (1.1–1.2) ALL treatment (vs. female control): chemo only, OR 1.3, 95% CI (1.1–1.6); chemo + CRT < 20 Gy, OR 1.4, 95% CI (1.2–1.8); chemo + CRT > 20 Gy, OR 2.1, 95% CI (1.7–2.6) ALL treatment (vs. male control): chemo + CRT < 20 Gy, OR 1.4, 95% CI (1.1–1.7); chemo + CRT > 20 Gy (vs. male control), OR 1.4, 95% CI (1.2–1.8) Inactive lifestyle Survivors (vs. non‐survivors), OR 1.7, 95% CI (1.6–1.9) Female sex (vs. male sex), OR 1.3, 95% CI (1.3–1.4) Ethnicity (vs. White Non‐Hispanic): being Black Non‐Hispanic, OR 1.7, 95% CI (1.6–1.8); being Other Non‐Hispanic, OR 1.3, 95% CI (1.2–1.4); being Hispanic (vs. White Non‐Hispanic), OR 1.9, 95% CI (1.8–2.0) Lower income < $20,000 (vs. income ≥ $20,000), OR 1.7, 95% CI (1.6–1.7) Education (vs. college graduate): some high school education, OR 3.8, 95% CI (3.6–4.0); graduated high school, OR 2.5, 95% CI (2.4–2.6); some college or vocational school (vs. college graduate), OR 1.5, 95% CI (1.5–1.6) Current smoker (vs. no current smoker), OR 1.4, 95% CI (1.3–1.4) Female ALL survivor – chemo + CRT > 20 Gy (vs. female control), OR 1.9, 95% CI (1.5–2.3) Male ALL survivor – chemo + CRT < 20 Gy (vs. male control), OR 1.7, 95% CI (1.3–2.2) Male ALL survivor – chemo + CRT > 20 Gy (vs. male control), OR 1.9, 95% CI (1.5–2.3)
Cox et al. 2009 (cross‐sectional survey study, 838 CCS)	Higher physical activity participation (as indicated by Structural Equation Modeling) In men: More education Greater fear regarding future health Higher baseline exercise frequency Familiarity of primary care physician with cancer‐related problems In women: Greater self‐reported stamina Less fatigue Higher baseline exercise frequency Higher motivation
Ness et al. 2009 (cross‐sectional multi‐center study, 9301 CCS)	Not meeting physical activity recommendations Female sex (vs. male), RR 1.2, 99% CI (1.1–1.3) Non‐Hispanic Black (vs. Non‐Hispanic White), RR 1.2, 99% CI (1.2–1.3) Hispanic (vs. Non‐Hispanic White), RR 1.1, 99% CI (1.0–1.2) Other race/ethnicity (vs. Non‐Hispanic White), RR 1.1, 99% CI (1.0–1.2) Older age: 30–49 years vs. 18–29 years, RR 1.1, 99% CI (1.0–1.2), ≥ 50 years vs. 18–29 years, RR 1.2, 99% CI (1.1–1.4) Higher education: high school graduate vs. < high school, RR 0.9, 99% CI (0.8–1.0) Being a student (vs. working/ caring for home/family), RR 0.8, 99% CI (0.7–0.9) Being unable to work (vs. working/ caring for home/family), RR 1.2, 99% CI (1.1–1.3) Being either underweight or obese (vs. normal weight), RR 1.2, 99% CI (1.1–1.3) Being overweight (vs. normal weight); RR 1.2, 99% CI (1.0–1.2) Ever smoking (vs. never), RR 0.9, 99% CI (0.8–1.0) Women: amputation of lower limb (vs. no surgery), RR 1.3, 99% CI (1.2–1.5) Men: amputation of lower limb (vs. no surgery), RR 1.3, 99% CI (1.1–1.5) Other surgery (vs. no surgery), RR 1.1, 99% CI (1.0–1.2) Women: chemotherapy including anthracyclines (vs. no chemotherapy), RR 1.1, 99% CI (1.0–1.2) Women: any cranial radiation (vs. no radiation), RR 1.2, 99% CI (1.1–1.3) Women: other radiation (vs. no radiation), RR 1.1, 99% CI (1.0–1.2) Men: chest radiation without cranial radiation (vs. no radiation), RR 1.1, 99% CI (1.0–1.2) Inactive lifestyle Female sex (vs. male), RR 1.2, 99% CI (1.1–1.3) Non‐Hispanic Black (vs. Non‐Hispanic White), RR 1.7, 99% CI (1.3–2.2) Older age: 30–39 years (vs. 18–29 years), RR 1.5, 95% CI (1.3–1.7); 40–49 years (vs. 18–29 years), RR 1.5, 95% CI (1.3–1.8); ≥50 years (vs. 18–29 years), RR 2.0, 99% CI (1.4–3.0) Higher education: high school graduate vs. < high school, RR 0.8, 99% CI (0.6–1.0); college graduate vs. < high school, RR 0.4, 99% CI (0.3–0.6) Being unemployed, looking for work (vs. caring for home or family), RR 1.3, 99% CI (1.0–1.6) Being unable to work (vs. caring for home or family), RR 2.1, 99% CI (1.7–2.5) Being either underweight (vs. normal weight), RR 1.5, 99% CI (1.2–1.9) Being obese (vs. normal weight), RR 1.4, 99% CI (1.3–1.7) Being a current smoker (vs. never smoker), RR 1.5, 99% CI (1.2–1.9) Being depressed at time of interview (vs. not depressed), RR 1.4, 99% CI (1.2–1.7) Women: amputation of lower limb (vs. no surgery), RR 1.6, 99% CI (1.2–1.5) Men: amputation of lower limb (vs. no surgery), RR 1.4, 99% CI (1.0–1.9) Women: other surgery (vs. no surgery), RR 1.2, 99% CI (1.0–1.4) Women: chemotherapy including and without anthracyclines (vs. no chemotherapy), RR 1.1, 99% CI (1.0–1.3) Women: any cranial radiation (vs. no radiation), RR 1.5, 99% CI (1.3–1.7) Men: any cranial radiation (vs. no radiation), RR 1.3, 99% CI (1.1–1.6)
Rueegg et al. 2012 (cross‐sectional multi‐center study, 1058 CCS)	Inactivity Being female, OR 1.7, 95% CI (1.2–2.2) Education: compulsory schooling vs. vocational training, OR = 1.9, 95% CI (1.1–3.3), upper secondary education vs. vocational training, OR 1.4, 95% CI (1.1–2.0), university education vs. vocational training, OR 1.8, 95% CI (1.0–3.3) Underweight vs. normal weight, OR 3.0, 95% CI (1.3–6.8) Obese vs. normal weight, OR 1.5, 95% CI (0.8–2.6) No sports Education: compulsory schooling vs. vocational training, OR = 1.7, 95% CI (1.0–2.9), upper secondary education vs. vocational training, OR 0.9, 95% CI (0.6–1.2), university education vs. vocational training, OR 0.5, 95% CI (0.3–1.0) Having children, OR 1.8, 95% CI (1.0–3.2) Underweight vs. normal weight, OR 1.2, 95% CI (0.6–2.6) Obese vs. normal weight, OR 2.3, 95% CI (1.3–4.1) Current smoking, OR 1.9, 95% CI (1.4–2.6)
Rueegg et al. 2012 (cross‐sectional multi‐center study, 1038 CCS)	Any limitations in sports Parent's education (vs. secondary education): primary education, OR 0.4, 95% CI (0.1–1.2); unknown education, OR 3.0, 95% CI (1.2–7.4) Cancer diagnosis (vs. leukemia): lymphoma, OR 1.2, 95% CI (0.5–2.7); CNS tumor, OR 9.4, 95% CI (4.3–20.7); neuroblastoma, OR 3.8, 95% CI (1.2–11.6); retinoblastoma, OR 8.6, 95% CI (2.3–32.3); renal & hepatic tumors, OR 1.7, 95% CI (0.6–4.8); bone tumor, OR 13.6, 95% CI (5.6–33.3); soft tissue sarcoma, OR 2.9, 95% CI (1.1–7.7); germ cell tumor, OR 2.1, 95% CI (0.4–10.1); other tumors, OR 5.7, 95% CI (1.0–31.8); Langerhans cell histiocytosis, OR 1.6, 95% CI (0.4–7.5) Cancer treatment (vs. chemotherapy): surgery only, OR 0.4, 95% CI (0.1–0.9); radiotherapy, OR 1.6, 95% CI (0.9–2.9); bone marrow transplantation, OR 0.9, 95% CI (0.2–3.3) Any limitations in daily activities Parent's education (vs. secondary education): primary education, OR 1.9, 95% CI (1.0–3.5); tertiary education, OR 1.0, 95% CI (0.6–1.8), unknown education, OR 2.8, 95% CI (1.5–5.4) Cancer diagnosis (vs. leukemia): lymphoma, OR 0.9, 95% CI (0.5–1.8); CNS tumor, OR 5.8, 95% CI (3.1–10.8); neuroblastoma, OR 2.4, 95% CI (1.0–6.2); retinoblastoma, OR 2.9, 95% CI (0.8–10.1); renal & hepatic tumors, OR 2.2, 95% CI (1.0–4.6); bone tumor, OR 10.9, 95% CI (5.0–23.5); soft tissue sarcoma, OR 1.8, 95% CI (0.8–4.0); germ cell tumor, OR 1.2, 95% CI (0.3–4.2); other tumors, OR 1.9, 95% CI (0.3–10.0); Langerhans cell histiocytosis, OR 2.9, 95% CI (1.2–7.4) Cancer treatment (vs. chemotherapy): surgery only, OR 0.5, 95% CI (0.2–1.1); radiotherapy, OR 2.1, 95% CI (1.3–3.3); bone marrow transplantation, OR 3.0, 95% CI (1.2–7.1)
Slater et al. 2016 (cross‐sectional survey study, 158 CCS)	Engaging in active transportation Being married or living with a partner (vs. not), OR 0.3, 95% CI (0.1–0.8) Less planning/psychosocial barriers (vs. more), OR 0.2, 95% CI (0.0–0.5) Higher perceived walkability of the neighborhood (vs. lower), OR 2.6, 95% CI (1.1–5.7)
Darabos et al. 2021 (cross‐sectional survey study, 307 CCS)	Not meeting physical activity recommendations Non‐Hispanic race, OR 0.3, 95% CI (0.1–0.7) Longer time since treatment completion, OR 0.8, 95% CI (0.6–0.9)
Smoking	
Emmons et al. 2003 (randomized trial of a smoking cessation intervention, 796 smoking CCS)	Higher smoking rates (β represents the increase in the odds of higher smoking rates) Older current age: β = 0.0226, p < 0.0001 Education: less than high school vs. more than high school: β = 0.3311, p = 0.0019 Social norms: most smoke vs. none/few smoke: β = 0.5657 p < 0.0001; about half smoke vs. none/few smoke: β = 0.1881 p = 0.016 Support for quitting: a little or a lot vs. not at all: β = 0.2027, p = 0.0256 Higher score on the global severity index (severe psychological symptoms): β = 0.0077, p = 0.0238 Nicotine dependence Older age: OR 1.0, 95% CI (1.0–1.1) Low level of education: < high school vs. college graduate, OR 2.8, 95% CI (1.6–5.0); high school graduate vs. college graduate, OR 2.4, 95% CI (2.4–1.5); post high school vs. college graduate, OR 1.9, 95% CI (1.2–3.0) A greater proportion of smokers in social network: most smoke vs. non/few smoke, OR 2.2, 95% CI (1.5–3.2); about half smoke vs. non/few smoke, OR 1.7, 95% CI (1.2–2.4) Higher score on the global severity index (severe psychological symptoms): OR 1.7, 95% CI (1.1–2.7) More quit attempts Younger age, OR 1.0, 95% CI (1.0–1.0) Support for quitting: a little or a lot vs. not at all: OR 1.7, 95% CI (1.1–2.5) Social support: a lot vs. not at all, OR 1.7, 95% CI (1.1–2.5) Seeing oneself as more vulnerable to smoking‐related illnesses, OR 1.2, 95% CI (1.1–1.3) Social networks of which most smoke vs. none/few/half smoke, OR 0.6, 95% CI (0.5–0.9) Readiness to quit Support for quitting: a lot vs. not at all, OR 3.8, 95% CI (2.5–5.6), a little vs. not at all, OR 2.2, 95% CI (1.5–3.3) Seeing oneself as more vulnerable to smoking‐related illnesses, OR 1.2, 95% CI (1.1–1.3)
Emmons et al. 2005 (randomized trial of a smoking cessation intervention, 796 smoking CCS; overlap with Emmons et al. 2003)	Smoking cessation Counseling vs. self‐help at both the 8‐month (16.8% vs. 8.5%, *p* = 0.01) and 12‐month follow‐ups (15% vs. 9%, *p* = 0.01). Long‐term self‐efficacy (vs. little or no self‐efficacy), OR 1.4 (1.2–1.6) Having a lot of energy in the past 4 weeks, OR 1.4, 95% CI (1.1–2.0)
Kahalley et al. 2012 (cross‐sectional multi‐center survey, 307 CCS)	Smoking No history of cranial radiotherapy (vs. history of cranial radiotherapy), RR, 95% CI 2.4 (1.1–5.2) Household smoking (vs. no household smoking), RR 2.2, 95% CI (1.2–4.2) Suicidal behavior (vs. no suicidal behavior), RR 1.9, 95% CI 1.9 (1.0–3.6) Peer smoking and binge eating (vs. no peer smoking and no binge eating), RR 3.4, 95% CI (1.2–9.7)
Bougas et al. 2021 (cohort study, 2887 CCS)	Smoking Being a CNS tumor survivor (vs. Wilms tumor survivors), RR 0.4, 95% CI (0.3–0.6). Treatment with chemotherapy RR 0.9, 95% CI (0.7–1.0) Treatment with thoracic radiation therapy RR 0.8, 95% CI (0.6–1.0) Having had a second cancer, RR 0.7, 95% CI (0.4–1.0) Having (had) a cardiovascular disease, RR 0.7, 95% CI (0.5–1.0) Being male, RR 1.4, 95% CI (1.2–1.6) Being married, RR 0.8, 95% CI (0.7–1.0) Being ≥40 years (vs. <30 years), RR 0.8, 95% CI (0.6–1.0) Higher education level: graduated from college (vs. high school dropout), RR 0.6, 95% CI (0.5–0.7) Poor physical quality of life score (< first quartile vs. others), RR 0.8, 95% CI (0.7–1.0) Poor mental quality of life score (<first quartile vs. > third quartile), RR 1.6, 95% CI (1.3–1.9) Quitting smoking Being male, RR 0.8, 95% CI (0.7–0.9) Being married, RR 1.2, 95% CI (1.1–1.4) Higher educational level, RR 1.5, 95% CI (1.2–1.7) Having had a second cancer, RR 1.3, 95% CI (1.0–1.6)
Cappelli et al. 2021 (cohort study, 127 young adult cancer survivors)	Smoking Higher cancer treatment intensity score, OR 0.3, 95% CI (0.1–0.8) Being a past smoker, OR 5.9, 95% CI (1.2–29.9)
Darabos et al. 2021 (cross‐sectional survey study, 307 CCS)	Smoking Female sex, OR 0.2, 95% CI (0.1–0.8) White race (vs. African American, Asian, American Indian/Alaskan native, other race), OR 11.4, 95% CI (1.2–104.8)
Cheung et al. 2022 (cross‐sectional survey study, 200 CCS)	Smoking Lower education level (vs. higher level than secondary school), OR 5.1, 95% CI (1.5–17.8)
Alcohol consumption	
Lown et al. 2008 (cross‐sectional survey study, 10,398 CCS)	Heavy drinking Younger age, OR 2.7, 95% CI (1.9–3.9) Being male, OR 2.1, 95% CI (1.8–2.6) Education: grades 0–12 vs. college graduate, OR 3.4, 95% CI (2.7–4.4); some post‐high school vs. college graduate, OR 2.2, 95% CI (1.7–2.8) Age of first drink: <14 years vs. 21+ years, OR 6.9, 95% CI (4.4–10.8), 15–16 years vs. 21+ years, OR 5.3, 95% CI (3.5–8.1), 17–20 years vs. 21+ years, OR 2.9, 95% CI (1.9–4.4) Older age at diagnosis: 15–21 years vs. 5–9 years, OR 0.7, 95% CI (0.5–1.0) Fair/poor general health (vs. excellent/very good/good), OR 1.5, 95% CI (1.1–1.9) Abnormal depression (vs. normal). OR 1.7, 95% CI (1.4–2.2) Abnormal anxiety (vs. normal), OR 1.4, 95% CI (1.1–1.9) Abnormal somatization (vs. normal), OR 1.7, 95% CI (1.3–2.2) Abnormal Global Severity Index score including depression, somatization and anxiety (vs. normal), OR 1.9, 95% CI (1.5–2.4) Activity limitations (vs. not limited at all), OR 1.3, 95% CI (1.1–1.5) Some anxiety about cancer (vs. none), OR 1.2, 95% CI (1.0–1.4) Cancer diagnosis (vs. leukemia): Hodgkin's disease OR 1.4, 95% CI (1.0–1.8), Wilms tumor OR 1.5 (1.1–2.1), neuroblastoma OR 1.6, 95% CI (1.1–2.3), and Bone tumor OR 1.7, 95% CI (1.2–2.2) Intrathecal methotrexate or cranial radiation, OR 0.7, 95% CI (0.5–0.8)
Cappelli et al. 2021 (cohort study, 127 young adult cancer survivors)	Binge drinking Female sex, OR 0.4, 95% CI = (0.2–1.0)
Darabos et al. 2021 (cross‐sectional survey study, 307 CCS)	Binge drinking Higher age at baseline, OR 1.9, 95% CI (1.1–3.4) Solid tumor diagnosis (vs. leukemia/lymphoma/brain tumor), OR 1.9, 95% CI (1.0–3.6) Higher intensity of treatment, OR 0.6, 95% CI (0.4–1.0)
Cheung et al. 2022 (cross‐sectional survey study, 200 CCS)	Alcohol consumption Female sex, OR 0.3, 95% CI (0.2–0.7) No private medical insurance, OR 0.4, 95% CI (0.2–0.9)
Diet	
Zhang et al. 2016 (retrospective cohort study with cross‐sectional assessment, 2570 CCS)	High diet quality based on adjusted means Healthy Eating Index–2010 (maximum score = 100): Higher age: 58.0 (56.7, 59.3) for age 40–64 years, 56.3 (55.2, 57.4) for age 30–39 years, 55.1 (54.0, 56.2) for age 18–29 years Female sex, 59.3 (58.3, 60.4), vs. male sex 53.6 (52.6, 54.7) Higher education level: college graduate 60.1 (59.4, 61.7) vs. some post high‐school 55.7 (54.6, 56.8) and grades 0–12 53.2 (52.0, 54.4 Non‐smokers 57.9 (57.0, 58.9) and former smokers 57.7 (56.1, 59.2) vs. current smokers 53.9 (52.7, 55.1) Being physically active 58.5 (57.5, 59.6) vs. inactive 54.5 (53.4, 55.5) Overweight 58.0 (57.0, 59.0) and normal weight 57.3 (56.3, 58.3) vs. underweight 54.1 (51.6, 56.7) and obesity 56.5 (55.5, 57.5) Primary cancer diagnosis with leukemia 58.7 (57.9, 59.5) and lymphoma 59.4 (58.3, 60.4) vs. embryonal tumors 56.9 (55.5, 58.2, sarcoma 57.3 (56.0, 58.6), CNS tumors 57.7 (56.1, 59.3), and other tumors 57.0 (55.2, 58.8) Age at diagnosis: 5–9 years (58.2 (57.1, 59.2), 10–14 years 58.5 (57.4, 59.5), and >15 years 58.2 (56.9, 59.5) vs. < 5 years 56.9 (56.0, 57.8) Lower abdomen radiation dose: 0 Gy 58.9 (58.0, 59.7) vs. 1–19.9 Gy 57.2 (55.0, 59.4), 20–29.9 Gy 56.7 (54.8, 58.5) and ≥ 30 Gy 56.1 (54.2, 58.0) 1500–8999 Mg/m2 cumulative glucocorticoid dose: 59.7 (57.9, 61.5) vs. 0 57.7 (56.9, 58.4), 1–1499 Mg/m2 57.1 (55.4, 58.7) and ≥ 9000 Mg/m2 56.9 (55.0, 58.5)
Bhandari et al. 2021 (cross‐sectional survey study, 446 CCS)	Vitamin D deficiency Hispanic or Black race (vs. non‐Hispanic white), OR 2.4, 95% CI (1.4–4.1) Being overweight (vs. normal/underweight), OR 1.8, 95% CI (1.0–3.1) Being obese (vs. normal/underweight), OR 2.4, 95% CI (1.4–4.1)
Cheung et al. 2022 (cross‐sectional survey study, 200 CCS)	Adoption of a balanced diet more than ≥4 days per week Younger age at interview, OR 1.0, 95% CI (0.9–1.0) Primary cancer diagnosis including hematological malignancies vs. CNS tumors, OR 2.5, 95% CI (1.3–4.7)
Darabos et al. 2021 (cross‐sectional survey study, 307 CCS)	Not meeting fruit/vegetable intake recommendations Having had a relapse, OR 0.5, 95% CI (0.2–1.0)
Sun exposure	
Zwemer et al. 2012 (cross‐sectional survey study, 153 young adult cancer survivors)	Low adherence to sunbathing recommendations Age 26–60 years (vs. <26 years), OR 0.4, 95% CI (0.2–0.9) Female sex, OR 2.4, 95% CI (1.1–5.5) Low adherence recommendations during incidental sun exposure Perceived vulnerability to appearance changes from UV exposure, OR 0.6, 95% CI (0.4–0.9)
Darabos et al. 2021 (cross‐sectional survey study, 307 CCS)	Engaging in unsafe sun protective habits Female sex, OR 0.6, 95% CI (0.3–1.0) Non‐Hispanic White race (vs. Hispanic), OR 0.4, 95% CI (0.2–0.8)
Cheung et al. 2022 (cross‐sectional survey study, 200 CCS)	Sunscreen use more than ≥4 days per week Female sex, OR 5.7, 95% CI (2.4–13.3) Educational level: secondary school or below vs. above secondary school, OR 0.2, 95% CI (0.1–0.8) Monthly household income: ≤$30,000 vs. >$30,000, OR 0.4, 95% CI (0.2–0.8)
Fluehr et al. 2023 (cross‐sectional survey study, 94 CAYA cancer survivors)	Increased sun protection behaviors (as indicated hierarchical linear regression) Being of fair/easily burned skin type (*p* = 0.02) Perceiving greater relative susceptibility to skin cancer compared with non‐cancer survivors (*p* = 0.02)
Health behavior in general	
Klosky et al. 2012 (retrospective multi‐center survey study, 307 CAYA cancer survivors)	Poor overall behavioral health Better mental health, OR 0.2, 95% CI (0.13–0.43)

Abbreviations: ALL, acute lymphocytic leukemia, BMI, body mass index, CAYA, childhood, adolescent, and young adult, CCS, childhood cancer survivors, CI, confidence interval, CNS, central nervous system, CRT, cranial radiotherapy, Gy, gray, RR, risk ratio, OR, odds ratio.

*Note*: This table displays only the significant study results; non‐significant results and descriptions of the models used for each included study are shown in the evidence tables (Supplementary File A). Barriers contribute to the persistence of unhealthy behaviors, while facilitators support the transition to healthier choies.

**TABLE 3:** Barriers and facilitators to health behaviors.

	Health behaviors											
	Health behavior in general		Smoking		Physical activity		Diet		Alcohol consumption		Sun exposure	
	Barriers	Facilitators	Barriers	Facilitators	Barriers	Facilitators	Barriers	Facilitators	Barriers	Facilitators	Barriers	Facilitators
Information, knowledge, and skills												
Lifestyle advice and information		*N* = 3 [28, 29, 30]										
Health promotion conversation with healthcare professional		*N* = 2 [28, 30]										
Being able to contact healthcare professional for lifestyle choices		*N* = 1 [28]										
Unmet information needs	*N* = 2 [29, 31]											
Lack of knowledge and skills					*N* = 2 [26, 32]							
Knowledge and skills		*N* = 1 [29]										
Learning how to deal with physical limitations		*N* = 1 [31]										
Counseling				*N* = 1 [20]								
Self‐management and cognitive processes												
Self‐help								*N* = 1 [33]				
Lack of self‐efficacy					*N* = 1 [26]							
Self‐efficacy		*N* = 1 [29]		*N* = 1 [20]								
Lack of confidence					*N* = 1 [26]							
Confidence		*N* = 1 [29]				*N* = 1 [26]						
Lack of motivation	*N* = 1 [30]				*N* = 4 [25, 26, 31, 34]							
(Self)‐motivation						*N* = 2 [31, 35]						
Positive reinforcement		*N* = 1 [30]										
Conscious decision‐making		*N* = 1 [30]										
Decisions embedded in memory		*N* = 1 [30]										
Health and wellbeing												
Cancer‐related physical changes	*N* = 1 [29]											
Perceived health benefits		*N* = 1 [31]				*N* = 2 [25, 26]						
Weight management						*N* = 1 [26]						
Fear of injury					*N* = 2 [25, 34]							
Foods hurting stomach							*N* = 1 [36]					
Perceived greater relative susceptibility to skin cancer												*N* = 1 [36]
Fatigue					*N* = 5 [25, 26, 34, 35, 37]							
Stamina						*N* = 1 [35]						
Decreased strength					*N* = 1 [34]							
Being underweight					*N* = 2 [38, 39]							
Being overweight					*N* = 2 [34, 38]		*N* = 1 [40]	*N* = 1 [41]				
Being obese					*N* = 2 [38, 39]		*N* = 1 [40]					
Poor general health				*N* = 1 [42]	*N* = 1 [31]				*N* = 1 [43]			
Experiencing physical limitations					*N* = 2 [26, 34]							
Having (had) cardiovascular disease				*N* = 1 [42]								
Environmental influences												
Positive family influence		*N* = 1 [29]				*N* = 1 [31]						
Negative family influence	*N* = 1 [29]		*N* = 1 [44]		*N* = 1 [26]							
Positive social environment		*N* = 1 [31]		*N* = 1 [27]								
Negative influence of social environment	*N* = 1 [31]		*N* = 2 [27, 44]				*N* = 1 [36]					
Lack of peer support	*N* = 1 [31]				*N* = 1 [25]							
Peer support		*N* = 1 [29]		*N* = 1 [27]		*N* = 1 [31]		*N* = 1 [33]				
Positive influence of social media		*N* = 1 [31]										
Negative influence of social media	*N* = 1 [31]											
Connectedness						*N* = 1 [26]						
Stimulating work environment		*N* = 1 [30]										
Sedentary profession					*N* = 1 [31]							
Obligatory classes at school						*N* = 1 [31]						
Doctors limiting physical activities					*N* = 1 [34]							
Resources												
Resource unavailability	*N* = 1 [29]				*N* = 1 [36]		*N* = 1 [36]					
Distance	*N* = 1 [29]				*N* = 1 [31]							
Lack of finances	*N* = 1 [29]				*N* = 2 [26, 31]							
No private medical insurance										*N* = 1 [34]		
Having enough time						*N* = 1 [31]						
Time constraints	*N* = 2 [29, 30]				*N* = 4 [25, 26, 31, 37]							
Available professional support		*N* = 1 [33]						*N* = 1 [33]				
Walkability of the neighborhood						*N* = 1 [45]						
Proximity of facilities						*N* = 1 [31]						
Having adaptive equipment						*N* = 1 [31]						
Digital prints/materials								*N* = 1 [33]				
Mental health and quality of life												
Good mental health		*N* = 1 [32]										
Poor mental health			*N* = 2 [27, 42]									
Depression					*N* = 1 [38]				*N* = 1 [43]			
Anxiety									*N* = 1 [43]			
Suicidal behavior			*N* = 1 [44]									
Increased energy				*N* = 2 [20, 26]								
Poor physical quality of life				*N* = 1 [42]								
Poor mental quality of life			*N* = 1 [42]									
Stress	*N* = 1 [30]											
Perceived vulnerability			*N* = 1 [27]									*N* = 1 [46]
Fear regarding future health						*N* = 1 [35]						
Behavioral regulation												
Too much screen time					*N* = 1 [34]							
Currently smoking					*N* = 3 [38, 39, 47]							
Not currently smoking								*N* = 1 [41]				
Being a past smoker			*N* = 1 [48]			*N* = 1 [38]						
Drinking initiation at a young age									*N* = 1 [43]			
Higher (baseline) exercise frequency						*N* = 1 [35]		*N* = 1 [41]				
Planning		*N* = 1 [30]				*N* = 1 [45]						
Not liking the taste of certain foods							*N* = 1 [36]					

*Note*: Numbers in brackets refer to the reference number of an included study. Barriers (red colors) contribute to the persistence of unhealthy behaviors, while facilitators (green colors) support (the transition to) healthier behaviors. The darkness of the color corresponds to the frequency of the barriers or facilitators on a 3‐point scale, with darker colors representing higher frequencies.

**TABLE 4:** Sociodemographic, cancer and treatment related and other factors associated with health behavior.

	Health behaviors											
	Health behavior in general		Smoking		Physical activity		Diet		Alcohol consumption		Sun exposure	
	Risk	Supportive	Risk	Supportive	Risk	Supportive	Risk	Supportive	Risk	Supportive	Risk	Supportive
Sociodemographic factors												
Male sex			*N* = 1 [42]						*N* = 2 [43, 48]			
Female sex				*N* = 1 [49]	*N* = 3 [38, 39, 47]			*N* = 1 [41]		*N* = 1 [34]	*N* = 1 [46]	*N* = 2 [34, 49]
Older age			*N* = 1 [27]	*N* = 1 [42]	*N* = 1 [38]			*N* = 1 [41]	*N* = 1 [49]			
Younger age				*N* = 1 [27]				*N* = 1 [34]	*N* = 1 [43]		*N* = 1 [46]	
Being married or living with a partner				*N* = 1 [42]		*N* = 1 [45]						
Having children					N = 2 [31, 39]							
Lower level of education			*N* = 2 [27, 34]		*N* = 2 [39, 47]				*N* = 1 [43]		*N* = 1 [34]	
Higher level of education				*N* = 1 [42]		*N* = 3 [35, 38, 39]						
Lower level of parent's education						*N* = 1 [50]						
Unknown parent's education					*N* = 1 [50]							
Non‐Hispanic White ethnicity			*N* = 1 [49]			*N* = 1 [49]						*N* = 1 [49]
Hispanic, Black or Other Non‐Hispanic ethnicity					*N* = 2 [38, 47]							
Hispanic or Black ethnicity							*N* = 1 [40]					
Lower income					*N* = 1 [47]						*N* = 1 [34]	
Being unable to work					*N* = 1 [38]							
Being a student						*N* = 1 [38]						
Cancer‐ and treatment‐related factors												
Being a survivor of lymphoma						*N* = 1 [50]		*N* = 1 [41]				
Being a survivor of CNS tumor				*N* = 1 [42]								
Solid tumor diagnosis									*N* = 1 [49]			
Diagnosis including hematological malignancies								*N* = 1 [34]				
Other cancer diagnosis than leukemia					*N* = 1 [50]				*N* = 1 [43]			
Cancer diagnosis at a younger age								*N* = 1 [41]				
Cancer diagnosis during late adolescence										*N* = 1 [43]		
Higher cancer treatment intensity				*N* = 1 [48]						*N* = 1 [49]		
Surgery only						*N* = 1 [50]						
Having had surgery					*N* = 1 [38]							
Chemotherapy				*N* = 1 [42]								
Radiotherapy					*N* = 2 [38, 50]							
Cranial radiotherapy					*N* = 1 [38]					*N* = 1 [43]		
No cranial radiotherapy			*N* = 1 [44]									
Chemotherapy and/or cranial radiotherapy					*N* = 1 [47]							
Thoracic radiotherapy				*N* = 1 [42]								
Bone marrow transplantation (vs. chemotherapy)					*N* = 1 [50]	*N* = 1 [50]						
Lower abdomen radiation dose								*N* = 1 [41]				
4600–8999 Mg/m2 cumulative glucocorticoid dose								*N* = 1 [41]				
Intrathecal methotrexate or cranial radiation								*N* = 1 [43]				
Familiarity of primary care physician with cancer‐related problems						*N* = 1 [35]						
Having had a relapse								*N* = 1 [49]				
Having had a second cancer				*N* = 1 [42]								
Longer time since treatment completion						*N* = 1 [49]						
Other factors												
Amputation of lower limb					*N* = 1 [38]							
Fair/easily burned skin type												*N* = 1 [51]

*Note*: Numbers in brackets refer to the reference number of an included study. Green colors indicate a positive association with the health behavior. Red colors indicate a negative association. The darkness of the color corresponds to the frequency of the factors on a 3‐point scale, with darker colors representing higher frequencies.

## 3 RESULTS

**FIGURE 1:**
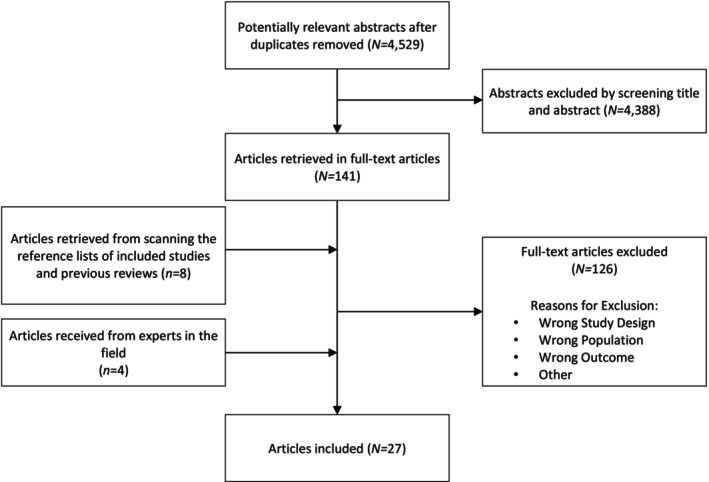
Flowchart of studies included and excluded from the systematic review.

After removing duplicates, 4529 abstracts were identified and screened. Next, 141 full‐text articles were assessed for inclusion in the review. Eight studies were identified by screening the reference lists of the included studies and relevant reviews, and four studies were identified by experts in the field. Finally, 27 studies met all eligibility criteria (Figure 1). Nine were qualitative or semiquantitative studies and 18 were quantitative studies. Study designs included interviews (*n* = 1), a combination of interviews and focus groups (*n* = 2), elicitation surveys (*n* = 2), a pilot intervention study (*n* = 1), randomized trials (*n* = 2), cohort studies (*n* = 3), cross‐sectional survey studies (*n* = 15), and a retrospective multi‐institution survey study (*N =* 1). Sample sizes ranged from 13 to 10,398, with a total of 31,905 participants across all included studies. All studies were conducted between 2003 and 2023. Five studies reported on more than one health behavior.^34^, ^37^, ^48^, ^49^, ^52^

### 3.1 Barriers, facilitators, and other factors associated with health behaviors (Tables 3 and 4)

#### 3.1.1 Physical activity (*n* = 12 studies, *n* = 15,588 participants)

Twenty‐one barriers, 17 facilitators, 17 risk factors, and 10 supportive factors associated with physical activity were reported in the included studies. Barriers reported in at least two studies were *fatigue* (*n* = 5^25^, ^26^, ^34^, ^35^, ^37^), lack of motivation (n = 4^25^, ^26^, ^31^, ^34^), time constraints (*n* = 4^25^, ^26^, ^31^, ^37^), being a current smoker (*n* = 3^38^, ^39^, ^47^), a lack of knowledge and skills (*n* = 2^25^, ^26^); fear of injury (*n* = 2^25^, ^34^); lack of finances (e.g., for a gym membership; *n* = 2^26^, ^31^), being underweight (*n* = 2^38^, ^39^), being overweight (*n* = 2^34^, ^38^), being obese (*n* = 2^38^, ^39^), and experiencing physical limitations (*n* = 2, e.g., poor balance or lack of fitness^26^, ^34^). Facilitators for physical activity reported in at least two studies were perceived health benefits (*n* = 2^25^, ^26^) and (self)‐motivation (*n* = 2^31^, ^35^).

Relevant risk factors for physical activity were female sex (*n* = 3^38^, ^47^), a treatment history including radiotherapy (*n* = 2^38^, ^50^), having children (*n* = 2^31^, ^39^), lower educational level (*n* = 2^39^, ^47^), and being of Hispanic, Black, or other non‐Hispanic ethnicity (*n* = 2^38^, ^47^). In contrast, higher levels of education (*n* = 3^35^, ^38^, ^39^) was a supportive factor for physical activity.

#### 3.1.2 Smoking (*n* = 7 studies, *n* = 5420 participants)

Seven barriers, six facilitators, five risk factors, and 12 supportive factors were associated with smoking in the included studies. Barriers reported in at least two studies were negative influence of the social environment (*n* = 2, e.g., smoking in the household or a higher proportion of smokers in the social network^27^, ^44^) and *poor mental health* (*n* = 2^27^, ^42^). On the contrary, increased energy (*n* = 2^20^, ^26^) was identified as a facilitator in at least two studies. In terms of factors, lower educational attainment (*n* = 2^27^, ^34^) was a risk factors for smoking.

#### 3.1.3 Diet (*n* = 7 studies, *n* = 3695 participants)

Six barriers (e.g., not liking the taste of certain foods), seven facilitators (e.g., peer support), two risk factors (e.g., Hispanic or Black ethnicity), and 10 supportive factors (e.g., female sex) were associated with diet in one of the included studies. No barriers, facilitators, or factors were reported in at least two studies.

#### 3.1.4 Alcohol consumption (*n* = 4 studies, *n* = 11,032 participants)

Three barriers, one facilitator, seven risk factors, and four supportive factors associated with (reducing) alcohol consumption were reported in the included studies. No barriers or facilitators were reported in at least two studies. However, in terms of factors, men had significantly higher levels of alcohol consumption than women, especially with regard to binge drinking (*n* = 2^43^, ^48^).

#### 3.1.5 Sun exposure (*n* = 4 studies, *n* = 754 participants)

The included studies reported zero barriers, two facilitators, four risk factors, and three supportive factors associated with (increased) sun exposure. No barriers or facilitators were reported in at least two studies. However, women were significantly more likely than men to adhere to sun exposure recommendations (*n* = 3^34^, ^46^, ^49^).

#### 3.1.6 Health behavior in general (*n* = 4 studies, *n* = 403 participants)

Twelve barriers and 19 facilitators associated with health behavior in general were identified in the included studies. Barriers identified in at least two studies were unmet information needs (*n* = 2^29^, ^30^) and *time constraints* (*n* = 2^29^, ^30^). Lifestyle advice and information (*n* = 3^28^, ^29^, ^30^) and having a health promotion conversation with a healthcare professional (*n* = 2^28^, ^30^) were identified as facilitators in at least two studies. There were no other factors associated with health behavior in general.

#### 3.1.7 Nonsignificant results

For the quantitative studies, Tables 1, 2, 3, 4 include only the significant results. Non‐significant results are reported in Appendix S2. Across all health behavior outcomes (NB: a single study may examine multiple outcomes), the most nonsignificant results were found for age at diagnosis (*n* = 17^28^, ^34^, ^39^, ^40^, ^46^, ^49^), cancer diagnosis (*n* = 14^28^, ^39^, ^43^, ^46^, ^49^), attained age (*n* = 14^20^, ^28^, ^34^, ^39^, ^40^, ^44^, ^45^, ^46^, ^49^, ^51^), cancer treatment (*n* = 12^28^, ^38^, ^39^, ^40^, ^41^, ^46^, ^49^, ^51^), sex (*n* = 11^20^, ^34^, ^39^, ^44^, ^48^, ^49^, ^51^), race/ethnicity (*n* = 9^28^, ^36^, ^41^, ^43^, ^46^, ^49^), and household income (*n* = 7^28^, ^34^, ^46^). None of the significant results identified in the included studies were outweighed by a greater number of nonsignificant results. In other words, the results described in Tables 1, 2, 3, 4 were all found to be statistically significant more often than statistically nonsignificant.

## 4 DISCUSSION

To our knowledge, this systematic review is the first to provide a comprehensive overview of the evidence on barriers, facilitators, and other factors associated with health behaviors in CAYA cancer survivors. Physical activity was the most commonly studied health behavior in this systematic review. The most frequently identified barriers to physical activity were fatigue, time constraints, lack of motivation, current smoking, lack of knowledge and skills, fear of injury, financial constraints, being either underweight, overweight, or obese, and experiencing physical limitations. Of note, feeling fatigued may reduce physical activity, but regular physical activity may in its turn reduce cancer‐related fatigue.^51^, ^53^ Female sex was the most commonly identified risk factor associated with lower levels of physical activity, followed by a treatment history including radiotherapy, having children, having less education, and being of Hispanic, black, or other non‐Hispanic ethnicity. Facilitators for physical activity included perceived health benefits and levels of motivation. Higher education was the only supportive factor associated with increased physical activity in at least two studies.

Systematic reviews in people without cancer found comparable correlates of physical activity, including sex, having knowledge/appreciation of the benefits of physical activity, (lack of) motivation, smoking, access to facilities, lack of time, lack of energy, and having underlying health problems.^54^, ^55^, ^56^, ^57^ Besides, Brown and colleagues recently synthesized evidence from eight qualitative studies of barriers and facilitators to physical activity from the perspective of childhood cancer survivors.^58^ Parental influence and support were found to be major themes, possibly because parental factors were the main focus of two of the included studies. The current review adds to these findings by synthesizing evidence from both qualitative and quantitative studies, including the impact of sociodemographic, cancer and treatment‐related factors on survivors' physical activity levels.

Higher smoking rates among CAYA cancer survivors were related to lower levels of education, poor mental health, and having more peers or household members who smoke. In contrast, increased energy was associated with lower smoking rates. Men were more likely than women to have higher levels of alcohol consumption. These findings are consistent with the literature on smoking and alcohol consumption in the general population^59^, ^60^, ^61^ and highlight the importance of sociodemographic factors such as sex and educational level in identifying those at risk of unhealthy behaviors. Furthermore, as smoking and mental health are linked through the withdrawal effect of tobacco, HCPs can explain to smokers that the decrease in nicotine levels after smoking a cigarette leads to withdrawal symptoms such as poor concentration, insomnia, feelings of tension, restlessness, low mood, and anxiety.^60^ One strategy that can be used to support smoking cessation is cognitive behavioral therapy (CBT), which helps people understand the relationships between their thoughts, feelings, behaviors, and physical experiences.^60^ CBT can also be used for improving other health behaviors.

This review also found that women were more likely than men to adhere to sun exposure recommendations, with the exception of occasional sun exposure. This is consistent with research in the general population suggesting that men are more likely to perceive the inconvenience and cost of sunscreen and sun‐protective clothing as barriers to their sun‐protective behavior.^62^ In addition, men tend to perceive skin damage from sun exposure as less severe than women do.^62^

We did not find any barriers, facilitators, or other factors associated with a healthy diet that were identified by two or more studies. In the general population, systematic reviews found that social environment plays an important role in dietary health behavior, along with automaticity, self‐regulation, motivational regulation, subjective norm, and relationships with sedentary behavior.^63^, ^64^ However, the evidence is suggestive at best, because of the widespread use of cross‐sectional designs in the studies included in the reviews. More research is therefore needed to understand the barriers and facilitators associated with a healthy diet among both the general population and, particularly, CAYA cancer survivors.

### 4.1 Health behavior interventions and identification of survivors most at risk

The barriers and facilitators identified in this review can be used as a starting point for developing health behavior interventions that meet the needs and preferences of individual CAYA cancer survivors and support them in adopting healthier lifestyles. For example, a targeted health behavior intervention can help survivors to manage their clinical symptoms of fatigue and time constraints, and increase their motivation by addressing their individual preferences and needs and by emphasizing the benefits of healthy lifestyles. A recent systematic review and meta‐analysis on healthy lifestyle interventions found that current health behavior interventions are primarily exercise‐based without significant effects on physical outcomes such as physical fitness, fatigue, and body mass index.^65^ Therefore, a different, more holistic and individualized approach to health behavior interventions is warranted.^66^ Overall, further clinical trials are needed to increase the body of research on effective health behavior interventions for CAYA cancer survivors. Such interventions should build on the accumulated evidence on barriers and facilitators and address strategies to overcome fatigue, increase and sustain motivation over time, and include aspects of time management techniques. Moreover, engaging key stakeholders such as survivors, HCPs, and policymakers at the initial stages of intervention development increases the likelihood of creating interventions that are not only delivered on time and within budget but also deemed acceptable and feasible.^67^

The insights in relevant risk and supportive factors associated with health behaviors can help to increase awareness among HCPs regarding which survivors are most at risk of certain unhealthy behaviors. However, the consistent lack of statistical significance observed for risk and supportive factors related to cancer history and treatment, such as age at diagnosis, cancer diagnosis, and cancer treatment, highlights the possibility that these specific factors may not be of substantial importance in relation to health behaviors in CAYA cancer survivors. In other words, this review indicates that the primary results identified are not inherently specific to CAYA cancer survivors. Consequently, HCPs and lifestyle coaches may need to broaden their focus beyond medical history when assisting CAYA cancer survivors to improve their health behaviors and adopt new habits. For instance, other individual characteristics such as sex and educational attainment should be taken into account.

### 4.2 The importance of knowledge dissemination

This review highlights the importance of increasing knowledge about healthy behaviors in general among CAYA cancer survivors through health behavior advice, information dissemination, and health promotion discussions with HCPs. These findings align with a recent qualitative study of HCPs, which highlighted the critical role of education and training of HCPs in effectively guiding CAYA cancer survivors toward healthy behaviors.^68^ Survivorship care clinics should prioritize the integration of health behavior support services such as lifestyle coaching and ensure that HCPs are adequately equipped with the necessary knowledge and skills to support survivors in adopting and maintaining healthy behaviors. In addition, a systematic review of 17 randomized controlled trials among all types of patients showed that using deliberate communication strategies when providing information can improve patient outcomes more effectively than not using such strategies.^69^ Therefore, when HCPs aim to encourage survivors to engage in specific health behaviors, they may particularly benefit from using explicit persuasive information strategies.

### 4.3 Strengths and limitations

The methodology used in this review had several strengths. First, we followed a rigorous and transparent approach, including a comprehensive search strategy and the involvement of two independent reviewers in the screening of studies and data extraction. We included both quantitative and qualitative studies to enrich the scope and depth of our review. However, this review brings together very different study designs and methodologies such as studies reporting on survivors' perceived influences on behavior and cohort studies reporting on risk factors. The results should therefore be interpreted with caution and used as a starting point to develop health behavior interventions and identify survivors most at risk of unhealthy behaviors. Furthermore, our strict inclusion criteria limited the age range of participants to 16–50 years. As a result, we might have missed relevant findings from studies that included participants outside this age range and AYA cancer survivors with an adult cancer diagnosis. This may somewhat limit the generalizability of our conclusions. Nevertheless, our findings are still valuable for understanding the barriers and facilitators that may promote healthy behaviors in CAYA cancer survivors.

## 5 CONCLUSION

Our comprehensive review examined different aspects of health behavior, including physical activity, smoking, diet, alcohol consumption, sun exposure, and health behavior in general. Barriers, such as fatigue, unmet information needs, time constraints, lack of motivation, social influences, poor mental health, and facilitators, such as the need for lifestyle advice and health promotion discussions with HCPs, highlight the importance of targeted interventions. The identification of other factors associated with health behavior outcomes, including (among others) sex and educational attainment, highlights the need to consider individual context and sociodemographic characteristics. Overall, our findings can be used as a starting point for the development of more targeted and effective health behavior change interventions to promote healthy behaviors in CAYA cancer survivors, to support them in adapting these behaviors, and to inform lifestyle coaches. Knowledge of other factors can be used to raise awareness among HCPs of which survivors are most at risk of unhealthy behaviors.

## AUTHOR CONTRIBUTIONS

**Ismay A. E. de Beijer:** Conceptualization (equal); data curation (equal); formal analysis (equal); methodology (equal); project administration (equal); visualization (lead); writing – original draft (lead). **Eline Bouwman:** Conceptualization (equal); formal analysis (equal); methodology (equal); writing – review and editing (lead). **Renée L. Mulder:** Methodology (equal); supervision (equal); writing – review and editing (equal). **Philippa Steensma:** Methodology (equal). **Morven C. Brown:** Conceptualization (equal); funding acquisition (supporting); methodology (equal); writing – review and editing (equal). **Vera Araújo‐Soares:** Conceptualization (equal); funding acquisition (supporting); methodology (equal); writing – review and editing (equal). **Magdalena Balcerek:** Methodology (equal); writing – review and editing (equal). **Edit Bardi:** Methodology (equal); writing – review and editing (equal). **Jeanette Falck‐Winther:** Funding acquisition (equal); methodology (equal); writing – review and editing (equal). **Line Elmerdahl Frederiksen:** Methodology (equal); writing – review and editing (equal). **Marloes van Gorp:** Methodology (equal); writing – review and editing (equal). **Sara Oberti:** Methodology (equal); writing – review and editing (equal). **Rebecca J. van Kalsbeek:** Methodology (equal); writing – review and editing (equal). **Tomas Kepak:** Funding acquisition (equal); methodology (equal); writing – review and editing (equal). **Katerina Kepakova:** Methodology (equal); writing – review and editing (equal). **Hannah Gsell:** Methodology (equal); writing – review and editing (equal). **Anita Kiesinger:** Methodology (equal); writing – review and editing (equal). **Raphaële van Litsenburg:** Methodology (equal); writing – review and editing (equal). **Luzius Mader:** Methodology (equal); writing – review and editing (equal). **Gisela Michel:** Funding acquisition (equal); methodology (equal); writing – review and editing (equal). **Monica Muraca:** Funding acquisition (equal); methodology (equal); writing – review and editing (equal). **Selina R. van den Oever:** Methodology (equal); writing – review and editing (equal). **Helena J. H. van der Pal:** Funding acquisition (equal); methodology (equal); writing – review and editing (equal). **Katharina Roser:** Methodology (equal); writing – review and editing (equal). **Roderick Skinner:** Funding acquisition (equal); methodology (equal); writing – review and editing (equal). **Iridi Stolman:** Methodology (equal); writing – review and editing (equal). **Anne Uyttebroeck:** Funding acquisition (equal); methodology (equal); writing – review and editing (equal). **Leontien C. M. Kremer:** Conceptualization (supporting); funding acquisition (lead); methodology (equal); supervision (equal); writing – review and editing (equal). **Jacqueline Loonen:** Conceptualization (lead); funding acquisition (equal); methodology (lead); supervision (lead); writing – review and editing (lead). **Elvira C. van Dalen:** Conceptualization (lead); methodology (lead); supervision (lead); writing – review and editing (lead). **Saskia M. F. Pluijm:** Conceptualization (lead); formal analysis (lead); funding acquisition (lead); methodology (lead); supervision (lead); writing – original draft (equal); writing – review and editing (lead).

## FUNDING INFORMATION

This work was supported by the European Union's Horizon 2020 Framework Program (Grant Number 824982). The funder had no role in study design, data col‐lection, data analysis, data interpretation, or in writing the report. The material presented and views expressed here are the responsibility of the author(s) only. The EU Commission takes no responsibility for any use made of the information set out.

## CONFLICT OF INTEREST STATEMENT

The authors declare that they have no known competing financial interests or personal relationships that could have appeared to influence the work reported in this paper.

## Supporting information


Appendix S1.

Appendix S2.

## Data Availability

Data sharing is not applicable to this article as no new data were created or analyzed in this study.

## APPENDIX A Members PanCare FollowUp Consortium

### A.1

Leontien C.M. Kremer, Princess Máxima Center for Paediatric Oncology, Hei delberglaan 25, 3584 CS, Utrecht, the Netherlands; Department of Paediatrics, Emma Children's Hospital, Amsterdam UMC, Meibergdreef 9, 1105 AZ Amsterdam, the Netherlands; Faculty of Medicine, Utrecht University and Utrecht Medical Center, Universiteitsweg 98, 3584 CG Utrecht, the Netherlands.

Helena J.H. van der Pal, Princess Máxima Center for Paediatric Oncology, Heidelberglaan 25, 3584 CS, Utrecht, the Netherlands; PanCare, Jacobus Bellamylaan 16, 1401 AZ Bussum, the Netherlands.

Renée L. Mulder, Princess Máxima Center for Paediatric Oncology, Heidelberg laan 25, 3584 CS, Utrecht, the Netherlands.

Saskia M.F. Pluijm, Princess Máxima Center for Paediatric Oncology, Heidelberglaan 25, 3584 CS, Utrecht, the Netherlands. Rebecca J. van Kalsbeek, Princess Máxima Center for Paediatric Oncology, Heidelberglaan 25, 3584 CS, Utrecht, the Netherlands.

Selina R. van den Oever, Princess Máxima Center for Paediatric Oncology, Heidelberglaan 25, 3584 CS, Utrecht, the Netherlands.

Lars Hjorth, Lund University, Skane University Hospital, Department of Clinical Sciences Lund, Paediatrics, Lasarettsgatan 40, 221 85 Lund, Sweden.

Cecilia Follin, Lund University, Skane University Hospital, Department of Clini cal Sciences Lund, Oncology, Lasarettsgatan 40, 221 85 Lund, Sweden.

Lill Eriksson, Lund University, Skane University Hospital, Department of Clinical Sciences Lund, Oncology, Lasarettsgatan 40, 221 85 Lund, Sweden.

Thomas Relander, Lund University, Skane University Hospital, Department of Clinical Sciences Lund, Oncology, Lasarettsgatan 40, 221 85 Lund, Sweden.

Jacob Engellau, Lund University, Skane University Hospital, Department of Clinical Sciences Lund, Oncology, Lasarettsgatan 40, 221 85 Lund, Sweden.

Karin Fjordén, Lund University, Skane University Hospital, Department of Clini cal Sciences Lund, Oncology, Lasarettsgatan 40, 221 85 Lund, Sweden.

Karolina Bogefors, Lund University, Skane University Hospital, Department of Clinical Sciences Lund, Oncology, Lasarettsgatan 40, 221 85 Lund, Sweden.

Anna S. Holmqvist, Lund University, Skane University Hospital, Department of Clinical Sciences Lund, Paediatrics, Lasarettsgatan 40, 221 85 Lund, Sweden.

Riccardo Haupt, Epidemiology and Biostatistics Unit and DOPO clinic, IRCCS Istituto Giannina Gaslini, Via G. Gaslini, 5, 16147 Genoa, Italy.

Monica Muraca, Epidemiology and Biostatistics Unit and DOPO clinic, IRCCS Istituto Giannina Gaslini, Via G. Gaslini, 5, 16147 Genoa, Italy.

Brigitte Nicolas, Epidemiology and Biostatistics Unit and DOPO clinic, IRCCS Istituto Giannina Gaslini, Via G. Gaslini, 5, 16147 Genoa, Italy.

Francesca Bagnasco, Epidemiology and Biostatistics Unit and DOPO clinic, IRCCS Istituto Giannina Gaslini, Via G. Gaslini, 5, 16147 Genoa, Italy.

Marina Benvenuto, Epidemiology and Biostatistics Unit and DOPO clinic, IRCCS Istituto Giannina Gaslini, Via G. Gaslini, 5, 16147 Genoa, Italy.

Anna Aulicino, Epidemiology and Biostatistics Unit and DOPO clinic, IRCCS Istituto Giannina Gaslini, Via G. Gaslini, 5, 16147 Genoa, Italy.

Luca Laudisi, Epidemiology and Biostatistics Unit and DOPO clinic, IRCCS Istituto Giannina Gaslini, Via G. Gaslini, 5–16147 Genoa, Italy.

Vera Araújo‐Soares, Center for Preventive Medicine and Digital Health, Department for Prevention, Medical Faculty Mannheim, Heidelberg University, Röntgenstraße 7 D‐68167, Mannheim, Germany.

Tomas Kepak, International Clinical Research Center, St. Anne's University Hospital Brno, Pekařská 53, Brno 656 91, Czech Republic (FNUSA‐ICRC). Katerina Kepakova, International Clinical Research Center, at St. Anne's University Hospital, Masaryk University, Pekařská 53, Brno 656 91, Czech Republic (FNUSA‐ICRC).

Hana Hrstkova, International Clinical Research Center, St. Anne's University Hospital Brno, Pekařská 53, Brno 656 91, Czech Republic (FNUSA‐ICRC).

Viera Bajciova, International Clinical Research Center, St. Anne's University Hospital Brno, Pekařská 53, Brno 656 91, Czech Republic (FNUSA‐ICRC).

Marta Holikova, International Clinical Research Center, St. Anne's University Hospital Brno, Pekařská 53, Brno 656 91, Czech Republic (FNUSA‐ICRC). Lucie Strublova, International Clinical Research Center, St. Anne's University Hospital Brno, Pekařská 53, Brno 656 91, Czech Republic (FNUSA‐ICRC).

Anne Uyttebroeck, Department of Oncology, Paediatric Oncology, KU Leuven, Department of Paediatric Haematology and Oncology, University Hospitals Leuven, Herestraat 49, 3000 Leuven, Belgium.

Marleen Renard, Department of Paediatric Haematology and Oncology, University Hospitals Leuven, Herestraat 49, 3000 Leuven, Belgium.

Sandra Jacobs, Department of Oncology, Paediatric Oncology, KU Leuven, Department of Paediatric Haematology and Oncology, University Hospitals Leuven, Herestraat 49, 3000 Leuven, Belgium. Heidi Segers, Department of Oncology, Paediatric Oncology, KU Leuven, Department of Paediatric Haematology and Oncology, University Hospitals Leuven, Herestraat 49, 3000 Leuven, Belgium. Monique van Helvoirt, Department of Paediatric Haematology and Oncology, University Hospitals Leuven, Herestraat 49, 3000 Leuven, Belgium.

Jeanette F. Winther, Childhood Cancer Research Group, Danish Cancer Society Research Center, Strandboulevarden 49, 2100 Copenhagen, Denmark; Department of Clinical Medicine, Faculty of Health, Aarhus University and Aarhus University Hospital, Palle Juul‐Jensens Boulevard 82, 8200 Aarhus, Denmark.

Luzius Mader, Childhood Cancer Research Group, Danish Cancer Society Research Center, Strandboulevarden 49, 2100 Copenhagen, Denmark; Institute of Social and Preventive Medicine, University of Bern, Mittelstrasse 43, 3012 Bern, Switzerland.

Line E. Frederiksen, Childhood Cancer Research Group, Danish Cancer Society Research Center, Strandboulevarden 49, 2100 Copenhagen, Denmark.

Elisabeth A. W. Andersen, Statistics and Data Analysis, Danish Cancer Society Research Center, Strandboulevarden 49, 2100 Copenhagen, Denmark.

Gisela Michel, University of Lucerne, Faculty of Health Sciences and Medicine, Alpenquai 4, 6005 Lucerne, Switzerland.

Stefan Boes, University of Lucerne, Faculty of Health Sciences and Medicine, Alpenquai 4, 6005 Lucerne, Switzerland.

Katharina Roser, University of Lucerne, Faculty of Health Sciences and Medicine, Alpenquai 4, 6005 Lucerne, Switzerland.

Jacqueline Loonen, Radboud University Medical Center, Radboud Institute for Health Sciences, Department of Hematology, Geert Grooteplein Zuid 10, 6525 GA, Nijmegen, the Netherlands.

Rosella Hermens, Radboud University Medical Center, Radboud Institute for Health Sciences, Scientifc Institute for Quality of Healthcare (IQ Healthcare), Geert Grooteplein 21, 6525 EZ, Nijmegen, the Netherlands.

Irene Göttgens, Radboud University Medical Center, Radboud Institute for Health Sciences, Department of Primary and Community Care, Geert Grooteplein 21, 6525 EZ, Nijmegen, the Netherlands.

Eline Bouwman, Radboud University Medical Center, Radboud Institute for Health Sciences, Department of Hematology, Geert Grooteplein Zuid 10, 6525 GA, Nijmegen, the Netherlands.

Iridi Stollman, Radboud University Medical Center, Radboud Institute for Health Sciences, Department of Hematology, Geert Grooteplein Zuid 10, 6525 GA, Nijmegen, the Netherlands. Adriaan Penson, Radboud University Medical Center, Radboud Institute for Health Sciences, Department of Hematology, Geert Grooteplein Zuid 10, 6525 GA, Nijmegen, the Netherlands.

Dionne Breij, Radboud University Medical Center, Radboud Institute for Health Sciences, Department of Hematology, Geert Grooteplein Zuid 10, 6525 GA, Nijmegen, the Netherlands.

Roderick Skinner, Newcastle University Centre for Cancer, Wolfson Childhood Cancer Research Centre, Herschel Building, Brewery Lane, Newcastle upon Tyne, NE1 7RU, United Kingdom; Great North Children's Hospital, Royal Victoria Infrmary, Queen Victoria Road, Newcastle upon Tyne, NE1 4 LP, United Kingdom; Translational and Clinical Research Institute, Wolfson Childhood Cancer Research Centre, Herschel Building, Brewery Lane, Newcastle upon Tyne, NE1 7RU, United Kingdom. Morven C. Brown, Population Health Sciences Institute, Newcastle University, Sir James Spence Institute, Royal Victoria Infrmary, Queen Victoria Road, Newcastle upon Tyne, NE1 4LP, United Kingdom; Newcastle University Centre for Cancer, Wolfson Childhood Cancer Research Centre, Herschel Building, Brewery Lane, Newcastle upon Tyne, NE1 7RU, United Kingdom.

Samira Essiaf, European Society for Paediatric Oncology, c/o BLSI, Clos Chapelle‐aux‐Champs 30, Bte 1.30.30, BE‐1200 Brussels, Belgium.

Anne Blondeel, European Society for Paediatric Oncology, c/o BLSI, Clos Chapelle‐aux‐Champs 30, Bte 1.30.30, BE‐1200 Brussels, Belgium.

William Sciberras, European Society for Paediatric Oncology, c/o BLSI, Clos Chapelle‐aux‐Champs 30, Bte 1.30.30, BE‐1200 Brussels, Belgium.

Joke Korevaar, Netherlands Institute for Health Services Research (Nivel), P.O. Box 1568, 3500 BN Utrecht, the Netherlands.

Mieke Rijken, Netherlands Institute for Health Services Research (Nivel), P.O. Box 1568, 3500 BN Utrecht, the Netherlands; University of Eastern Finland, Department of Health and Social Management, P.O. Box 1627, FI‐70211 Kuopio, Finland.

Anita Kienesberger, Childhood Cancer International Europe, Servitengasse 5/16, 1090 Vienna, Austria.

Jaap den Hartogh, Princess Máxima Center for Paediatric Oncology, Heidelberglaan 25, 3584 CS, Utrecht, the Netherlands.

Hannah Gsell, Childhood Cancer International Europe, Servitengasse 5/16, 1090 Vienna, Austria. Carina Schneider, Childhood Cancer International Europe, Servitengasse 5/16, 1090 Vienna, Austria. Edit Bardi, PanCare, Jacobus Bellamylaan 16, 1401 AZ Bussum, the Netherlands.

Jeroen te Dorsthorst, PanCare, Jacobus Bellamylaan 16, 1401 AZ Bussum, the Netherlands.

## APPENDIX B Search strategy for barriers, facilitators, factors and effectiveness of eHealth lifestyle interventions

### B.1

Search Strategy for OVID‐Medline and PsycInfo (adapted for PubMed update):

**Table:**

1	“cochrane review e‐health facilitators and barriers”.ti.
2	exp nervous system neoplasms
3	leukemias/
4	(leukemia or leukemi* or leukaemi*).tw,id.
5	(aml or anll or lymphoma or lymphom* or hodgkin* or T‐cell or B‐cell or non‐hodgkin or sarcoma or sarcom* or Ewing* or osteosarcom* or wilms* or nephroblastom* or neuroblastom* or rhabdomyosarcom* or teratom* or hepatom* or hepatoblastom* or PNET or medulloblastom* or PNET* or (neuroectodermal adj2 tumors adj2 primitive) or retinoblastoma or retinoblastom* or meningiom* or gliom*).tw,id.
6	exp neoplasms/
7	((brain adj tumo?r*) or (brain adj neoplasm?) or (central adj nervous adj system adj neoplasm?) or (central adj nervous adj system adj tumo?r?) or (central adj nervous adj system adj cancer?) or (brain adj cancer*) or (brain adj neoplasm*) or (intracranial adj neoplasm*) or (leukemia adj lymphocytic adj acute*)).tw,id.
8	or/2‐7
9	“P variant neurocognief”.ti.
10	(neoplasm* or hemato?oncolog* or (hemato adj oncological)).tw,id.
11	malignan*.tw,id.
12	(tumour* or tumor*).tw,id.
13	cancer*.tw,id.
14	carcinoma*.tw,id.
15	leuk?emia*.tw,id.
16	oncolog*.tw,id.
17	or/10‐16
18	“P variant breed”.ti.
19	((p?ediatric adj3 oncolog*) or (child* adj3 (cancer? or tumo?r? or neoplasm?))).tw,id.
**20**	**8 or 17 or 19 =pediatric oncology**
21	((late? adj3 effect*) or (long adj3 term) or long?term or (later adj3 side effect*)).id,tw.
22	survivors/ or symbolic interactionism/
23	(surviv* or survivor? or survival?).ti,id.
24	aftercare/ or “continuum of care”/ or exp maintenance therapy/ or exp outpatient treatment/ or partial hospitalization/ or posttreatment followup/
**25**	**or/21‐24 = survivors late effects**
26	exp lifestyle/ or exp health behaviour/
27	(lifestyle? or (life adj2 style?)).tw,id.
28	health promotion/
29	client education/ or exp health education/
30	weight control/ or exp exercise/ or food intake/ or “obesity (attitudes toward)”/ or weight gain/ or weight loss/
31	sedentary behaviour/
32	tobacco smoking/ or smoking cessation/
33	exp alcohol drinking patterns/ or drinking behaviour/ or exp alcoholism/
34	exercise/ or physical activity/
35	exp sports/ or swimming/
36	treatment compliance/
37	((smoking adj3 cessat*) or nutrition or diet* or self‐care or (dietary adj3 chang*) or (weight adj3 control*) or (stimulat* adj3 physical)).tw,id.
38	((body adj2 weight adj3 maintena*) or exercis* or walking or training or smoking or (physical adj3 exercis*) or diet or alcohol or eating).tw,id.
39	((weight adj3 loss) or overweight or obesit* or (dietary adj3 intake)).tw,id.
40	(fruit or vegetable? or nutrition or smoking or alcohol or self?help or self‐care or (self adj help) or (self adj care)).tw,id.
41	self‐management/
42	exp motor performance
43	((behavio?r or life?style or (life adj style)) adj3 (change or intervent* or counsel*)).tw,id.
44	diets/ or exp food/
45	exp cognitive behaviour therapy/
46	acceptance.mp. and commitment therapy.tw,id. [mp=title, abstract, heading word, table of contents, key concepts, original title, tests & measures, mesh]
47	mindfulness/ or mindfulness‐based interventions/
48	exp relaxation therapy/
49	motivational interviewing/ or exp behaviour change/
50	counseling/
51	exp behaviour therapy/
52	(CBT or (cognitive adj3 behavi??r adj3 therap*) or (motivational adj3 interview*) or (commitment adj therap*) or (behavi??r adj3 interventi*)).tw,id.
**53**	**or/26‐52 = life style**
54	20 and 25 and 53
55	((need? adj3 demand*) or (need? adj3 assess*) or tailor* or (patient adj3 cent*) or personali* or facilitat* or barrier*).tw.
56	((need? adj3 demand*) or (need? adj3 assess*) or tailor* or (patient adj3 cent*) or personali* or facilitat* or barrier*).id.
57	health care services/ or exp electronic health services/ or exp health care delivery/ or exp hospital programs/ or long term care/ or exp mental health services/ or exp health care seeking behaviour/ or health service needs/ or exp managed care/
58	(attitude? or need? or prefer*).ti,id.
59	attitudes/ or health attitudes/
60	treatment compliance/ or treatment barriers/
**61**	**or/55‐60=facilitators and barriers**
62	54 and 61
63	“onderdeel ehealth”.ti.
64	internet/ or blog/ or electronic collaboration/ or exp electronic communication/ or online experiments/ or online therapy/ or exp social media/ or exp telemedicine/ or exp websites/ or exp wireless technologies/ or internet usage/
65	telerehabilitation/ or rehabilitation counseling/ or videoconferencing/
66	telemetry/
67	(telemetr* or (rehabilitation adj3 (remote or virtual or tele)) or telemedicine).tw,id.
68	(mhealth or ehealth or telehealth or telemedicine or (mobile adj health) or telerehabilitation).tw,id. (
69	(web?based or online).tw,id.
70	(online adj3 (coach* or support* or platform or environ*)).tw,id.
71	(web adj3 (coach* or support* or platform or environ*)).tw,id.
72	(online adj3 (support* or self*)).tw,id.
73	(internet adj3 (support or self*)).tw,id.
**74**	**or/64‐73 =eHealth**
75	54
76	limit 75 to (all journals and english language)
**77**	**20 and 25 and 53 and 61 = barriers, facilitators and factors**
78	english.la.
79	20 and 25 and 74
80	20 and 25 and 53 and 61
81	80 and 78
82	limit 81 to all journals
**83**	**20 and 25 and 53 and 74 = effectiveness of eHealth lifestyle interventions.**
84	83 and 78
85	limit 84 to all journals

Abbreviations: Id = article identifier, Ti = title, Tw = text word, La = language.
