# Supplementary material for: Barriers, facilitators, and other factors associated with health behaviors in childhood, adolescent, and young adult cancer survivors: A systematic review
Source: Cancer Med. 2024 Jun 21;13(12):e7361. doi: 10.1002/cam4.7361 (PMC11192647; doi:10.1002/cam4.7361)
Supplement: Supplementary file 1 — Appendix S1. [file CAM4-13-e7361-s002.docx]

| ***Zwemer et al.*** Sun exposure in young adult cancer survivors on and off the beach: results from Project REACH. J Cancer Surviv, 2012, 6(1), 63–71. | | | |
| --- | --- | --- | --- |
| **Study design**  **Treatment era**  **Years of follow-up** | **Participants** | **Variable definitions** | **Results** |
| Study design  Cross-sectional study  Treatment era  Not reported  Follow-up duration  Years from cancer diagnosis:   - 2–10 years: N=42 (27.5%) - 11–15 years: N=36 (23.5%) - 16–20 years: N=24 (22.2%) - 20+ years: N=41 (26.8%) | Type and number of participants  N=153 adults diagnosed with non-skin cancer before age 30 years  Cancer diagnosis   - Central nervous system: N=45 (29.4%) - Hodgkin’s lymphoma: N=30 (19.6%) - Leukaemia: N=25 (16.3%) - Bone: N=15 (9.8%) - Non-Hodgkin’s lymphoma: N=11 (7.2%) - Sarcoma: N=9 (5.9%) - Wilms tumour: N=5 (3.3%) - Neuroblastoma: N=5 (3.3%) - Germ cell tumour: N=5 (3.3%) - Retinoblastoma: N=2 (1.3%) - Hepatoblastoma: N=1 (0.7%)   Age at diagnosis   - 0-5 years: N=39 (25.4%) - 6-11 years: N=24 (15.7%) - 12-17 years: N=65 (42.5%) - 18-29 years: N=25 (16.3%)   Age at follow-up   - 18–25 years: N=76 (49.7%) - 26–35 years: N=48 (31.4%) - 36–45 years: N=22 (14.4%) - 46–60 years: N=7 (4.6%)   Cancer treatment  *History of radiation therapy*   - No radiation: N=51 (33.3%) - Radiation: N=102 (66.7%)   Controls/normal population (if applicable)  Not applicable  Additional participant characteristics (if applicable):  Not applicable | Sun exposure measures  *Sun exposure*  Measured with the Sun Survey (15 sun exposure and protection items). Sunbathing was defined as “time you spend in the sun with the goal of ‘getting some colour.’” Incidental sun exposure was defined as “time in the sun doing something other than sunbathing. SB and IE exposure and protection were assessed by asking about typical behaviours during the 2009 summer months, including amounts of sun exposure, sunscreen use on face and body, and hat wearing during IE. Separate items for weekends and weekdays were  summed to derive weekly average totals. One item asked about frequency of artificial tanning over the past year.  Other variables  *Perceived vulnerability to UV exposure*  Six items assessed vulnerability to appearance changes (e.g., wrinkling, looking older) due to UV exposure. Three parallel items were developed for this survey to assess perceived vulnerability to future skin cancers due to UV exposure. Items were Z-scored and averaged to create separate scores reflecting PV to appearance changes and PV to skin cancer.  *Perceived skin cancer risk*  One item asking participants to rate their overall risk of skin cancer compared to the general population: “Compared to most people, I think the chances that I will get skin cancer someday are…” on a five-point scale from “much lower than average” to “much higher than average.” Ratings were collapsed to three categories (higher, the same, or lower than average) for analysis. | Associations between factors and sun exposure  Risk factors for low adherence to sunbathing and incidental sun exposure recommendations. Odds ratios are adjusted for age and gender.  Sunbathing adherence  None of the results were found to be significant unless indicated.  *Current age (18-25 years = ref.)*  26-60 years: OR = 0.41, 95% CI (0.18–0.91), p = sign. (no specific values reported)  *Age at diagnosis (0-5 years = ref.)*   - 6-11 years: OR=0.92, 95% CI (0.28–3.06) - 12-17 years: OR= 1.41, 95% CI (0.57–3.46) - 18-29 years: OR= 1.12, 95% CI (0.30–4.22)   *Years from diagnosis (2-10 years = ref.)*   - 11-15 years: OR = 0.60, 95% CI (0.19–1.87) - 16-20 years: OR= 3.37, 95% CI (1.18–9.69) - 20+ years: OR= 0.96, 95% CI (0.27–3.44)   *Gender (male = ref.)*  Female: OR = 2.44, 95% CI (1.10–5.45), p = sign. (no specific values reported)  *Education (post-graduate degree = ref.)*   - Attended college: OR = 0.62, 95% CI (0.20–1.94) - Less than college: OR = 0.31, 95% CI (0.07–1.30)   *Household income (≥$100,000 = ref.)*   - $75,000–99,999: OR = 0.38, 95% CI (0.12–1.25) - $50,000–74,999: OR = 0.67, 95% CI (0.22–2.06) - $30,000–49,999: OR = 0.65, 95% CI (0.19–2.22) - <$30,000: OR = 0.88, 95% CI (0.28–2.80)   *Currently employed (full-time = ref.)*   - Part-time: OR = 1.21, 95% CI (0.45–3.28) - Not working: OR = 0.44, 95% CI (0.16–1.19)   *Currently in school (no = ref.)*  Yes: OR = 2.05, 95% CI (0.79–5.30)  *Race/ethnicity (Caucasian = ref.)*  Non-Caucasian: OR = 1.90, 95% CI (0.53–6.83)  *Cancer diagnosis (lymphoma = ref.)*   - Leukaemia: OR = 0.63, 95% CI (0.19–2.01) - Central nervous system: OR = 0.42, 95% CI (0.14–1.24) - Other solid tumours: OR = 0.68, 95% CI (0.22–1.67)   *History of radiation (no radiation = ref.)*  Radiation: OR = 1.54, 95% CI (0.68–3.44)  *Skin cancer risk (higher than most = ref.)*  Average or less than most: OR = 0.82, 95% CI (0.39–1.74)  *Perceived vulnerability to appearance changes (no ref.):*  OR *=* 1.21, 95% CI (0.73–2.03)  *Perceived vulnerability to skin cancer (no ref.):*  OR = .95, 95% CI (0.60–1.49)  Incidental exposure adherence  *Current age (18-25 years = ref.)*  26–60 years: OR = 0.50, 95% CI (0.24–1.01)  *Age at diagnosis (0-5 years = ref.)*   - 6–11 years: OR = 1.22, 95% CI (0.40–3.70) - 12–17 years: OR = 1.47, 95% CI (0.61–3.55) - 18–29 years: OR = 0.22, 95% CI (0.04–1.19)   *Years from diagnosis (2-10 years = ref.)*   - 11–15 years: OR = 1.10, 95% CI (0.41–2.92) - 16–20 years: OR = 0.94, 95% CI (0.34–2.65) - 20+ years: OR = 1.59, 95% CI (0.47–5.32)   *Gender (male = ref.)*  Female: OR = 0.51, 95% CI (0.25–1.04)  *Education (post-graduate degree = ref.)*   - Attended college: OR = 1.02, 95% CI (0.32–3.20) - Less than college: OR = 0.58, 95% CI (0.14–2.37)   *Household income (≥$100,000 = ref.)*   - $75,000–99,999: OR = 2.60, 95% CI (0.80–8.41) - $50,000–74,999: OR = 1.45, 95% CI (0.47–4.45) - $30,000–49,999: OR = 3.54, 95% CI (1.07–11.75) - <$30,000: OR = 1.07, 95% CI (0.30–3.77)   *Currently employed (full-time = ref.)*   - Part-time: OR = 0.64, 95% CI (0.21–1.90) - Not working: OR = 1.43, 95% CI (0.58–3.52)   *Currently in school (no = ref.)*  Yes: OR = 0.93, 95% CI (0.38–2.27)  *Race/ethnicity (Caucasian = ref.)*  Non-Caucasian: OR = 0.52, 95% CI (0.10–2.64)  *Cancer diagnosis (lymphoma = ref.)*   - Leukaemia: OR = 2.92, 95% CI (0.91–9.34) - Central nervous system: OR = 2.29, 95% CI (0.78–6.70) - Other solid tumours: OR = 0.81, 95% CI (0.26–2.51)   *History of radiation (no radiation = ref.)*  Radiation: OR = 0.77, 95% CI (0.36–1.65)  *Skin cancer risk (higher than most = ref.)*  Average or less than most: OR = 1.46, 95% CI (0.69–3.08)  *Tanning booth in last year (no = ref.)*  Yes: OR = 1.08, 95% CI (0.36–3.23)  *Perceived vulnerability to appearance changes (no ref.):*  Yes: OR *=* 0.55, 95% CI(0.34–0.90)  *Perceived vulnerability to skin cancer (no ref.)*  OR = 1.11, 95% CI (0.71–1.74)  Within the years from diagnosis and household income variables, individual strata differed within either SB or IE adherence, but no consistent pattern of relationship  with adherence groups was identified. |

Abbreviations: CI, confidence interval, CNS, central nervous system, IE, incidental exposure, OR, odds ratio, SB, sun bathing

| ***Zhang et al.*** Adult Survivors of Childhood Cancer Have Poor Adherence to Dietary Guidelines, J of Nutr ,2016, 146(12), 2497–2505. | | | |
| --- | --- | --- | --- |
| **Study design**  **Treatment era**  **Years of follow-up** | **Participants** | **Variable definitions** | **Results** |
| Study design  Retrospective cohort study with cross-sectional assessment  Treatment era  Not reported  Follow-up duration  Mean time from diagnosis = 24.1 years (SD 8.1) | Type and number of participants  N=2570 childhood cancer survivors  Cancer diagnosis   - Leukaemia: N=986 (38.4%) - Lymphoma: N=497 (19.4%) - Embryonal tumour: N=335 (13.1%) - Sarcoma: N=328 (12.8%) - CNS tumour: N=239 (9.3%) - Other: N=181 (7.1%)   Age at diagnosis  Mean age = 8.3 years (SD 5.6)  Age at follow-up  Mean age = 32.3 years (SD 8.3)  Cancer treatment   - Alkylating agents: N=1,627 (63.3%) - Anthracyclines: N=1499 (58.3%) - Antimetabolites: N=1369 (53.3%) - Glucocorticoids: N=1260 (49.0%) - Radiation (any): N=1532 (59.6%)   To brain: N=916 (35.6%)  To abdomen: N=588 (22.9%)  Controls/normal population (if applicable)  Not applicable  Additional participant characteristics (if applicable):  Not applicable | Diet/nutrition outcomes  *Diet quality*  Estimated by using the Healthy Eating  Index–2010 calculated by Block Dietary Data Systems. For 12 components, intakes  of foods and nutrients are represented on a density basis, counted as amount per 1000 kcal. The 9 adequacy components include total fruit, whole fruit, total vegetables, greens and beans, whole grains, dairy, total protein foods, seafood and plant proteins, and FAs, which reflects the ratio of PUFAs and MUFAs to SFAs. Three moderation components include  refined grains, sodium, and empty calories, which reflect calories from solid fats, alcohol, and added sugars. For adequacy components, a score of zero is assigned for no intake, and the scores increase proportionately as intake increases up to the recommended level. For moderation components, intake at the recommended level is assigned the maximum score and the score decreases as intake increases. The total HEI-2010 score ranges from zero (nonadherence) to 100 (perfect adherence). | Associations between factors and diet/nutrition  Means of HEI-2010 score adjusted for age at study, sex, race/ethnicity, education, smoking status, alcohol  consumption, physical activity, and weight status by using ANCOVA; for cancer and treatment characteristics, means, SEs, and P values were adjusted for age at study, sex,  and cancer diagnosis by using ANCOVA.  Adjusted means of HEI-2010 score:  *Age at study, p < 0.0001*   - 18–29 years: 55.1, 95% CI (54.0, 56.2) - 30–39 years: 56.3, 95% CI (55.2, 57.4) - 40–64 years: 58.0, 95% CI (56.7, 59.3)   *Sex, p < 0.0001*   - Male: 53.6, 95% CI (52.6, 54.7) - Female: 59.3, 95% CI (58.3, 60.4)   *Race/ethnicity, p = .06*   - Non-Hispanic white: 57.1, 95% CI (56.2, 57.9) - Other: 55.9, 95% CI (54.5, 57.2)   *Education, p < 0.0001*   - Grades 0–12: 53.2, 95% CI (52.0, 54.4) - Some post–high school: 55.7, 95% CI (54.6, 56.8) - College graduate: 60.1, 95% CI (59.4, 61.7)   *Smoking status, p < 0.0001*   - Nonsmoker: 57.9, 95% CI (57.0, 58.9) - Former smoker: 57.7, 95% CI (56.1, 59.2) - Current smoker: 53.9, 95% CI (52.7, 55.1)   *Alcohol consumption, p = .07*   - Nondrinker: 56.0, 95% CI (54.9, 57.1) - <14 g ethanol/d: 57.1, 95% CI (56.1, 58.2) - ≥14 g ethanol/d: 56.3, 95% CI (55.9, 57.7)   *Physical activity, p < 0.0001*   - Active: 58.5, 95% CI (57.5, 59.6) - Inactive: 54.5, 95% CI (53.4, 55.5)   *Weight status, p = .006*   - Underweight: 54.1, 95% CI (51.6, 56.7) - Normal weight: 57.3, 95% CI (56.3, 58.3) - Overweight: 58.0, 95% CI (57.0, 59.0) - Obese: 56.5, 95% CI (55.5, 57.5)   *Height, SDS, p = .38*   - <2: 57.1, 95% CI (55.4, 58.8) - ≥2: 57.9, 95% CI (57.4, 58.4)   *Primary diagnosis, p = .01*   - Leukaemia: 58.7, 95% CI (57.9, 59.5) - Lymphoma: 59.4, 95% CI (58.3, 60.4) - Embryonal tumour: 56.9, 95% CI (55.5, 58.2) - Sarcoma: 57.3, 95% CI (56.0, 58.6) - Central nervous system tumour: 57.7, 95% CI (56.1, 59.3) - Other: 57.0, 95% CI (55.2, 58.8)   *Age at diagnosis, p = .045*   - <5: 56.9, 95% CI (56.0, 57.8) - 5–9: 58.2, 95% CI (57.1, 59.2) - 10–14: 58.5, 95% CI (57.4, 59.5) - ≥15: 58.2, 95% CI (56.9, 59.5)   *Any radiation, p = .83*   - No: 57.7, 95% CI (56.9, 58.6) - Yes: 57.8, 95% CI (57.2, 58.5)   *Brain radiation dose, p = .95*   - 0 Gy: 58.2, 95% CI (57.1, 59.3) - 1–19.9 Gy: 57.6, 95% CI (55.5, 59.7) - 20–29.9 Gy: 57.7, 95% CI (55.9, 59.6) - ≥30 Gy: 58.1, 95% CI (55.9, 60.5)   *Abdomen radiation dose, p = .02*   - 0 Gy: 58.9, 95% CI (58.0, 59.7) - 1–19.9 Gy: 57.2, 95% CI (55.0, 59.4) - 20–29.9 Gy: 56.7, 95% CI (54.8, 58.5) - ≥30 Gy: 56.1, 95% CI (54.2, 58.0)   *Cumulative alkylating agent dose, p = .85*   - 0 mg/m²: 58.0, 95% CI (57.2, 58.9) - 1–7999 mg/m²: 57.6, 95% CI (56.5, 58.6) - 8000–11,999 mg/m²: 57.5, 95% CI (56.2, 58.8) - ≥12,000 mg/m²: 57.7, 95% CI (56.5, 58.9)   *Cumulative anthracycline dose, p = .43*   - 0 mg/m²: 58.1, 95% CI (57.4, 58.9) - 1–99 mg/m²: 57.0, 95% CI (55.5, 58.5) - 100–299 mg/m²: 57.6, 95% CI (56.5, 58.7) - ≥300 mg/m²: 57.0, 95% CI (55.5, 58.5)   *Cumulative glucocorticoid dose, p = .03*   - 0 mg/m²: 57.7, 95% CI (56.9, 58.4) - 1–1499 mg/m²: 57.1, 95% CI (55.4, 58.7) - 1500–8999 mg/m²: 59.7, 95% CI (57.9, 61.5) - ≥9000 mg/m²: 56.9, 95% CI (55.0, 58.5) |

Abbreviations: CI, confidence interval, CNS, central nervous system, Gy, grey, HEI, Healthy Eating Index, SD, standard deviation, SDS, standard deviation score

| ***Bhandari et al.*** Prevalence and risk factors for vitamin D deficiency in long‐term childhood cancer survivors, Pediat Blood Cancer, 2021, 68(7), e29048. | | | |
| --- | --- | --- | --- |
| **Study design**  **Treatment era**  **Years of follow-up** | **Participants** | **Variable definitions** | **Results** |
| Study design  Cross-sectional study  Treatment era  Not reported  Follow-up duration  Median follow-up from completion of therapy: 14.2  years (range 2–65 years) | Type and number of participants  N=446 childhood cancer survivors  Cancer diagnosis   - Leukaemia/lymphoma: N=313 (70.2%) - Solid tumour: N=111 (24.9%) - Non-malignant hematologic disease: N=22 (4.9%)   Age at diagnosis  Not reported.  Age at follow-up  Mean age at follow-up for study: 27.5 years (SD 11.4)  Cancer treatment  *Hematopoietic cell transplantation*  *status*   - No hematopoietic cell transplantation: N=264 (59.2%) - Autologous: N=44 (9.9%) - Allogeneic: N=138 (30.9%)   *Prednisone cumulative dose (if*  *available; dose information incomplete in 86 patients)*   - None: N=153 (42.5%) - Low (1–4275 mg/m2): N=104 (28.9%) - High (>4275 mg/m2): N=103 (28.6%)   *Methotrexate cumulative dose (if*  *Available; dose information incomplete in 99 patients)*   - None: N=207 (59.7%) - Low (1–3690 mg/m2): N=70 (20.2%) - High (>3690 mg/m2): N=70 (20.2%)   Controls/normal population (if applicable)  Not applicable  Additional participant characteristics (if applicable):  Not applicable | Diet/nutrition outcomes  *Vitamin D level*  Total 25-hydroxy vitamin D level (ng/ml), measured via immunoassay was recorded for all patients. Vitamin D levels below the lower limit of what is detectable were recorded as “0.” Vitamin D levels ≤20 ng/mL, 21–29 ng/mL, and ≥30 ng/mL were considered deficient, insufficient, and sufficient, respectively.  Other variables  *Body Mass Index*  BMI was derived from height and weight. BMI percentile was calculated using age/sex-adjusted population norms for individuals between 2 and 20 years of age and categorized as normal/underweight (<85th percentile), overweight (between  85th and 95th percentile), or obese (≥95th percentile). BMI for individuals ≥20 years of age was also categorized as normal/underweight (<25), overweight (between 25 and 30), or obese (≥30). | Associations between factors and diet/nutrition  Vitamin D deficiency  *Race/ethnicity (Non-Hispanic White = ref.)*   - Hispanic/Black: OR = 2.40, 95% CI (1.41–4.09), p <0.01 - Other: OR = 1.27, 95% CI (0.61–2.66), p = 0.53   *BMI category (normal/underweight = ref.)*   - Overweight: OR = 1.78, 95% CI (1.03–3.07), p = 0.04 - Obese: OR = 2.40, 95% CI (1.39–4.13), p <0.01   Multivariable models adjusted for sex, race/ethnicity, and HCT status. |

Abbreviations: CI, confidence interval, HCT, hematopoietic cell transplantation, SD, standard deviation, OR, odds ratio

| ***Emmons et al.*** Smoking Among Participants in the Childhood Cancer Survivors Cohort: The Partnership for Health Study, J Clin Oncol, 2003, 21(2), 189–196. | | | |
| --- | --- | --- | --- |
| **Study design**  **Treatment era**  **Years of follow-up** | **Participants** | **Variable definitions** | **Results** |
| Study design  Baseline data collection of a randomized trial  Treatment era  Not reported  Follow-up duration  Time since diagnosis:   - 0-16 years: N=162 (20%) - 17-20 years: N=216 (27%) - 21-24 years: N=230 (29%) - 25+ years: N= 185 (23%) - Missing: N=3 (0.4%)   Mean time since treatment = 21 years (SD 4.5). | Type and number of participants  N=796 childhood cancer survivors  Cancer diagnosis   - Leukaemia: N=205 (26%) - Hodgkin’s disease: N=144 (18%) - CNS malignancy: N=93 (12%) - Non-Hodgkin’s lymphoma: N=90 (11%) - Bone cancer: N=84 (11%) - Soft tissue sarcoma: N=75 (9%) - Kidney cancer: N=54 (7%) - Neuroblastoma: N=48 (6%)   Age at diagnosis   - 0-3 years: N=152 (19%) - 4-9 years: N=233 (29%) - 10-14 years: N=223 (28%) - 15-21 years: N=185 (23%) - Missing: N=3 (0.4%)   Mean age at diagnosis = 9.6 years (SD 5.8).  Age at follow-up   - 18-25 years: N=185 (23%) - 26-30 years: N=223 (28%) - 31-35: N=184 (23%) - 36-50: N=204 (26%)   Mean age at study enrolment = 31 years (SD 6.66).  Cancer treatment   - Chemotherapy: N=24 (3%) - Radiation: N=3 (<1%) - Surgery: N=65 (8%) - Radiation and chemotherapy: N= 59 (7%) - Chemotherapy and surgery: N= 120 (15%) - Radiation and surgery: N= 113 (14%) - Radiation, chemotherapy, and surgery: N=259 (33%) - Missing: N=153 (19%)   Controls/normal population (if applicable)  Not reported  Additional participant characteristics (if applicable):  Not applicable | Smoking measures  *Smoking rate*  Assessed using self-reported number of cigarettes smoked per day. Seven of the 796 participants were not daily smokers, so the authors calculated number of cigarettes smoked per week for all participants as the measure of smoking rate.  *Self-reported number of quit attempts*  Frequency of quit attempts in the past 12 months.  *Nicotine dependence*  Determined by assessing time to first cigarette. Smoking within the first 30 minutes after waking was considered as nicotine dependent.  *Readiness to quit smoking*  Assessed using the stages of change algorithm. Participants were placed into one of three stages of readiness for change: (1) precontemplators, not thinking about quitting in the next 6 months; (2) contemplators, thinking about quitting in the next 6 months; or (3) in preparation, thinking of quitting in the next month and having made recent attempts to quit. Because all the participants in this study were active smokers, no one was placed in the action stage.  Other variables  *Perceived vulnerability specific to cancer and smoking*  Measured using Tyc’s Perceived Importance of Health Protection scale, developed for adolescent cancer survivors. No other information reported. | Associations between factors and smoking  Number of cigarettes smoked per week  All factors below (except high school graduate) were associated with higher smoking rates in the multivariate model. All variables were included in the model. β represents the increase in the odds of higher smoking rates.  *Age:* β = 0.0226, p < .0001  *Education (more than high school = ref.)*   - Less than high school: β = 0.3311, p =.0019 - High school grad: β = 0.1295, p = .0824   *Social norms (none/few smoke = ref.)*   - Most smoke: β = 0.5657 p < .0001 - About half smoke: β = 0.1881 p = .016   Support for quitting (not at all = ref.)   - A little or a lot: β = 0.2027, p = .0256   *Global severity index*: β = 0.0077, p = .0238  Quit attempts  *Age (no ref.)*  OR = 0.97, 95% CI (0.95 - 0.99), p = 0.005  *Support for quitting (not at all = ref.)*   - A lot: OR = 1.66, 95% CI (1.12 - 2.47), p = 0.012 - A little: OR = 1.43, 95% CI (0.95 - 2.14), p = 0.087   *Social support (no ref.)*  OR = 0.85, 95% CI (0.73 - 0.99), p = 0.038  *Perceived vulnerability (no ref.)*  OR = 1.17, 95% CI (1.09 - 1.25), p < 0.0001  *Social norms (none/few/half smoke = ref.)*  Most smoke: OR = 0.62, 95% CI (0.45 - 0.86), p = 0.004  Nicotine dependence  *Education (college grad = ref.)*   - ≤ High school: OR = 2.81, 95% CI (1.57 - 5.04), p = 0.0005 - High school grad: OR = 2.39, 95% CI (1.48 - 3.85), p = 0.0004 - Post high school: OR = 1.90, 95% CI (1.19 - 3.01), p = 0.0068   *Social norms (none/few smoke = ref.)*   - Most smoke: OR = 2.16, 95% CI (1.46 - 3.18), p = 0.0001 - About half smoke: OR = 1.72, 95% CI (1.23 - 2.41), p = 0.0015   *Age*  OR = 1.04, 95% CI (1.01 - 1.06), p = 0.0020  *GSI (not severe = ref.)*  Severe: OR = 1.74, 95% CI (1.13 - 2.70), p = 0.0123  Readiness to quit  *Support for quitting (not at all = ref.)*   - A lot: OR = 3.78, 95% CI (2.53-5.64), p < .0001 - A little: OR = 2.19, 95% CI (1.46 - 3.27), p = .0001   *Perceived vulnerability (no ref.)*  OR = 1.20, 95% CI (1.13 – 1.29), p < .0001 |

Abbreviations: CI, confidence interval, CNS, central nervous system, GSI, global severity index, OR, odds ratio, SD, standard deviation

| ***Cox et al.*** Promoting physical activity in childhood cancer survivors, Cancer, 2009, 115(3), 642–654. | | | |
| --- | --- | --- | --- |
| **Study design**  **Treatment era**  **Years of follow-up** | **Participants** | **Variable definitions** | **Results** |
| Study design  Structural equation modelling data were derived from the Childhood Cancer Survivors Study of adult survivors.  Treatment era  Diagnosis 1970-1986  Follow-up duration  Mean years since diagnosis: 23.74 | Type and number of participants  N=838 childhood cancer survivors  Cancer diagnosis   - Leukaemia, Hodgkin disease, lymphoma: N=593 (66.7%) - Solid tumour: N=205 (23.1%) - Bone cancer: N=91 (10.2%)   Age at diagnosis  Mean age at diagnosis: 9.25 years (SD 5.87)  Age at follow-up  Mean age: 30.98 years (SD 7.50)  Cancer treatment  Not reported  Controls/normal population (if applicable)  Not applicable  Additional participant characteristics (if applicable):  Not applicable | Physical activity measures  *Physical activity*  Physical activity participation was measured as a binary outcome derived from the Behavioural Risk Factor Surveillance Study Survivors were asked (yes=1; no=2), ‘‘During the past month, did you participate in any physical activities or exercises, such as running, calisthenics, golf, bicycling, swimming, wheelchair basketball, or walking, for exercise?’’  Other variables  *Stamina*  Derived from 5 observed items on the Physical Function measure of the 36-item Short Form Health Survey, survivors were asked about the extent to which their physical health limited their ability to climb several flights of stairs, climb 1 flight of stairs, walk more than 1 mile, walk several blocks, and walk 1 block (3=not limited at all, 2=limited a little, 1=limited a lot).  *Fear*  Three observed variables measured on a 5-point Likert scale assessed the extent of survivors’ fear (1=not at all concerned, 5=extremely concerned) about their future  health, the return of their cancer, and the discovery of a health problem during a routine check-up.  *Affect*  Derived from 3 observed measures and rated on a 6-point Likert scale (1=all of the time, 6=none of the time), survivors assessed the frequency of feeling unhappy, downhearted and blue, and not cheerful.  *Survivor-provider interaction*  Four items asked survivors to rate on a 5-point Likert scale (1=not at all, 5=extremely) the extent to which they believed that their physician took enough time to answer their questions, they could ask their physician questions about cancer, their fears and concerns had been addressed by their physician, and their PCP could handle cancer-related problems.  *Covariates*  Eleven directly observed, independent variables were associated significantly with recent physical activity as covariates in the final analytical models. All were self-reported by survivors:   1. their PCP’s familiarity with cancer-related problems, (1=familiar, 2=not familiar) 2. current pain resulting from cancer or its treatment (1=no pain, 5=excruciating pain) 3. frequency of fatigue (1=all the time, 6=none of the time) 4. whether survivors had discussed the risk of recurrent cancer with their PCP (1=yes, 2=no) 5. baseline frequency of aerobic exercise (defined as sufficient to induce sweating or breathing hard, lasting 20 minutes 0 days per week or 7 days per week) 6. 6) age at diagnosis 7. current anxiety as a result of cancer or its treatment (1=no anxiety, 5=extreme anxiety) 8. current highest school grade completed 9. whether the survivor had seen a primary care physician since cancer treatment ended (1=yes, 2=no) 10. modified from the Multidimensional Health Locus of Control Scales for intrinsic motivation, survivors rated 4 items (e.g., ‘‘I am in control of my health.’’ ‘‘The main thing that affects my health is what I myself do’’) on a 6-point Likert scale (1=strongly disagree, 6=strongly agree 11. extrinsic motivation; survivors rated 4 items (e.g., ‘‘Health professionals control my health’’; ‘‘Regarding my health, I can only do what my physician tells me to do’’) on a 6-point Likert scale (1=strongly disagree, 6=strongly agree). | Associations between factors and physical activity  Results obtained with Structural Equation Modeling including physical activity (reported here), stamina, fear, affect, fatigue, patient-provider interaction, intrinsic motivation, and cancer anxiety.  The estimates below quantify the magnitude and direction of the relationship between the predictor variable and the outcome. The standard YX is an approximation of the strength of the relative contribution of the background variable to the outcome (either the latent construct or the path outcome) obtained by using data that adjust for the differences in measurement scales.  Men  *Physical activity*   - Education: Estimate = -0.312, SE = 0.101, Estimate/SE = -3.102, Standard YX = -0.304 - Baseline exercise frequency: Estimate = -0.227, SE = 0.053, Estimate/SE = -4.327, Standard YX = -0.413 - Provider knowledge: Estimate = 0.403, SE = 0.134, Estimate/SE = 3.006, Standard YX = 0.280 - Fear: Estimate = 0.417, SE = 0.134, Estimate/SE = 3.110, Standard YX = 0.282   Women  *Physical activity*   - Fatigue: Estimate = -0.270, SE = 0.079, Estimate/SE = 3.438, Standard YX = -0.275 - Stamina: Estimate = -0.682, SE = 0.154, Estimate/SE = 4.433, Standard YX = -0.321 - Baseline exercise frequency: Estimate = 0.122, SE = 0.038, Estimate/SE = -3.220, Standard YX = -0.211 |

Abbreviations: PCP, primary-care physician, SD, standard deviation, SE = standard error

| ***Klosky et al.***  Risky health behaviour among adolescents in the childhood cancer survivor study cohort, J Pediatr Psychol, 2012, 37(6):634-46. | | | |
| --- | --- | --- | --- |
| **Study design**  **Treatment era**  **Years of follow-up** | **Participants** | **Variable definitions** | **Results** |
| Study design  Retrospective cohort study  Treatment era  Diagnosis between 1970-1986.  Follow-up duration  Not reported. | Type and number of participants  N = 307 childhood cancer survivors  Cancer diagnosis   - CNS: N=40 (13.03%) - Leukaemia: N= 95 (30.94%) - Non-Hodgkin’s Lymphoma: N=4 (1.30%) - Wilms tumour: N=56 (18.24%) - Neuroblastoma: N=90 (29.32%) - Soft tissue sarcoma: N=19 (6.19%) - Bone cancer: N= 3 (0.98%)   Age at diagnosis  Time since diagnosis (years):   - <15: N=6 (1.95%) - 15–20: N=300 (97.72%) - >20: N=1 (0.33%)   Mean age = 18.1 years (SD 1.0, range 15.4-20.4).  Age at follow-up   - 14-17 years: N=129 (42.02%) - 18-20 years: N=178 (57.98%)   Cancer treatment   - Surgery only: N=41 (13.35) - Radiation therapy (no chemo): N=11 (3.58%) - Chemotherapy + radiation: N=99 (32.25%) - Chemotherapy (no radiation): N=144 (46.91%) - No chemo, surgery, or radiation: N=2 (0.65%) - Unknown: N=10 (3.26%)   *CNS treatment*   - No CNS treatment: N=193 (62.87%) - Any CNS treatment: N=114 (37.13%)   Controls/normal population (if applicable)  N=97 siblings.   - 14-17 years: N=58 (59.79%) - 18-20 years: N=39 (40.21%)   Mean age = 17.5 years (SD 1.4, range 14.6-20.1).  Additional participant characteristics (if applicable):  Not applicable | Smoking measures  *Smoking status*  Self-reported smoking status were obtained by the self-administered questionnaire Child Health and Illness Profile—Adolescent Edition. Questions were assessed on a Likert-type scale from 1 to 5. Response options were then collapsed into three mutually exclusive categories with scores of 1 (past week) and 2 (past month) forming the ‘‘current use’’ category (i.e., use within the past month), scores of 3 (past year) and 4 (more than a year ago) forming the ‘‘past use’’ category (i.e., previous, but not current use), and a score of 5 (never) forming the ‘‘never used’’ category.  Alcohol consumption measures  *Alcohol consumption and risky drinking*  Self-reported alcohol consumption was obtained by the self-administered questionnaire Child Health and Illness Profile—Adolescent Edition. Questions were assessed on a Likert-type scale from 1 to 5. Response options were then collapsed into three mutually exclusive categories with scores of 1 (past week) and 2 (past month) forming the ‘‘current use’’ category (i.e., use within the past month), scores of 3 (past year) and 4 (more than a year ago) forming the ‘‘past use’’ category (i.e., previous, but not current use), and a score of 5 (never) forming the ‘‘never used’’ category. Risky drinking was defined as more than 4 drinks in 1 day or 14 drinks in 1 week for males and 3 drinks in 1 day or 7 drinks in 1 week for women.  Other variables  *Participant mental health*  Measured via the Psychosocial Disorder subdomain score of the CHIP—AE. This subdomain queries emotional, mental or behavioral problems which may occur in adolescence. Respondents indicated whether they had (a) never had the problem, (b) previously had a problem but not within the past year, or (c) experienced the problem within the past year. | Associations between factors and smoking, alcohol consumption, and drug use  All results are adjusted for age and gender.  *Cigarette use*   - Survivor vs. Sibling / Past vs. Never: OR = 0.59, 95% CI (0.30, 1.14), p = 0.12 - Survivor vs. Sibling / Current vs. Never: OR = 0.73, 95% CI (0.38, 1.41), p = 0.35   *Smokeless tobacco*   - Survivor vs. Sibling / Past vs. Never: OR = 0.32, 95% CI (0.11, 0.92), p = 0.03 - Survivor vs. Sibling / Current vs. Never: OR = 1.06, 95% CI (0.21, 5.42), p = 0.95   *Beer/Wine consumption*   - Survivor vs. Sibling / Past vs. Never: OR = 1.27, 95% CI (0.67, 2.42), p = 0.47 - Survivor vs. Sibling / Current vs. Never: OR = 0.50, 95% CI (0.29, 0.86), p = 0.01   *Hard liquor/Mixed drinks consumption*   - Survivor vs. Sibling / Past vs. Never: OR = 0.61, 95% CI (0.34, 1.10), p = 0.10 - Survivor vs. Sibling / Current vs. Never: OR = 0.56, 95% CI (0.31, 1.00), p = 0.05   *Binge drinking*   - Survivor vs. Sibling / Past vs. Never: OR = 0.89, 95% CI (0.45, 1.76), p = 0.74 - Survivor vs. Sibling / Current vs. Never: OR = 0.53, 95% CI (0.29, 0.97), p = 0.04   Demographic, Diagnostic, and Treatment Differences Among  Adolescent Survivors of Childhood Cancer With a Poor Overall  Behavioural Health Outcome  *Gender (male = ref).*  Female: OR = 0.67, 95% CI (0.25–1.79), p = 0.43  *Race (White = ref.)*  Non-White: OR = 2.07, 95% CI (0.55–7.83) 0.29  *Age (15-17 years = ref.)*  18–20 years: OR = 1.10, 95% CI (0.36–3.37), p = 0.87  Age at diagnosis (0-1 years = ref.)  2–3 years: OR = 1.12, 95% CI (0.34–3.71), p = 0.85  *Diagnosis (leukaemia = ref.)*   - Neuroblastoma: OR = 0.24, 95% CI (0.04–1.50), p = 0.13 - Other: OR = 0.36, 95% CI (0.08–1.58), p = 0.18   *CNS treatment (no = ref.)*  Any CNS treatment: OR = 0.30, 95% CI (0.07–1.26), p = 0.10  *Household income (<60,000/year=ref.)*  60,000þ/year: OR = 0.56, 95% CI (0.19–1.68), p = 0.30  *Mental health score*  OR = 0.24, 95% CI (0.13–0.43), p = 0.0001 |

Abbreviations: CI, confidence interval, CHIP-AE, Child Health and Illness Profile—Adolescent Edition, CNS, central nervous system, OR, odds ratio

| ***Emmons et al.*** Peer-delivered smoking counselling for childhood cancer survivors increases rate of cessation: The Partnership for Health Study. J Clin Oncol, 2005, 23: 6515-6523. | | | |
| --- | --- | --- | --- |
| **Study design**  **Treatment era**  **Years of follow-up** | **Participants** | **Variable definitions** | **Results** |
| Study design  Randomized controlled  trial  Treatment era  Diagnosis between 1970-1986  Follow-up duration  Participants were at least 5 years from the time of diagnosis. No other information (such as starting point) reported. | Type and number of participants  N=796 childhood cancer survivors  Cancer diagnosis:   - Leukaemia: N=205 (26%) - Hodgkin’s disease: N=144 (18%) - CNS malignancy: N=93 (12%) - Non-Hodgkin’s lymphoma: N=90 (11%) - Bone cancer: N=84 (11%) - Soft tissue sarcoma: N=75 (9%) - Kidney cancer: N=54 (7%) - Neuroblastoma: N=48 (6%)   Age at diagnosis:   - 0-3 years: N=151 (19%) - 4-9 years: N=231 (29%) - 10-14 years: N=223 (28%) - ≥ 15 years: N=191 (24%)   Age at follow-up:  Mean age 31 years, SD 6.66 years  Cancer treatment:   - Chemotherapy and surgery: N=120 (15%) - Radiation and chemotherapy: N=59 (7%) - Radiation and surgery: N=113 (14%) - Radiation, chemotherapy, and surgery: N=259 (33%) - Radiation, chemotherapy, or surgery only: N=92 (12%) - Data missing: N=153 (19%)   Controls/normal population (if applicable)  N=398 participants were allocated to the intervention group and N=398 to the control group.  Additional participant characteristics (if applicable):  Not applicable | Smoking measures  *Smoking status*  7-day point-prevalence smoking status, number of recent quit attempts, smoking rate, nicotine dependence (time from waking to first cigarette), and NRT use were assessed.  Other variables  *Self-efficacy*  Defined by measures of confidence in one’s ability to quit smoking using a 5-point Likert response scale. | Associations between factors and smoking,  Predictors of smoking cessation through  logistic regression adjusted for age and sex:  *Age group (vs. ≥ 36 years), p = NS*   - 18-25 years: OR 0.52, 95% CI (0.28, 0.97) - 26-30 years: OR 0.59, 95% CI (0.33, 1.03) - 31-35 years: OR 0.69, 95% CI (0.38, 1.25)   *Sex (female vs. male), p = NS*  OR 1.13, 95% CI (0.73, 1.76)  *Long-term self-efficacy (yes vs. no), p < .05*  OR 1.36, 95% CI (1.13, 1.63)  *Intervention (yes vs. no), p < .05*  OR 1.69, 95% CI (1.09, 2.63)  *Mean positive feelings (yes vs. no), p = NS*  OR 0.58, 95% CI (0.31, 1.08)  *Have a lot of energy past 4 weeks (baseline yes vs. no), p < .05*  OR 1.45, 95% CI (1.05, 1.99)  *Depressive symptoms (yes vs. no), p = NS*  OR 0.69, 95% CI (0.48, 1.01) |

Abbreviations: CI, confidence interval, NRT, nicotine replacement therapy, OR, odds ratio, SD, standard deviation

| ***Kahalley et al.***  Risk Factors for Smoking among Adolescent Survivors of Childhood Cancer: A Report From the Childhood Cancer Survivor Study, Pediatr Blood Cancer, 2012; 58:428–434 | | | |
| --- | --- | --- | --- |
| **Study design**  **Treatment era**  **Years of follow-up** | **Participants** | **Variable definitions** | **Results** |
| Study design  Retrospective cohort study  Treatment era  Diagnosis 1970-1986.  Follow-up duration  Not reported. | Type and number of participants  N = 307 childhood cancer survivors  Cancer diagnosis   - CNS: N=40 (11.11%) - Leukaemia: N=95 (26.39%) - Solid tumours: N=168 (46.67%) - Lymphomas: N=4 (1.11%)   Age at diagnosis  Mean age at diagnosis = 1.1 years, (SD 1.0, range 0–3)  Age at follow-up  Mean age at survey = 17.6 years, (SD 1.1, range 15–20)  Cancer treatment   - No radiation or chemotherapy: N=43 (14.00%) - Chemotherapy without radiation: N=144 (46.88%) - Radiation: N=110 (35.92%) - History of CRT: N=62 (20.20%) - Missing: N=20 (6.51%)   Controls/normal population (if applicable)  N=97 siblings (no other information reported)  Additional participant characteristics (if applicable):  Not applicable | Smoking measures  *Smoking*  Participants rated the last time they smoked a cigarette on a five-point scale (never, more than a year ago, in the past year, in the past month, in the past week). Participants were categorized as ‘‘Never Smokers’’ if they responded that they never smoked a cigarette, while all others were categorized as ‘‘Ever Smokers.’’ Participants who endorsed smoking in the past week were categorized as ‘‘Recent Smokers,’’ while all others were categorized as ‘‘Non-Smokers.’’  *Peer smoking*  Participants reported on how many of their friends smoke cigarettes (None, Some, Most, All). A dichotomous peer smoking variable was derived due to the restricted variability across responses in this sample. Due to the low frequency of the None response on this item (i.e., only four ever smokers and one recent smoker reported having no smoking friends), responses of none or some friends who smoke were categorized as having ‘‘few’’ smoking friends while responses of Most or All friends who smoke were categorized as ‘‘many’’ smokers in the peer  network.  *Household smoking*  Participants reported whether anyone else in the household smokes cigarettes (yes/no).  Other variables  *Binge eating*  Participants reported the number of times they engaged in binge eating over the past 12 months (ever/never). | Associations between factors and smoking  Multivariate Poisson regression model of recent smoking among survivors adjusted for all variables reported:  *Age*  RR = 1.12, 95% CI (0.86-1.45), p = .408  Interpreted as an increase of 12% in the risk of recent smoking for each one-unit increase in age.  *Gender (female = ref.)*  Male: RR = 1.65, 95% CI (0.91-3.00), p = .097  *History of CRT (yes = ref.)*  No: RR = 2.40, 95% CI (1.12-5.17), p = .025  *Suicidal behaviour (no = ref.)*  Yes: RR = 1.89, 95% CI (1.00-3.56), p = .049  *Household smoking (no = ref.)*  Yes: RR = 2.24, 95% CI (1.21-4.16), p = .011  *Peer smoking X binge eating:*  *Peer smoking: many smoking friends (no binge eating = ref.)*   - Binge eating: RR = 0.79, 95% CI (0.34-1.85), p = .593   *Peer smoking: few smoking friends (no binge eating = ref.)*   - Binge eating: RR = 3.37, 95% CI (1.17-9.71), p = .024   Multivariate Poisson regression model of ever smoking among survivors adjusted for all variables reported:  *Age*  RR = 1.11, 95% CI (0.95-1.31), p = 0.189  Interpreted as an increase of 11% in the risk of recent smoking for each one-unit increase in age.  *Peer smoking X gender*  *Peer smoking: many smoking friends (female = ref.)*   - Male: RR = 1.48, 95% CI (0.99-2.21), p = 0.057   *Peer smoking: few smoking friends (female = ref.)*   - Male: RR = 0.71, 95% CI (0.43-1.18), p = 0.187   *Peer smoking X history of CRT:*  *Peer smoking: many smoking friends (CRT = ref.)*   - No CRT: RR = 0.98, 95% CI (0.65-1.49), p = 0.930   *Peer smoking: few smoking friends (CRT = ref.)*   - No CRT: RR = 4.47, 95% CI (1.43-13.9), p = 0.010 |

Abbreviations: CI, confidence interval, CNS, central nervous system, CRT, cranial radiotherapy, SD, standard deviation, RR, relative risk

| ***Bougas et al.*** Smoking and cannabis use among childhood cancer survivors: Results of the French childhood cancer survivor study. Cancer Epidemiol Biomarkers Prev, 2021, 30(10):1965-1973. | | | |
| --- | --- | --- | --- |
| **Study design**  **Treatment era**  **Years of follow-up** | **Participants** | **Variable definitions** | **Results** |
| Study design  Cohort study  Treatment era  Diagnosis between 1945-2000. Decade of diagnosis of first cancer:   - < 1975: N = 670 (20.3%) - 1975 - 1984: N = 1,293 (39.3%) - 1985 - 1994: N = 880 (26.7%) - ≥ 1995: N = 450 (13.7%)   Follow-up duration  Not reported | Type and number of participants  N=3293 childhood cancer survivors  Cancer diagnosis   - Wilms Tumour: N = 607 (18.4%) - Neuroblastoma: N = 470 (14.3%) - Hodgkin's lymphoma: N = 213 (6.5%) - Non-Hodgkin's lymphoma: N = 388 (11.8%) - Soft tissue sarcoma: N = 376 (11.4%) - Bone sarcoma: N = 315 (9.6%) - CNS tumour: N = 377 (11.4%) - Retinoblastoma: N = 148 (4.5%) - Other solid cancers (gonadal tumour, thyroid tumour and other types of carcinoma): N = 399 (12.1%)   Age at diagnosis   - < 5 years: N = 1,580 (48.0%) - 5-9 years: N = 731 (22.2%) - 10-14 years: N = 710 (21.6%) - ≥ 15 years: N = 272 (8.3%)   Age at follow-up  Age at the questionnaire:   - < 25 years: N = 516 (15.7%) - 25-29 years: N = 669 (20.3%) - 30-39 years: N = 1,356 (41.2%) - ≥ 40 years: N = 752 (22.8%)   Cancer treatment   - Chemotherapy: N = 2,589 (78.6%) - Thoracic radiation therapy: N=508 (15.4%)   No other information reported.  Controls/normal population (if applicable)  N=2887 sex-, age-, education level- and marital status-matched controls from the general population  Additional participant characteristics (if applicable):  Not applicable | Smoking measures  *Smoking status*   - Ever smoking: defined as a binary variable (yes, no) using the question “Have you ever smoked cigarettes regularly?” - Current smoking: defined as a binary variable (yes, no) using the question “Do you currently smoke cigarettes regularly?”   Subjects who had ever smoked cigarettes regularly but who did not currently smoke were considered as those who quit smoking.  *Number of cigarettes per day*  Current smokers were asked how many cigarettes they smoked per day.  Other variables  Not applicable. | Associations between factors and smoking and drug use  Models adjusted for sex, age, educational level, and marital status. None of the results were found to be significant unless indicated.  *Current smokers (survivors vs. controls)*   - FCCSS overall: RR = 0.64, 95% CI (0.59-0.68) - Wilms Tumour: RR = 0.70, 95% CI (0.60-0.80) - Neuroblastoma: RR = 0.68, 95% CI (0.58-0.80) - Hodgkin's lymphoma: RR = 0.55, 95% CI (0.42-0.73) - Non-Hodgkin's lymphoma: RR = 0.70, 95% CI (0.59-0.83) - Soft tissue sarcoma: RR = 0.76, 95% CI (0.64-0.90) - Bone sarcoma: RR = 0.66, 95% CI (0.54-0.81) - CNS tumour: RR = 0.31, 95% CI (0.24-0.42) - Retinoblastoma: RR = 0.51, 95% CI (0.37-0.70) - Other solid cancers: RR = 0.69, 95% CI (0.58-0.82)   Current smoking (N=2887)  *Childhood cancer type (vs. Wilms tumour)*   - Neuroblastoma: RR = 1.00, 95% CI = 0.81-1.22 - Hodgkin's lymphoma: RR = 1.03, 95% CI (0.72-1.45) - Non-Hodgkin's lymphoma: RR = 1.02, 95% CI (0.81-1.28) - Soft tissue sarcoma: RR = 1.13, 95% CI (0.91-1.40) - Bone sarcoma: RR = 1.08, 95% CI (0.82-1.42) - CNS tumour: RR = 0.43, 95% CI (0.31-0.59), p < 0.05 - Retinoblastoma: RR = 0.73, 95% CI (0.51-1.04) - Other solid cancers: RR = 0.96, 95% CI (0.75-1.23)   *Age in years at first cancer (< 5 = ref.)*   - 5-9: RR = 0.88, 95% CI (0.73-1.05) - ≥ 10: RR = 0.96, 95% CI (0.79-1.17)   *Chemotherapy (no = ref.)*  Yes: RR = 0.85, 95% CI (0.73-0.99), p < 0.05  *Thoracic radiation therapy (no = ref.)*  Yes: RR = 0.80, 95% CI (0.64-0.99), p < 0.05  *Second cancer (no = ref.)*  Yes: RR = 0.65, 95% CI (0.44-0.96), p < 0.05  *Cardiovascular disease (no = ref.)*  Yes: RR = 0.72, 95% CI (0.52-0.99), p < 0.05  *Sex (female = ref.)*  Male: RR = 1.40, 95% CI (1.23-1.59), p < 0.05  *Age in years at the questionnaire (< 30 = ref)*   - 30-39: RR = 0.98, 95% CI (0.82-1.16) - ≥ 40: RR = 0.78, 95% CI (0.62-0.99), p < 0.05   *Educational level (less than high school = ref.)*   - High School graduate: RR = 0.88, 95% CI (0.75-1.03) - College graduate: RR = 0.59, 95% CI (0.50-0.70), p < 0.05   *Unemployed and seeking work (no = ref.)*  Yes: RR = 1.20, 95% CI (0.99-1.45)  *Marital status (divorced or widowed = ref.)*  Single: RR = 0.82, 95% CI (0.69-0.97), p < 0.05  Smoking cessation (N =1424)  *Childhood cancer type (vs. Wilms tumour)*   - Neuroblastoma: RR = 0.90, 95% CI (0.75-1.09) - Hodgkin's lymphoma: RR = 0.91, 95% CI (0.70-1.19) - Non-Hodgkin's lymphoma: RR = 0.85, 95% CI (0.68-1.07) - Soft tissue sarcoma: RR = 0.86, 95% CI (0.71-1.06) - Bone sarcoma: RR = 0.99, 95% CI (0.79-1.24) - CNS tumour: RR = 1.05, 95% CI (0.80-1.37) - Retinoblastoma: RR = 1.25, 95% CI (0.91-1.72) - Other solid cancers: RR = 0.95, 95% CI (0.77-1.18)   *Age in years at first cancer (< 5 = ref.)*   - 5-9: RR = 1.08, 95% CI (0.92-1.27) - ≥ 10: RR = 1.13, 95% CI (0.94-1.35)   *Chemotherapy (no = ref.)*  Yes: RR = 0.98, 95% CI (0.86-1.13)  *Thoracic radiation therapy (no = ref.)*  Yes: RR = 1.09, 95% CI (0.94-1.28)  *Second cancer (no = ref.)*  Yes: RR = 1.27, 95% CI (1.04-1.55), p < 0.05  *Cardiovascular disease (no = ref.)*  Yes: RR = 1.17, 95% CI (0.95-1.43)  *Sex (female = ref.)*  Male: RR = 0.82, 95% CI (0.73-0.92)  *Age in years at the questionnaire (< 30 = ref)*   - 30-39: RR = 1.09, 95% CI (0.92-1.28) - ≥ 40: RR = 1.19, 95% CI (0.97-1.46)   *Educational level (less than high school = ref.)*   - High School graduate: RR = 1.09, 95% CI (0.92-1.29) - College graduate: RR = 1.45, 95% CI (1.24-1.70)   *Unemployed and seeking work (no = ref.)*  Yes: RR = 1.07, 95% CI (0.89-1.28)  *Marital status (divorced or widowed = ref.)*  Single: RR = 1.23, 95% CI (1.09-1.38) |

Abbreviations: CI, confidence interval, CNS, central nervous system, FCCSS, French childhood cancer survivor study, RR, relative risk

| ***Cappelli et al.*** Binge Drinking, Tobacco, and Marijuana Use Among Young Adult Childhood Cancer Survivors: A Longitudinal Study. J. Pediatr. Oncol. 2021; 38(5):285-294. | | | |
| --- | --- | --- | --- |
| **Study design**  **Treatment era**  **Years of follow-up** | **Participants** | **Variable definitions** | **Results** |
| Study design  Longitudinal cohort study  Treatment era  Diagnosis between 2000-2007.  Follow-up duration  Initial survey measures were completed between 2007 and 2009 (Time 1), with follow-up surveys distributed and completed between 2015 and 2018 (Time 2). The mean time between surveys was 5.1 years (SD 0.56). No other information reported. | Type and number of participants  N = 127 young adult cancer survivors  Cancer diagnosis   - Bone cancer: N= 4 (3.2%) - Brain/CNS: N= 23 (18.1%) - Lymphoma: N= 31 (24.4%) - Leukaemia: N= 46 (36.2%) - Other: N= 23 (18.1%)   Age at diagnosis  Mean age = 12.4 years (SD 2.9)  Age at follow-up  Average age at completion Time 1 survey: 19.9 years  Average age at completion Time 2 survey completion: 24.9 years  Cancer treatment  Not reported  Controls/normal population (if applicable)  Not applicable  Additional participant characteristics (if applicable):  Not applicable | Smoking measures  *Current cigarette use*  Measured at Time 1 and Time 2, defined as any reported use in the past 30 days.  Alcohol measures  *Alcohol use*  Measured at Time 1 and Time 2, defined as any reported use in the past 30 days.  *Binge drinking*  Having five or more drinks on the same occasion at least once in the prior 30 days.  Other variables  *Spirituality*  This construct was screened with a single-item question asking “how important is your religion/spirituality to you?” Scores were 0=don’t  know/not important, 1=somewhat, and 2=very important. Higher scores indicated higher levels of spirituality.  *Treatment Intensity*  The intensity of prior cancer treatment was categorized in four levels of intensity: 1=least intensive (surgery only), 2=moderately intensive (chemotherapy or radiation), 3=very intensive (two or more treatment modalities), and 4=most intensive. | Associations between factors and smoking, alcohol consumption, and drug use  *Binge drinking*   - Perceived stress scale score (continuous): OR = 1.13, 95% CI (0.99−1.29), p = NS - Cigarette use Time 1: OR = 3.34, 95% CI (0.75−14.84), p = NS - Spirituality: OR = 1.13, 95% CI (0.99−1.29), p = NS - Binge drinking Time 1 (ordinal): OR = 2.76, 95% CI (1.08−7.04), p = < .05 - Sex (male vs. female): OR = 0.42, 95% CI = (0.18−0.96), p < .05   *Cigarette use*   - Treatment intensity (continuous): OR = 0.32, 95% CI (0.13−0.78), p = <.05 - Cigarette use Time 1 (ordinal): OR = 5.92, 95% CI (1.17−29.87), p <.05 - Marijuana use (ordinal): OR = 4.07, 95% CI (0.91−18.20), p = NS - Sex (male vs. female): OR = 0.44, 95% CI (0.11−1.77), p = NS |

Abbreviations: CI, confidence interval, CNS, central nervous system; NS, non-significant, OR, odds ratio, SD, standard deviation

| ***Darabos et al.*** Association of Demographic and Cancer-Specific Factors on Health Behaviour Recommendations Specific to Cancer Prevention and Control Among Adolescent and Young Adult Survivors of Childhood Cancer, 2021 J Adolesc Young Adult Oncol, 6(10): 619-628. | | | |
| --- | --- | --- | --- |
| **Study design**  **Treatment era**  **Years of follow-up** | **Participants** | **Variable definitions** | **Results** |
| Study design  Cross-sectional study; combined cross-sectional data from 3 studies.  Treatment era  Not specified  Follow-up duration  Study 1 (N=49): within 1 year off treatment  Study 2 (N=227): within 2 years off treatment  Study 3 (N=31): not specified | Type and number of participants  N = 307 adult survivors of childhood cancer  Cancer diagnosis   - Liquid tumour type (leukaemia/lymphoma): N=184 (59.9%) - Solid tumour type: N=97 (31.6%) - Brain tumour type: N=26 (8.5%)   Age at diagnosis  Mean age 20.33 years (SD 3.37, range 15 to 34)  Age at follow-up  Mean years since diagnosis: 9.34 (SD 6.13, range 0 to 24)  Cancer treatment  Not reported  Controls/normal population (if applicable)  Not applicable  Additional participant characteristics (if applicable):  Not applicable | Physical activity measures  *Meeting physical activity recommendations*  Asked with the question “In a typical week, on how many days do you do (1) vigorousintensity and (2) moderate intensity sports, fitness or recreational activities for at least 10–15 minutes?’’. Responses were weighted and summed to create a modified Leisure Index Score into  active, moderately, insufficiently active; meeting recommendations: active, moderately active; not meeting recommendations: insufficiently active.  Smoking measures  *Tobacco use*  Measured by the question “Do you now smoke cigarettes?’’ (every day; some days; not at all).  Alcohol measures  *Binge drinking*  Having ≥4 drinks (females) and ≥5 drinks (males) in one sitting.  Diet measures  *Fruit/vegetable recommendations*  Meeting recommendations: 3–4 servings, 5 or more servings; not meeting recommendations: no servings, 1–2 servings.  Sun exposure measures  *Sun protective habits*  Self-reported questionnaire (Health Behaviours Survey and questions adapted from the Youth Risk Behaviour Surveillance System questionnaire). The outcome was dichotomized into meeting vs. not meeting health behaviour recommendations.  Other variables  Not applicable | Associations between factors and smoking, alcohol consumption, and sun exposure  Variables in each model included sex, age at survey, race, ethnicity, health insurance, age at diagnosis, time since diagnosis, and time since treatment completion, cancer type, history of relapse, and intensity of treatment.  *Not meeting physical activity recommendations*   - Non-Hispanic ethnicity (vs. Hispanic): OR = 0.28, 95% CI (0.12–0.68) - More time since treatment: OR=0.75, 95% CI (0.60–0.93)   *Tobacco use*   - Sex (female vs. male): OR = 0.22, 95% CI (0.06– 0.79) - White ethnicity (vs. African American, Asian, American Indian/Alaskan Native, and Other Race): OR = 11.38, 95% CI (1.24–104.77)   *Binge drinking*   - Higher age at baseline: OR = 1.89, 95% CI (1.05–3.40) - Solid tumour diagnosis (vs. leukaemia/lymphoma/brain tumour): OR = 1.90, 95% CI (1.00–3.61) - Higher intensity of treatment: OR = 0.60, 95% CI (0.38–0.96)   *Not meeting fruit/vegetable intake recommendations*   - Having had a relapse: OR = 0.47, 95% CI (0.21–1.02)   *Unsafe sun protective habits*   - Sex (females vs. males): OR = 0.57, 95% CI (0.34–0.95) - Non-Hispanic White ethnicity (vs. Hispanic): OR = 0.44, 95% CI (0.23–0.83) |

Abbreviations: CI, confidence interval, OR, odds ratio, SD, standard deviation

| ***Cheung et al.*** Health behaviour practices and expectations for a local cancer survivorship programme: a crosssectional study of survivors of childhood cancer in Hong Kong. Hong Kong Med J, 2022 28(1): 33. | | | |
| --- | --- | --- | --- |
| **Study design**  **Treatment era**  **Years of follow-up** | **Participants** | **Variable definitions** | **Results** |
| Study Design  Prospective, observational, cross-sectional study  Treatment Era  Not reported  Follow-up Duration  Mean (SD) time since treatment completion for all included survivors: 13.4 (7.6) years | Type and number of participants  200 childhood cancer survivors.  For participants ≤16 years or cognitively impaired, a parent was required to be present.  Cancer diagnosis   - Leukaemia: N=78 (39.0%) - Lymphoma: N=28 (14.0%) - Central nervous system tumour: N=14 (7.0%) - Neuroblastoma: N=13 (6.5%) - Retinoblastoma: N=2 (1.0%) - Renal tumour: N=10 (5.0%) - Hepatic tumour: N=7 (3.5%) - Bone tumour: N=18, (9.0%) - Soft tissue sarcoma: N=14 (7.0%) - Germ cell tumour: N=11 (5.5%) - Other (including adrenal gland carcinoma, nasopharyngeal carcinoma, primary adnexal carcinoma, mucoepidermoid carcinoma, and adrenocortical carcinoma; N=5 (2.5%)   Age at diagnosis  Mean age 7.3 years (SD 5.2)  Age at follow-up  Mean age 23.4 (SD 8.8)  Cancer treatment   - Chemotherapy: N=185 (92.5%) - Radiation therapy: N=130 (65.0%) - Surgery: N=85 (42.5%) - Hematopoietic stem cell transplantation: N=30 (15.0%)   Controls/normal population (if applicable)  Not applicable.  Additional participant characteristics (if applicable):  Not applicable. | Health behaviour outcomes  Physical activity, balanced diet, sun protection, smoking, and alcohol use were measured using a version of the 2013 National Youth Risk Behaviour Survey that had been modified and translated into Chinese (no other information provided).  Other variables  Not applicable | Associations between factors and smoking and alcohol consumption  All models were adjusted for age at interview, age at diagnosis, and sex.  Physical activity more than ≥4 days per week  *Age at interview (continuous)*  OR = 0.99, 95% CI (0.95-1.04), p = 0.72  *Age at diagnosis (continuous)*  OR = 0.94, 95% CI (0.86-1.02), p = 0.14  *Sex (male = ref.)*  Female: OR = 0.84, 95% CI (0.39-1.83), p = 0.67  *Clinical diagnosis (other solid malignancies = ref.)*   - Haematological malignancies: OR = 2.16, 95% CI (0.90-5.18), p = 0.085 - CNS malignancies: OR = 1.63, 95% CI (0.30-8.82), p = 0.57   *Education level* *(above secondary school = ref.)*  Secondary school or below: OR = 0.52, 95% CI (0.15-1.85), p = 0.31  *Private medical insurance (yes = ref.)*  No: OR = 0.70, 95% CI (0.32-1.54), p = 0.37  *Monthly household income (HKD, > $30,000 = ref.)*  ≤$30,000: OR = 1.07, 95% CI (0.49-2.33), p = 0.87  Current alcohol consumption  *Age at interview (continuous)*  OR = 0.98, 95% CI (0.93-1.04), p = 0.484  *Age at diagnosis (continuous)*  OR = 1.06, 95% CI (0.98-1.14), p = 0.131  *Sex (male = ref.)*  Female: OR = 0.32, 95% CI (0.15-0.67), p = 0.003  *Clinical diagnosis (other solid malignancies = ref.):*   - Haematological malignancies: OR = 0.76, 95% CI (0.36-1.62), p = 0.481 - CNS malignancies: OR = 2.33, 95% CI (0.59-9.22), p = 0.229   *Educational level (above secondary school = ref.)*   - Secondary school or below: OR = 0.98, 95% CI (0.39-2.46), p = 0.969   *Private medical insurance (yes = ref.):*   - No: OR = 0.42, 95% CI (0.20-0.85), p = 0.017   *Monthly household income (HKD, $30000 = ref.):*   - ≤$30 000: OR = 0.51, 95% CI (0.25-1.07), p = 0.076   Current and ever-smokers  *Age at interview (continuous)*  OR = 1.04, 95% CI (0.95-1.13), p = 0.426  *Age at diagnosis (continuous)*  OR = 0.97, 95% CI (0.87-1.09), p = 0.634  *Sex (male = ref.)*  OR = 0.48, 95% CI (0.14-1.62), p =0.236  *Clinical diagnosis (other solid malignancies = ref.):*   - Haematological malignancies: OR = 0.53, 95% CI (0.16-1.79), p = 0.304 - CNS malignancies: OR = 1.90, 95% CI (0.32-11.40), p = 0.485   *Education level (above secondary school = ref.)*   - Secondary school or below: OR = 5.13, 95% CI (1.48-17.75), p = 0.010   *Private medical insurance (yes = ref.):*   - No: OR = 0.31, 95% CI (0.10-1.01), p = 0.053   *Monthly household income (HKD, $30000 = ref.):*   - ≤$30 000: OR = 0.38, 95% CI (0.09-1.54), p = 0.175   Sunscreen protection use more than ≥4 days per week  *Age at interview (continuous)*  OR = 1.03, 95% CI (0.98-1.08), p = 0.27  *Age at diagnosis (continuous)*  OR = 1.05, 95% CI (0.97-1.14), p = 0.26  *Sex (male = ref.)*  OR = 5.66, 95% CI (2.40-13.34), p < 0.001  *Clinical diagnosis (other solid malignancies = ref.):*   - Haematological malignancies: OR = 0.68, 95% CI (0.30-1.54), p = 0.34 - CNS malignancies: OR = 1.19, 95% CI (0.28-5.11), p = 0.81   *Education level (above secondary school = ref.)*   - Secondary school or below: OR = 0.20, 95% CI (0.05-0.83), p = 0.026   *Private medical insurance (yes = ref.):*   - No: OR = 0.52, 95% CI (0.24-1.15), p = 0.10   *Monthly household income (HKD, $30000 = ref.):*   - ≤$30 000: OR = 0.35, 95% CI (0.15-0.84), p = 0.018   Balanced diet more than ≥4 days per week  *Age at interview (continuous)*  OR = 0.95, 95% CI (0.91-0.99), p = 0.009  *Age at diagnosis (continuous)*  OR = 1.00, 95% CI (0.94-1.07), p = 0.97  *Sex (male = ref.)*  Female: OR = 1.03, 95% CI (0.57-1.85), p = 0.924  *Clinical diagnosis (other solid malignancies = ref.)*   - Haematological malignancies: OR = 2.45, 95% CI (1.29-4.68), p = 0.006 - CNS malignancies: OR = 0.44, 95% CI (0.09-2.19), p = 0.31   *Education level* *(above secondary school = ref.)*  Secondary school or below: OR = 0.42, 95% CI (0.16-1.11), p = 0.078  *Private medical insurance (yes = ref.)*  No: OR = 0.85, 95% CI (0.47-1.54), p = 0.60  *Monthly household income (HKD, > $30,000 = ref.)*   - ≤$30 000: OR = 1.09, 95% CI (0.60-1.97), p = 0.78 |

Abbreviations: CI, confidence interval, OR, odds ratio, SD, standard deviation

| ***Florin et al.*** Physical Inactivity in Adult Survivors of Childhood Acute Lymphoblastic Leukaemia: A Report from the Childhood Cancer Survivor Study. Cancer Epidemiol Biomarkers Prev 2007, 7: 1356-1363. | | | |
| --- | --- | --- | --- |
| **Study design**  **Treatment era**  **Years of follow-up** | **Participants** | **Variable definitions** | **Results** |
| Study Design  Retrospective cohort study with longitudinal follow-up abstracted from the CCSS database.  Treatment Era  Diagnosis between 1970-1986  Follow-up Duration  Mean interval between diagnosis and completion of questionnaire: 23.1 years | Type and number of participants  N=2648 childhood cancer survivors  Cancer diagnosis  Acute lymphoblastic leukaemia  Age at diagnosis  Mean age: 5.6 years (range 0.1 to 20.0)  Age at follow-up  Age at interview:   - 18-24 years: N=788 (29.8%) 18,359 (16.6) <0.001 - 25-34 years: N=1375 (51.9%) 40,238 (36.4) - 35-44 years: N=485 (18.3%) 52,026 (47.0)   Mean age: 28.7 years (range 18 to 44)  Cancer treatment  *Chemotherapy*   - Cytarabine: N= 1286 (53.0%) - Cyclophosphamide: N= 1232 (50.8%) - Daunorubicin: N= 697 (28.7%) - Dexamethasone: N= 295 (12.2%) - Doxorubicin: N= 673 (27.8%) - Thioguanine: N= 365 (15.1%) - Etoposide: N= 365 (15.1%)   *Anthracyclines*   - None: N= 1,301 (55.0%) - <300 mg/m^2^: N= 662 (28.0%) - ≥300 mg/m^2^: N= 404 (17.0%)   *Cranial Radiation Therapy*   - None: N= 810 (35.5%) - 10.0-19.9 Gy: N= 695 (30.4%) - ≥20 Gy: N= 778 (34.1%)   Controls/normal population (if applicable)  Participants in the BRFSS survey were used as controls (N=110623).  Age at interview:   - 18-24 years: N=18359 (16.6%) - 25-34 years: N=40238 (36.4%) - 35-44 years: N=52026 (47.0%)   Additional participant characteristics (if applicable):  Not applicable | Physical activity measures  *Physical activity*  In the BRFSS survey, patients were asked 6 questions to quantify the amount of time (days/week and minutes/day) spent in moderate to vigorous physical activity during a typical week.   - Vigorous activity: any activity causing considerable increases in breathing or heart rate (e.g., aerobics, wheelchair basketball, heavy yard work.) - Moderate activity: any activity causing small increases in breathing or heart rate (e.g., brisk walking, bicycling, vacuuming, gardening, manual wheelchair operation.)   From these responses, the following outcomes were derived.   1. Meeting or not meeting CDC recommendations (≥30 minutes/day of moderate intensity physical activity for ≥5 days/week or ≥20 minutes/occasion.) 2. Inactive: reporting no leisure-time physical activity 1 month prior to survey.   Other variables  Not applicable | Associations between factors and physical activity  Not meeting CDC recommendations, adjusted for age at time of study:  *Survivors (vs. controls), p < .001*  OR = 1.44, 95% CI (1.32-1.57)  *Sex (vs. male), p < .001*  Female: OR = 1.16, 95% CI (1.13-1.19)  *Race (vs. Non-Hispanic White), p < .001*   - Black: OR = 1.52, 95% CI (1.46-1.60) - Other: OR = 1.15, 95% CI (1.10-1.20) - Hispanic: OR = 1.41, 95% CI (1.35-1.47)   *Income (US$, ≥ 20,000 = ref.), p < .001*  < 20,000: OR = 1.24, 95% CI (1.20-1.29)  *Education (vs. college graduate), p < .001*   - Some high school: OR = 1.49, 95% CI (1.41-1.57) - Graduated high school: OR = 1.26, 95% CI (1.22-1.31) - Some college: OR = 1.11, 95% CI (1.07-1.15)   *Current smoker (no = ref.), p = .54*  Yes: OR = 1.00, 95% CI (0.98-1.04)  Adjusted for age, race, and BMI:  *Females treatment (controls = ref.)*   - Chemo only: OR = 1.31, 95% CI (1.10-1.59), p = .006 - Chemo + CRT < 20 Gy: OR = 1.44, 95% CI (1.15-1.80), p = .001 - Chemo + CRT ≥ 20 Gy: OR = 2.07 (1.67-2.56), p < .001   *Males treatment (controls = ref.)*   - Chemo only: OR = 0.96, 95% CI (0.77-1.19), p = .69 - Chemo + CRT < 20 Gy: OR = 1.37, 95% CI (1.11-1.69), p = .004 - Chemo + CRT ≥ 20 Gy: OR = 1.43 (1.16-1.76), p = .002   No leisure-time physical activity (inactive), adjusted for age at time of study:  *Survivors (vs. controls), p < .001*  OR = 1.74, 95% CI (1.56-1.94)  *Sex (vs. male), p < .001*  Female: OR = 1.33, 95% CI (1.29-1.38)  *Race (vs. Non-Hispanic White), p < .001*   - Black: OR = 1.73, 95% CI (1.64-1.82) - Other: OR = 1.32, 95% CI (1.24-1.40) - Hispanic: OR = 1.91, 95% CI (1.81-2.00)   *Income (US$, ≥ 20,000 = ref.), p < .001*  < 20,000: OR = 1.66, 95% CI (1.59-1.72)  *Education (vs. college graduate), p < .001*   - Some high school: OR = 3.78, 95% CI (3.55-4.01) - Graduated high school: OR = 2.52, 95% CI (2.41-2.63) - Some college: OR = 1.54, 95% CI (1.47-1.62)   *Current smoker (no = ref.), p < .001*  Yes: OR = 1.37, 95% CI (1.32-1.42)  Adjusted for age, race, and BMI:  *Females treatment (controls = ref.)*   - Chemo only: OR = 1.06, 95% CI (0.84-1.35), p = .62 - Chemo + CRT < 20 Gy: OR = 1.37, 95% CI (1.06-1.78), p = .10 - Chemo + CRT ≥ 20 Gy: OR = 1.86 (1.50-2.31), p < .001   *Males treatment (controls = ref.)*   - Chemo only: OR = 1.10, 95% CI ((0.83-1.46), p = .52 - Chemo + CRT < 20 Gy: OR = 1.68, 95% CI (1.30-2.16), p < .001 - Chemo + CRT ≥ 20 Gy: OR = 1.84 (1.45-2.32), p < .001 |

Abbreviations: BMI, body mass index, BRFSS, Behavioural Risk Factor Surveillance System, CCSS, childhood cancer survivor study, CDC, Centers for Disease Control and Prevention, CI, confidence interval, Gy, gray, OR, odds ratio, SD, standard deviation

| ***Ness et al.*** Predictors of inactive lifestyle among adult survivors of childhood cancer: A report from the Childhood Cancer Survivor Study. Cancer 2009, 9:1984.1994. | | | |
| --- | --- | --- | --- |
| **Study design**  **Treatment era**  **Years of follow-up** | **Participants** | **Variable definitions** | **Results** |
| Study Design  Retrospective cohort study with longitudinal follow-up abstracted from the Childhood Cancer Survivor Study database.  Treatment Era  1970-1986  Follow-up Duration  2003 follow-up survey was used. Exact follow-up duration not specified. | Type and number of participants  N = 9301 childhood cancer survivors  Cancer diagnosis   - Acute lymphoblastic leukaemia: N=5381 - Acute myeloid leukaemia: N=460 - Other leukaemias: N=364 - Astrocytoma: N=1454 - Medulloblastoma: N=485 - Other central nervous system tumour: N=363 - Hodgkin lymphoma: N=2331 - Non-Hodgkin lymphoma: N=1380 - Wilms tumour: N=1710 - Neuroblastoma: N=1222 - Osteosarcoma/other bone tumour: N=1032 - Ewing sarcoma: N=467 - Soft tissue sarcoma: N=1608   Age at diagnosis  Not specified.  Age at follow-up   - 18 to 29 years: N=3843 (41.3%) - 30 to 39 years: N=3868 (41.6%) - 40 to 49 years: N=1503 (16.2%) - ≥50 years: N=87 (0.9%)   Cancer treatment  Not reported  Controls/normal population (if applicable)  N=2886 siblings   - 18 to 29 years: N=1011 (35.0%) - 30 to 39 years: N=1084 (37.6%) - 40 to 49 years: N=682 (23.6%) - ≥50 years: N=109 (3.8%)   Additional participant characteristics (if applicable):  A BRFSS group also served as a reference group for external validation (N=26979)  N=860 had amputation of lower limb. | Physical activity measures  *Physical activity guidelines*  Assessed based on answers from the BRFSS about physical activity (6 questions), and 1 question related to participation in physical activity over the previous month (yes/no; CDC guidelines for MVPA, yes/no; for inactive lifestyle if any participation in leisure physical activity).  Other variables  Not applicable | Associations between factors and physical activity  Significance indicated by 99% CIs. Models adjusted for age.  Did not meet physical activity guidelines:  *Survivors (vs. siblings)*  RR = 1.2, 99% CI (1.1-1.3)  *Gender (male = ref.)*  Female: RR = 1.2, 99% CI (1.1-1.3)  *Race (White - non-Hispanic = ref.)*   - Black – non Hispanic: RR = 1.2, 99% CI (1.2-1.3) - Hispanic: RR = 1.1, 99% CI (1.0-1.2) - Other: RR = 1.1, 99% CI (1.0-1.2)   *Age (18-29 years = ref.)*   - 30-39 years: RR = 1.1, 99% CI (1.0-1.2) - 40-49 years: RR = 1.1, 99% CI (1.0-1.2) - 50+ years: RR = 1.2, 99% CI (1.1-1.4)   *Educational attainment (< high school = ref.)*   - High school graduate: RR = 0.9, 99% CI (0.8-1.0) - College graduate: RR = 0.9, 99% CI (0.8-1.1)   *Employment (working/caring for home or family = ref.)*   - Student: RR = 0.8, 99% CI (0.7-0.9) - Unemployed/looking for work: RR = 1.0, 99% CI (0.9-1.2) - Unable to work: RR = 1.2, 99% CI (1.1-1.3)   Annual household income (< $20.000 = ref.)  ≥ $20.000: RR = 1.1, 99% CI (0.9-1.1)  *Body mass index (normal weight = ref.)*   - Underweight: RR = 1.2, 95% CI (1.1-1.3) - Overweight: RR = 1.1, 99% CI (1.0-1.2) - Obese: RR = 1.2, 99% CI (1.1-1.3)   *Smoking status (never = ref.)*   - Current: RR = 1.0, 99% CI (0.9-1.1) - Ever: RR = 0.9, 99% CI (0.8-1.0)   *Depression at time of the survey (no = ref.)*  Yes: RR = 1.0, 99% CI (0.9-1.1)  *Female cancer diagnosis (siblings = ref.)*   - Acute lymphoblastic leukaemia: RR = 1.2, 99% CI (1.1-1.3) - Acute myeloid leukaemia: RR = 1.2, 99% CI (1.0-1.4) - Other or unspecified leukaemia: RR = 1.2, 99% CI (1.0-1.4) - Astrocytoma: RR = 1.3, 99% CI (1.1-1.4) - Medulloblastoma, PNET: RR = 1.4, 99% CI (1.2-1.6) - Other CNS tumour: RR = 1.2, 99% CI (0.9-1.4) - Hodgkin lymphoma: RR = 1.0, 99% CI (0.9-1.1) - Non-Hodgkin lymphoma: RR = 1.1, 99% CI (1.0-1.3) - Wilms' tumour (kidney tumours): RR = 1.1, 99% CI (1.0-1.3) - Neuroblastoma: RR = 1.1, 99% CI (1.0-1.3) - Osteosarcoma/other bone tumour: RR = 1.2, 99% CI (1.1-1.3) - Ewing’s sarcoma: RR = 1.4, 99% CI (1.2-1.6) - Soft tissue sarcoma: RR = 1.2, 99% CI (1.1-1.3)   *Female surgery (no surgery = ref.)*   - Amputation of lower limb: RR = 1.3, 99% CI (1.1-1.5) - Other surgery: RR = 1.1, 99% CI (1.0-1.2)   *Female chemotherapy* (no chemotherapy = ref.)   - Chemotherapy including anthracyclines: RR = 1.1, 99% CI (1.0-1.2) - Chemotherapy without anthracyclines: RR = 1.0, 99% CI (0.9-1.1)   *Female radiation (no radiation = ref.)*   - Any cranial radiation: RR = 1.2, 99% CI (1.1-1.3) - Chest radiation without cranial radiation: RR = 1.0, 99% CI (0.9-1.1) - Other radiation: RR = 1.1, 99% CI (1.0-1.2)   *Male cancer diagnosis (siblings = ref.)*   - Acute lymphoblastic leukaemia: RR = 1.1, 99% CI (1.0-1.2) - Acute myeloid leukaemia: RR = 1.1, 99% CI (0.9-1.3) - Other or unspecified leukaemia: RR = 1.1, 99% CI (0.9-1.3) - Astrocytoma: RR = 1.2, 99% CI (1.1-1.3) - Medulloblastoma, PNET: RR = 1.4, 99% CI (1.2-1.6) - Other CNS tumour: RR = 1.3, 99% CI (1.1-1.5) - Hodgkin lymphoma: RR = 1.0, 99% CI (0.9-1.1) - Non-Hodgkin lymphoma: RR = 1.1, 99% CI (1.0-1.3) - Wilms' tumour (kidney tumours): RR = 1.0, 99% CI (0.9-1.2) - Neuroblastoma: RR = 1.1, 99% CI (1.0-1.3) - Osteosarcoma/other bone tumour: RR = 1.2, 99% CI (1.1-1.3) - Ewing’s sarcoma: RR = 1.0, 99% CI (0.9-1.3) - Soft tissue sarcoma: RR = 1.1, 99% CI (1.0-1.2)   *Male surgery (no surgery = ref.)*   - Amputation of lower limb: RR = 1.3, 99% CI (1.1-1.5) - Other surgery: RR = 1.1, 99% CI (1.0-1.2)   *Male chemotherapy* (no chemotherapy = ref.)   - Chemotherapy including anthracyclines: RR = 1.0, 99% CI (0.9-1.1) - Chemotherapy without anthracyclines: RR = 1.0, 99% CI (0.9-1.1)   *Male radiation (no radiation = ref.)*   - Any cranial radiation: RR = 1.2, 99% CI (1.1-1.3) - Chest radiation without cranial radiation: RR = 1.1, 99% CI (1.0-1.2) - Other radiation: RR = 1.0, 99% CI (0.9-1.1)   Inactive lifestyle  *Survivors (vs. siblings)*  RR = 1.6, 99% CI (1.4-1.8)  *Gender (male = ref.)*  Female: RR = 1.2, 99% CI (1.1-1.3)  *Race (White - non-Hispanic = ref.)*   - Black – non Hispanic: RR = 1.7, 99% CI (1.3-2.2) - RR = 1.1, 99% CI (0.9-1.5) - RR = 1.2, 99% CI (0.8-1.6)   *Age (18-29 years = ref.)*   - 30-39 years: RR = 1.5, 99% CI (1.3-1.7) - 40-49 years: RR = 1.5, 99% CI (1.3-1.8) - 50+ years: RR = 2.0, 99% CI (1.4-3.0)   *Educational attainment (< high school = ref.)*   - High school graduate: RR = 0.8, 99% CI (0.6-1.0) - College graduate: RR = 0.4, 99% CI (0.3-0.6)   *Employment (working/caring for home or family = ref.)*   - Student: RR = 0.9, 99% CI (0.7-1.2) - Unemployed/looking for work: RR = 1.3, 99% CI (1.0-1.6) - Unable to work: RR = 2.1, 99% CI (1.7-2.5)   Annual household income (< $20.000 = ref.)  ≥ $20.000: RR = 0.8, 99% CI (0.7-0.9)  *Body mass index (normal weight = ref.)*   - Underweight: RR = 1.5, 99% CI (1.2-1.9) - Overweight: RR = 1.0, 99% CI (0.9-1.2) - Obese: RR = 1.4, 99% CI (1.3-1.7)   *Smoking status (never = ref.)*   - Current: RR = 1.5, 99% CI (1.2-1.9) - Ever: RR = 1.0, 99% CI (0.8-1.1)   *Depression at time of the survey (no = ref.)*  Yes: RR = 1.4, 99% CI (1.2-1.7)  *Female cancer diagnosis (siblings = ref.)*   - Acute lymphoblastic leukaemia: RR = 1.9, 99% CI (1.6-2.2) - Acute myeloid leukaemia: RR = 1.4, 99% CI (1.0-2.0) - Other or unspecified leukaemia: RR = 1.9, 99% CI (1.3-2.8) - Astrocytoma: RR = 1.9, 99% CI (1.6-2.4) - Medulloblastoma, PNET: RR = 3.0, 99% CI (2.4-4.0) - Other CNS tumour: RR = 2.3, 99% CI (1.6-3.2) - Hodgkin lymphoma: RR = 1.3, 99% CI (1.1-1.6) - Non-Hodgkin lymphoma: RR = 1.7, 99% CI (1.3-2.1) - Wilms' tumour (kidney tumours): RR = 1.6, 99% CI (1.3-2.0) - Neuroblastoma: RR = 1.4, 99% CI (1.1-1.8) - Osteosarcoma/other bone tumour: RR = 1.9, 99% CI (1.5-2.4) - Ewing’s sarcoma: RR = 1.5, 99% CI (1.1-2.2) - Soft tissue sarcoma: RR = 1.6, 99% CI (1.3-2.0)   *Male cancer diagnosis (siblings = ref.)*   - Acute lymphoblastic leukaemia: RR = 1.6, 99% CI (1.3-1.9) - Acute myeloid leukaemia: RR = 1.6, 99% CI (1.0-2.3) - Other or unspecified leukaemia: RR = 1.5, 99% CI (1.0-2.3) - Astrocytoma: RR = 1.9, 99% CI (1.5-2.3) - Medulloblastoma, PNET: RR = 2.3, 99% CI (1.7-3.0) - Other CNS tumour: RR = 2.3, 99% CI (1.7-3.2) - Hodgkin lymphoma: RR = .3, 99% CI (1.1-1.7) - Non-Hodgkin lymphoma: RR = 1.6, 99% CI (1.3-2.0) - Wilms' tumour (kidney tumours): RR = 1.6, 99% CI (1.2-2.0) - Neuroblastoma: RR = 1.5, 99% CI (1.1-2.0) - Osteosarcoma/other bone tumour: RR = 1.6, 99% CI (1.2-2.1) - Ewing’s sarcoma: RR = 1.3, 99% CI (0.9-2.0) - Soft tissue sarcoma: RR = 1.6, 99% CI (1.3-2.0)   *Female surgery (no surgery = ref.)*   - Amputation of lower limb: RR = 1.6, 99% CI (1.2-2.0) - Other surgery: RR = 1.2, 99% CI (1.0-1.4)   *Female chemotherapy* (no chemotherapy = ref.)   - Chemotherapy including anthracyclines RR = 1.1, 99% CI (1.0-1.3) - Chemotherapy without anthracyclines: RR = 1.1, 99% CI (1.0-1.3)   *Female radiation (no radiation = ref.)*   - Any cranial radiation: RR = RR = 1.5, 99% CI (1.3-1.7) - Chest radiation without cranial radiation: RR = 1.0, 99% CI (0.8-1.2) - Other radiation: RR = 1.1, 99% CI (0.9-1.3)   *Male surgery (no surgery = ref.)*   - Amputation of lower limb: RR = 1.4, 99% CI (1.0-1.9) - Other surgery: RR = 1.0, 99% CI (0.9-1.3)   *Male chemotherapy* (no chemotherapy = ref.)   - Chemotherapy including anthracyclines: RR = 0.8, 99% CI (0.7-1.0) - Chemotherapy without anthracyclines: RR = 0.9, 99% CI (0.8-1.1)   *Male radiation (no radiation = ref.)*   - Any cranial radiation: RR = 1.3, 99% CI (1.1-1.6) - Chest radiation without cranial radiation: RR = 1.0, 99% CI (0.9-1.3) - Other radiation: RR = 1.0, 99% CI (0.8-1.2) |

Abbreviations: BRFSS, Behavioural Risk Factor Surveillance System, CI, confidence interval, CDC, Centers for Disease Control and Prevention, CNS=Central Nervous System, MVPA, moderate to vigorous physical activity, PNET=Primitive Neuroectodermal Tumour, RR = risk ratio

| ***Slater et al.*** Active transportation in adult survivors of childhood cancer and neighborhood controls. J Cancer Surviv 2016, 10: 11-20. | | | |
| --- | --- | --- | --- |
| **Study design**  **Treatment era**  **Years of follow-up** | **Participants** | **Variable definitions** | **Results** |
| Study Design  Cross-sectional  Treatment Era  Not reported  Follow-up Duration  Mean time since diagnosis: 18.4 (SE 0.7) years. | Type and number of participants  N=158 childhood cancer survivors  Cancer diagnosis   - Leukaemia (acute lymphoblastic leukaemia, acute myeloid leukaemia): N=56 (35.4%) - Lymphoma (Hodgkin lymphoma, non-Hodgkin lymphoma): N=31 (19.6%) - Osteosarcoma: N=19 (12.0%) - Central nervous system: N=16 (10.1%) - Other: N=36 (22.8%)   Age at diagnosis  Not reported  Age at follow-up  Mean age 29 (SE 0.6) years  Cancer treatment  Not reported  Controls/normal population (if applicable)  N=153 age, sex, and neighbourhood-matched controls  Additional participant characteristics (if applicable):  Not applicable | Physical activity measures  *Transportation-related activity*  The International Physical Activity Questionnaire was modified with other questionnaires (Neighbourhood Environment Walkability Scale, Transportation-Related Activities of Childhood Cancer Survivors) to self-report transportation-related activity levels (bicycling/walking to/from school/work, run errands, or general travel) or other forms of activity (job-related, household activities, leisure-time activity). Seasonal variability was considered/adjusted accordingly.  Other variables  *Psychosocial barriers*  Assessed via questionnaire, asking whether survivors disproportionately experience barriers related to cancer-related disease/treatment.  *Objective walkability*  An objectively measured neighbourhood walkability score, called Walk Score, was obtained on a publicly available website (available at: <http://www.walkscore.com/>), yielding a score from 0 to 100, with higher scores indicating greater walkability. | Associations between factors and physical activity  Odds of engaging in active transportation  Models adjusted for BMI, income, and current smoking. None of the results were found to be significant unless indicated.  *Age (continuous years)*  OR = 0.95, 95% CI (0.88–1.02)  *Body mass index (continuous kg/m2)*  OR = 0.93, 95% CI (0.85–1.02)  *Married or living with a partner (no = ref.), p < .05*  OR = 0.30, 95% CI (0.11–0.81)  *Vehicles per driver (continuous)*  OR = 0.14, 95% CI (0.01–1.33)  *Environmental barriers*  OR = 1.61, 95% CI (0.59–4.43)  *Planning/psychosocial barriers, p < .05*  OR = 0.15, 95% CI (0.04–0.53)  *Health barriers*  OR = 1.15, 95% CI (0.61–2.16)  All barriers were measured on four-point Likert scales, where 1=strongly disagree, 2=somewhat disagree, 3=somewhat agree, and 4=strongly agree that a particular  barrier makes it difficult to actively travel.  *Perceived walkability, p < .05*  OR = 2.55, 95% CI (1.14–5.66)  Scores range from 1 to 5, with higher scores indicating greater perceived walkability.  *Objective walkability*  OR = 1.01, 95% CI (0.98–1.03)  Walk Scores range from 0 to 100, with higher scores indicating greater walkability. |

Abbreviations: CI, confidence interval, OR, odds ratio, SE, standard error

| ***Rueegg et al.*** Daily Physical Activities and Sports in Adult Survivors of Childhood Cancer and Healthy Controls: A Population-Based Questionnaire Survey, 2012, PLoS One, 7(4):e34930. | | | |
| --- | --- | --- | --- |
| **Study design**  **Treatment era**  **Years of follow-up** | **Participants** | **Variable definitions** | **Results** |
| Study design  Cohort study  Treatment era  Diagnosis between 1976-2003.  Follow-up duration  Time since diagnosis:   - 5–9.9 years: N=93 (8.78%) - 10–19.9 years: N=471 (44.57%) - 20–29.9 years: N=427 (40.39%) - ≥30 years: N=67 (6.34%) | Type and number of participants  N=1058 childhood cancer survivors  Cancer diagnosis   - Leukaemia: N=394 (37%) - Lymphoma: N=219 (21%) - Central nervous system tumour: N=121 (11%) - Neuroblastoma: N=36 (3%) - Retinoblastoma: N=21 (2%) - Renal tumour: N=60 (6%) - Hepatic tumour: N=7 (1%) - Bone tumour: N=54 (5%) - Soft tissue sarcoma: N=58 (6%) - Germ cell tumour: N=28 (3%) - Langerhans cell histiocytosis: N=44 (4%) - Other (malignant epithelial neoplasm, malignant melanomas and other or unspecified malignant neoplasm): N=13 (1%)   Age at diagnosis   - 0–4.9 years: N=338 (32%) - 5–9.9 years: N=279 (26%) - ≥10 years: N=441 (42%)   Cancer treatment   - Surgery only: N=98 (9%) - Chemotherapy (may include surgery): N=491 (47%) - Radiotherapy (may include surgery or chemotherapy): N=378 (36%) - Bone marrow transplantation: N=87 (8%)     Age at follow-up   - ≤ 24.9 years: N=417 (39.42%) - 25–29.9 years: N=304 (28.76%) - 30–34.9 years: N=188 (17.80%) - ≥35 years: N=149 (14.03%)   Controls/normal population (if applicable)  N=5593 population controls; weighted proportions and numbers of the Swiss normal population were used according to the marginal distribution in survivors on: age, gender, language region, and  nationality.  Age:   - <24.9 years: N=2237 (40.04%) - 25–29.9 years: N=1678 (30.04%) - 30–34.9 years: N=1007 (18.03%) - ≥35 years: N=671 (12.00%)   Additional participant characteristics (if applicable):  Not applicable | Physical activity measures  *Daily activities*  All participants were asked on how many days per week they engage in any type of activities that make them sweat (vigorous activities). Survivors were also asked how many minutes per day they engage in such activities. Then, participants were asked how many days per week and minutes per day they engage in activities that cause some increase in breathing (moderate activities). People were then classified as ‘‘active’’ (vs. ‘‘inactive’’) if they engaged in moderate activities for ≥30 minutes on ≥ 5 days a week or in vigorous activities on ≥ 3 days a week, according to the international physical activity recommendations of the Center of Disease Control and Prevention.  *Sporting activities*  Participants were asked whether they  engage in any gymnastics, fitness training or sports; how intensely they practice these sports; and how many hours per week. People were then classified as doing ‘‘sports’’ (vs. ‘‘no sports’’) if they engaged in a targeted gym or sport at least somewhat intense and at least one hour per week. Survivors were also asked to list the types of sport they were doing.  Other variables  Not applicable. | Factors associated with physical activity  Clinical factors were adjusted for all socio-demographic and cultural factors.  Risk factors for inactivity:  *Current age (0-24.9 years = ref.), p = 0.131*   - 25–29.9 years: OR = 1.49, 95% CI (1.05 - 2.11) - 30–34.9 years: OR = 1.46, 95% CI 0.93 - 2.29,) - ≥35 years: OR = 1.31, 95% CI (0.77 - 2.24)   *Gender (male = ref.), p = .001*  Female: OR = 1.66, 95% CI (1.24 - 2.22)  *Migration background (no = ref.), p = .336*  Yes: OR = 1.18, 95% CI (0.84 - 1.64)  *Education (vocational training = ref.), p = .017*   - Compulsory schooling: OR = 1.87, 95% CI (1.07 - 3.27) - Upper secondary education: OR = 1.43, 95% CI (1.05 - 1.95) - University education: OR = 1.83, 95% CI (1.03 - 3.25)   *Civil status (single, divorced, other = ref.), p = .967*  Married: OR = 1.01, 95% CI (0.60 - 1.70)  *Children (no = ref.), p = .248*  Yes: OR = 1.40, 95% CI (0.79 - 2.48)  *BMI categories (kg/m²; normal weight ≥18/<25 = ref.), p = .037*   - Underweight (<18): OR = 3.02, 95% CI (1.34 - 6.80) - Overweight (≥25/<30): OR = 1.01, 95% CI (0.71 - 1.45) - Obese (≥30): OR = 1.46, 95% CI (0.81 - 2.63)   *Smoking (current non-smoker = ref.), p = .255*  Current smoker: OR = 1.21, 95% CI (0.87 - 1.69)  *Age at diagnosis (0–4.9 years = ref.), p = .205*   - 5–9.9 years: OR = 1.25, 95% CI (0.85 - 1.83) - ≥10 years: OR = 1.42, 95% CI (0.96 - 2.10)   *Treatment (chemotherapy = ref.), p = .784*   - Surgery only: OR = 0.81, 95% CI (0.42 - 1.54) - Radiotherapy: 50% (OR = 0.86, 95% CI 0.60 - 1.22) - BMT: OR = 1.04, 95% CI (0.61 - 1.76)   *Diagnosis (leukaemia = ref.),*  *p = 0.639*   - Lymphomas: OR = 1.06, 95% CI (0.71 - 1.58) - CNS tumours: OR = 0.91, 95% CI (0.48 - 1.72) - Neuroblastomas: OR = 1.64, 95% CI (0.74 - 3.63) - Retinoblastomas: OR = 1.44, 95% CI (0.47 - 4.36) - Renal & hepatic tumours: OR = 1.02, 95% CI (0.55 - 1.89) - Bone tumours: OR = 1.34, 95% CI (0.69 - 2.59) - Soft tissue sarcomas: OR = 1.79, 95% CI (0.91 - 3.53) - Germ cell tumours: OR = 1.64, 95% CI (0.66 - 4.06) - Langerhans cell histiocytosis: OR = 0.69, 95% CI (0.30 - 1.55) - Other: OR = 1.46, 95% CI (0.41 - 5.16)   *Self-reported late effects (no late effects = ref.), p = .116*   - Somatic late effects: OR = 1.41, 95% CI (0.99 - 2.00) - Psychological late effects: OR = 0.93, 95% CI (0.57 - 1.51)   No sports  *Current age (0-24.9 years = ref.), p = 0.871*   - 25–29.9 years: OR = 1.11, 95% CI (0.77 - 1.58) - 30–34.9 years: OR = 1.19, 95% CI 0.76 - ≥35 years: OR = 1.06, 95% CI (0.62 - 1.82)   *Gender (male = ref.), p = .601*  Female: OR = 0.92, 95% CI (0.69 - 1.24)  *Migration background (no = ref.), p = .084*  Yes: OR = 1.34, 95% CI (0.96 - 1.85)  *Education (vocational training = ref.), p = .016*   - Compulsory schooling: OR = 1.72, 95% CI (1.01 - 2.93) - Upper secondary education: OR = 0.85, 95% CI (0.62 - 1.17) - University education: OR = 0.51, 95% CI (0.27 - 0.97)   *Civil status (single, divorced, other = ref.), p = .967*  Married: OR = 1.05, 95% CI (0.62 - 1.77)  *Children (no = ref.), p = .045*  Yes: OR = 1.79, 95% CI (1.01 - 3.17)  *BMI categories (kg/m²; normal weight ≥18/<25 = ref.), p = .044*   - Underweight (<18): OR = 1.23, 95% CI (0.60 - 2.57) - Overweight (≥25/<30): OR = 0.99, 95% CI (0.69 - 1.42) - Obese (≥30): OR = 2.28, 95% CI (1.27 - 4.10)   *Smoking (current non-smoker = ref.), p < .001*   - Current smoker: OR = 1.87, 95% CI (1.35 - 2.58)   *Age at diagnosis (0–4.9 years = ref.), p = .167*   - 5–9.9 years: OR = 0.73, 95% CI (0.50 - 1.09) - ≥10 years: OR = 1.01, 95% CI (0.69 - 1.50)   *Treatment (chemotherapy = ref.), p = .784*   - Surgery only: OR = 0.66, 95% CI (0.35 - 1.27) - Radiotherapy: OR = 0.99, 95% CI (0.70 - 1.42) - BMT: OR = 1.47, 95% CI (0.97 - 2.46)   *Diagnosis (leukaemia = ref.),*  *p = 0.087*   - Lymphomas: OR = 0.87, 95% CI (0.58 - 1.31) - CNS tumours: OR = 1.59, 95% CI (0.86 - 2.96) - Neuroblastomas: OR = 1.82, 95% CI (0.82 - 4.03) - Retinoblastomas: OR = 1.76, 95% CI (0.59 - 5.26) - Renal & hepatic tumours: OR = 0.66, 95% CI (0.33 - 1.32) - Bone tumours: OR = 2.01, 95% CI (1.05 - 3.87) - Soft tissue sarcomas: OR = 1.14, 95% CI (0.59 - 2.22) - Germ cell tumours: OR = 2.22, 95% CI (0.93 - 5.28) - Langerhans cell histiocytosis: OR = 0.87, 95% CI (0.38 - 1.95) - Other: OR = 0.64, 95% CI (0.16 - 2.60)   *Self-reported late effects (no late effects = ref.), p = .116*   - Somatic late effects: OR = 1.11, 95% CI (0.94 - 1.89) - Psychological late effects: OR = 1.02, 95% CI (0.63 - 1.66)   Language region was also included in the model but not reported in this table. |

Abbreviations: BMT, bone marrow transplantation, CI, confidence interval, CNS, central nervous system, OR odds ratio

| ***Rueegg et al.*** Physical Performance Limitations in Adolescent and Adult Survivors of Childhood Cancer and Their Siblings, 2012, PLoS One, 7(10): e47944. | | | |
| --- | --- | --- | --- |
| **Study design**  **Treatment era**  **Years of follow-up** | **Participants** | **Variable definitions** | **Results** |
| Study design  Cohort study  Treatment era  Diagnosis between 1976-2003.  Follow-up duration  Time since diagnosis:   - < 10 years: N=134 (12.9%) - 10–19.9 years: N=483 (46.5%) - 20–29.9 years: N=351 (33.8%) - ≥30 years: N=70 (6.7%) | Type and number of participants  N=1038 childhood cancer survivors  Cancer diagnosis   - Leukaemia: N=383 (36.9%) - Lymphoma: N=195 (18.8%) - Central nervous system tumour: N=132 (12.7%) - Neuroblastoma: N=45 (4.3%) - Retinoblastoma: N=21 (2.0%) - Renal tumour: N=70 (6.7%) - Hepatic tumour: N=5 (0.5%) - Bone tumour: N=42 (4.1%) - Soft tissue sarcoma: N=56 (5.4%) - Germ cell tumour: N=30 (2.9%) - Langerhans cell histiocytosis: N=47 (4.5%) - Other (malignant epithelial neoplasm, malignant melanomas and other or unspecified malignant neoplasm): N=12 (1.2%)   Age at diagnosis   - 0–4.9 years: N=372 (35.8%) - 5–9.9 years: N=286 (27.6%) - ≥10 years: N=380 (36.6%)   Cancer treatment   - Surgery: N=698 (67.2%) - Chemotherapy: N=871 (83.9%) - Radiotherapy: N=393 (37.9%) - Bone marrow transplantation: N=80 (7.7%)     Age at follow-up   - < 20 years: N=234 (22.5%) - 20 –29.9 years: N=536 (51.6%) - 30–39.9 years: N=228 (22.0%) - ≥40 years: N=40 (3.9%)   Controls/normal population (if applicable)  N=534 sibling participants. Age:   - < 20 years: N=110 (20.6%) - 20 –29.9 years: N=293 (54.9%) - 30–39.9 years: N=111 (20.8%) - ≥40 years: N=20 (3.8%)   Additional participant characteristics (if applicable):  Not applicable | Physical activity measures  *Limitations in sports*  Assessed by asking participants whether or not they had ‘‘any limitation in sporting activities’’. If so, they were asked to describe the limitation in detail. Three paediatricians manually coded these answers into broad categories of medical conditions. When participants reported more than one problem, the most severe was used for analysis.  *Limitations in daily activities*  Defined as low physical function in the SF-36. The physical function score aggregates ten questions related to tasks of daily living, such as carrying groceries, climbing stairs, bending down, walking a certain distance, dressing or bathing. Raw scores were converted to T scores (mean = 50, SD = 10) according to age- and sex-stratified norm data from a public use-file from the German Federal Survey (N = 6964). A natural cut-off value below the 5th percentile of the distribution of the sibling population was used. Survivors below this cut-off were defined as ‘‘limited in daily activities’’.    Other variables  Not applicable. | Risk factors for limitations in sports  All models adjusted for age and sex. In a sensitivity analysis, the regression model was also adjusted for family clustering.  *Current age (20-29.9 years = ref.), p = 0.061*   - ≤ 20 years: OR = 1.13, 95% CI (0.62 - 2.07) - -30–39.9 years: OR = 0.63, 95% CI (0.32 - 1.22) - ≥40 years: OR = 2.70, 95% CI (1.02 - 7.16)   *Gender (male = ref.), p = 0.367*  Female: OR = 1.23, 95% CI (0.78 - 1.95)  *Parents education (secondary education = ref.), p = 0.035*   - Primary education: OR = 0.40, 95% CI (0.13 - 1.18) - Tertiary education: OR = 0.98, 95% CI (0.51 - 1.87) - Unknown: OR = 2.97, 95% CI (1.19 - 7.39)   *Age at diagnosis (<5 years = ref.), p = 0.964*   - <5 years: OR = 1.0, 95% CI (ref.) - 5–9.9 years: OR = 1.04, 95% CI (0.55 - 1.97) - ≥10 years: OR = 1.10, 95% CI (0.56 - 2.14)   *Diagnosis (leukaemia = ref.), p < 0.001*   - Lymphoma: OR = 1.16, 95% CI (0.49 - 2.74) - CNS tumour: OR = 9.40, 95% CI (4.26 - 20.74) - Neuroblastoma: OR = 3.77, 95% CI (1.23 - 11.55) - Retinoblastoma: OR = 8.55, 95% CI (2.26 - 32.33) - Renal & hepatic tumour: OR = 1.65, 95% CI (0.57 - 4.80) - Bone tumour: OR = 13.59, 95% CI (5.55 - 33.28) - Soft tissue sarcoma: OR = 2.87, 95% CI (1.07 - 7.70) - Germ cell tumour: OR = 2.11, 95% CI (0.44 - 10.12) - Other tumour: OR = 5.72, 95% CI (1.03 - 31.76) - Langerhans Cell Histiocytosis: OR = 1.62, 95% CI (0.35 - 7.52)   *Treatment (chemotherapy = ref.), p < 0.001*   - Surgery only: OR = 0.35, 95% CI (0.13 - 0.90) - Chemotherapy: OR = 1.0, 95% CI (ref.) - Radiotherapy: OR = 1.61, 95% CI (0.90 - 2.88) - Bone marrow transplantation: OR = 0.85, 95% CI (0.22 - 3.28)   *Relapse (no = ref.), p = 0.880*  Yes: OR = 1.06, 95% CI (0.52 - 2.13)  Risk factors for limitations in daily activities  *Current age (20-29.9 years = ref.), p = 0.496*   - < 21 years: OR = 1.45, 95% CI (0.90 - 2.32) - 30–39.9 years: OR = 1.05, 95% CI (0.64 - 1.72) - ≥40 years: OR = 0.97, 95% CI (0.36 - 2.63)   *Gender (male = ref.), p = 0.001*  Female: OR = 1.31, 95% CI (0.90 - 1.90)  *Parents education (secondary education = ref.), p = 0.008*   - Primary education: OR = 1.89, 95% CI (1.03 - 3.49) - Tertiary education: OR = 1.01, 95% CI (0.58 - 1.76) - Unknown: OR = 2.80, 95% CI (1.46 - 5.38)   *Age at diagnosis (< 5 years = ref.), p = 0.387*   - 5–9.9 years: OR = 0.72, 95% CI (0.43 - 1.19) - ≥10 years: OR = 0.92, 95% CI (0.54 - 1.56)   *Diagnosis (leukaemia = ref.), p < 0.001*   - Lymphoma: OR = 0.89, 95% CI (0.45 - 1.75) - CNS tumour: OR = 5.76, 95% CI (3.08 - 10.80) - Neuroblastoma: OR = 2.42, 95% CI (0.95 - 6.17) - Retinoblastoma: OR = 2.88, 95% CI (0.82 - 10.10) - Renal & hepatic tumour: OR = 2.19, 95% CI (1.04 - 4.63) - Bone tumour: OR = 10.87, 95% CI (5.04 - 23.45) - Soft tissue sarcoma: OR = 1.76, 95% CI (0.77 - 4.04) - Germ cell tumour: OR = 1.15, 95% CI (0.31 - 4.19) - Other tumour: OR = 1.86, 95% CI (0.34 - 10.02) - Langerhans Cell Histiocytosis: OR = 2.91, 95% CI (1.15 - 7.35)   *Treatment (chemotherapy = ref.), p < 0.001*   - Surgery only: OR = 0.50, 95% CI (0.23 - 1.07) - Radiotherapy: OR = 2.08, 95% CI (1.31 - 3.32) - Bone marrow transplantation: OR = 2.98, 95% CI (1.24 - 7.14)   *Relapse (no = ref.), p = 0.326*  Yes: OR = 1.32, 95% CI (0.76 - 2.29) |

Abbreviations: CI confidence interval, CNS, central nervous system, OR odds ratio, SD, standard deviation

| ***Lown et al.***  Alcohol consumption patterns and risk factors among childhood cancer survivors compared to siblings and general population peers. Addiction, 2008; 103: 1139–1148. | | | |
| --- | --- | --- | --- |
| **Study design**  **Treatment era**  **Years of follow-up** | **Participants** | **Variable definitions** | **Results** |
| Study design  Cross-sectional survey study  Treatment era  Diagnosis between 1970-1986.  Follow-up duration  Not reported | Type and number of participants  N = 10398 childhood cancer survivors  Cancer diagnosis  Not reported.  Age at diagnosis  Not reported.  Age at follow-up  Age at interview (only percentages given):   - 18–21 years: 24.1% - 22–26 years: 30.0% - 27–31 years: 23.3% - 32–48 years: 22.5%   Cancer treatment  Not reported.  Controls/normal population (if applicable)  N = 3034 siblings  Age at interview (only percentages given):   - 18–21 years: 17.6% - 22–26 years: 22.3% - 27–31 years: 23.3% - 32–48 years: 36.5%   N = 4774 population controls  Age at interview (only percentages given):  *Weighted percentages for national data reflecting survivors by gender, age and race/ethnicity:*   - 18–21 years: 24.1% - 22–26 years: 30.0% - 27–31 years: 23.3% - 32–48 years: 22.5%   *Unweighted data:*   - 18–21 years: 12.4% - 22–26 years: 14.3% - 27–31 years: 15.2% - 32–48 years: 58.0%   Additional participant characteristics (if applicable):  Not applicable | Alcohol measures  All alcohol measures were constructed from data using standard measures gathered on usual daily drinking (0–6+ drinks) and monthly frequency of alcohol consumption.  *Current (past year) alcohol consumption*  No other information provided.  *Risky drinking*   - For women: exceeding three drinks per day or seven drinks per week. - For men: exceeding four drinks per day or 14 drinks per week   *Heavy drinking*   - For women: five or more drinks per day at least once a month in the past year. - For men: six or more drinks per day at least once a month in the past year.   Other variables  Not applicable. | Factors associated with heavy drinking among survivors and peers  Models were adjusted for age, race, gender and education.  *Age at interview (32-48 years = ref.)*  Survivors:   - 18–21 years: OR = 2.7, 95% CI (1.9–3.9), p < .001 - 22–26 years: OR = 1.9, 95% CI (1.5–2.4), p < .001 - 27–31 years: OR = 1.3, 95% CI (1.0–1.7), p = NS   Controls:   - 18–21 years: OR = 2.0, 95% CI (1.5–2.6), p < .001 - 22–26 years: OR = 2.4, 95% CI (1.7–3.3), p < .001 - 27–31 years: OR = 1.5, 95% CI (1.0–2.1), p < .05   *Race/ethnicity (White and other = ref.)*  Survivors:   - Black, non-Hispanic: OR = 0.6, 95% CI (0.4–1.1), p = NS - Hispanic: OR = 1.0, 95% CI (0.7–1.4), p = NS   Controls:   - Black, non-Hispanic: OR = 0.7, 95% CI (0.4–1.2), p = NS - Hispanic: OR = 1.0, 95% CI (0.6–1.6), p = NS   *Gender (female = ref.)*  Survivors: OR = 2.1, 95% CI (1.8-2.6), p < .001  Controls: OR = 1.8, 95% CI (1.5-2.3), p < .011  *Education (college graduate = ref.)*  Survivors:   - Grades 0-12: OR = 3.4, 95% CI (2.7–4.4), p < .001 - Some post-high school: OR = 2.2, 95% CI (1.7–2.8), p <. 001   Controls:   - Grades 0-12: OR = 2.7, 95% CI (2.0–3.7), p < .001 - Some post-high school: OR = 1.8, 95% CI (1.3–2.6), p <. 01   *Age of first drink (years; 21+ = ref.)*  Survivors:   - <14: OR = 6.9, 95% CI (4.4–10.8), p < .001 - 15–16: OR = 5.3, 95% CI (3.5–8.1), p < .001 - 17–20: OR = 2.9, 95% CI (1.9–4.4), p < .001   Controls:   - <14: OR = 2.8, 95% CI (1.9–4.1), p < .001 - 15–16: OR = 1.5, 95% CI (1.0–2.2), p < .05 - 17–20: OR = 1.0, 95% CI (0.7–1.5), p = NS   Risk factors for heavy drinking  *Age at diagnosis (5-9 years = ref.)*  Survivors:   - 0–4: OR = 0.9, 95% CI (0.7–1.2), p = NS - 10–14: OR = 1.1, 95% CI (0.9–1.4), p = NS - 15–21: OR = 0.7, 95% CI (0.5–1.0), p = < .05   *General health (excellent/very good/good = ref.)*   - Fair/poor: OR = 1.5, 95% CI (1.1–1.9), p < .01   *Depression (normal = ref.)*   - Abnormal: OR = 1.7, 95% CI (1.4–2.2), p < .001   *Anxiety (normal = ref.)*   - Abnormal: OR = 1.4, 95% CI (1.1–1.9), p < .05   *Somatization (normal = ref.)*   - Abnormal: OR = 1.7, 95% CI (1.3–2.2), p < .001   *GSI score (normal = ref.)*   - Abnormal: OR = 1.9, 95% CI (1.5–2.4), p < .001   *Functional impairment (no impairment = ref.)*   - Impairment: OR = 1.0, 95% CI (0.7–1.3), p= NS   *Activity limitations (not limited at all = ref.)*   - Limited: OR = 1.3, 95% CI (1.1–1.5), p < .05   *Pain from cancer (none= ref.)*   - Some+: OR = 1.2, 95% CI (1.0–1.5), p = NS   *Anxiety about cancer (none= ref.)*   - Some+: OR = 1.2, 95% CI (1.0–1.4), p < .05   *IT methotrexate or cranial radiation (no = ref.)*   - Yes: OR = 0.7, 95% CI (0.5–0.8), p < .001   *Cancer diagnosis (leukaemia = ref.)*   - Central nervous system: OR = 0.9, 95% CI (0.7–1.3), p = NS - Hodgkin’s disease: OR = 1.4, 95% CI (1.0–1.8), p < .05 - Non-Hodgkin’s lymphoma: OR = 1.2, 95% CI (0.9–1.6), p = NS - Wilms tumour: OR = 1.5, 95% CI (1.1–2.1), p < .05 - Neuroblastoma: OR = 1.6, 95% CI (1.1–2.3), p < .05 - Sarcoma: OR = 1.3, 95% CI (0.9–1.7), p = NS - Bone: OR = 1.7, 95% CI (1.2–2.2), p < .001 |

Abbreviations: CCSS, childhood cancer survivors study, CI, confidence interval, GSI, global severity index, NS, non-significant, OR, odds ratio

| ***Fluehr et al.*** Factors Associated With Sun Protection Behaviours Among Childhood Cancer Survivors, J Pediatr Hematol Oncol, 2023, 45(3):e323-e327. | | | |
| --- | --- | --- | --- |
| **Study design**  **Treatment era**  **Years of follow-up** | **Participants** | **Variable definitions** | **Results** |
| Study design  Cross-sectional survey study  Treatment era  Not reported  Follow-up duration  *Time since diagnosis (years)*   - 0-16: N = 162 (20%) - 17-20: N = 216 (27%) - 21-24: N = 230 (29%) - 25+: N = 185 (23%) - Missing: N = 3 (3%) | Type and number of participants  N = 94 adult survivors of childhood cancer  Cancer diagnosis  Not reported.  Age at diagnosis  Not reported.  Age at follow-up  24.6 years (SD = 4.45; range 18-41)  Cancer treatment  Not reported.  Controls/normal population (if applicable)  Not applicable  Additional participant characteristics (if applicable):  Not applicable | Sun exposure measures  *Sun protective behaviours index score*  The Sun Habits survey consists of 7 protective behaviours when outside on a warm sunny day (wearing a shirt with sleeves, wearing long pants, wearing sunglasses, staying in the shade, using sunscreen, limiting time in the sun during midday, and wearing a hat). Participants reported their use of each behaviour on a 5-point ordinal scale ranging from 1 =never to 5 =always. The survey is scored as a composite average index score, which reflects overall engagement in sun protection behaviours.  Other variables  *Skin type*  A variation of the Fitzpatrick Skin Phototype Classification (FSPC) self-report measure was measured based on the responses to the question, “How would your skin react if, after several months of not being in the sun, you stayed outdoors in the midday summer sun for 1 hour without sunscreen?” Responses were measured based on a 6-point categorical scale ranging from “burn easily and do not get darker” to “not burn and not get darker” and dichotomized as “burn easily,” which was coded as 1 (e.g., “burn easily and not get darker/get a little darker”), or “not burn,” which was coded as 0 (e.g., “not burn and get darker/not get darker,” “burn minimally and get a lot darker,” and “burn moderately and get somewhat darker”).  *Barriers to sun protection*  E.g., cost, hassle, apply, inconvenience, comfort, and interference were measured on a 5-point ordinal scale ranging from 1= strongly disagree to 5 =strongly agree. The measure yields an overall average sun protection barriers score, with higher scores indicating more barriers.  *Absolute overall perceived severity*  Assessed using the question, “How severe do you think the health consequences of skin cancer are?” rated on a 5-point scale from 0= not at all severe to 5=very severe.  *Relative perceived severity*  Assessed using the question, “Compared with other types of cancer, how severe do you think the health consequences of skin cancer are?” rated on a 5-point scale from 0 =much less severe than other cancers to 5 =much more severe than other cancers.  *Overall perceived susceptibility*  Susceptibility of getting skin cancer assessed using the question, “How would you rate your chances of getting skin cancer in your lifetime?” using a 5-point response scale from 1=not at all likely to 5=very likely.  *Relative susceptibility*  Susceptibility of getting skin cancer relative to healthy peers (without a history of cancer) was assessed with the question “Compared with other people of your sex, age, and skin colour, who have not had childhood cancer, how would you rate your chances of getting skin cancer in your lifetime” using a 5-point scale from 1=much lower than people without a history of skin cancer to 5=much higher than other people without a history of skin cancer. | Associations between factors and sun exposure  Linear Regression Predicting Total Sun Protection Behaviours  The coefficient (*B*) indicates the change in Total Sun Protection Behaviours. E.g., history of radiation increased the total sun protection behaviours score by 0.27.  Age, sex, history of radiation therapy (Yes or No), and skin type were included in step 1; predictors from the Health Belief Model framework were included in step 2.  Step 1 model:   - Sex: *B* = 0.15, SE = 0.12, β = 0.12, p = 0.23 - Age (years):  *B* = 0.01, SE = 0.01, β =0.09, p = 0.40 - History of radiation (0 =no, 1 = yes):  *B* = 0.27, SE = 0.14, β =0.21, p = 0.06 - Skin type (0 = low risk, 1 = high risk):  *B* = 0.25, SE = 0.13, β = 0.20, p = 0.06   Step 2 model:   - Sex:  *B* = 0.05, SE = 0.12, β = 0.04, p = 0.66 - Age (years): *B* = 0.01, SE = 0.01, β = 0.10, p = 0.34 - History of radiation (0 = no, 1 = yes): *B* = 0.18, SE = 0.13, β =0.14, p = 0.19 - Skin type (0 = low risk, 1 = high risk): *B* = 0.31, SE = 0.13, β = 0.25, p = 0.02 - Barriers to sun behaviours:  *B* = - 0.16, SE = 0.09, β =−0.20, p = 0.09 - Overall susceptibility: *B* = - 0.05, SE = 0.06, β = −0.09, p = 0.46 - Relative susceptibility:  *B* = 0.04, SE = 0.06, β = 0.07, p = 0.52 - Overall risk: *B* = 0.02, SE = 0.07, β = 0.04, p = 0.73 - Relative risk: *B* = 0.18, SE = 0.07, β = 0.2, p = 0.02 |

Abbreviations: SD, standard deviation, SE, standard error

| ***Mayes et al.*** Health promotion and information provision during long-term follow-up for childhood cancer survivors: A service evaluation, J Pediatr. Hematol. Oncol, 2016, 33(6), 359–370. | | | |
| --- | --- | --- | --- |
| **Study design**  **Treatment era**  **Years of follow-up** | **Participants** | **Variable definitions** | **Results** |
| Study design  Single semi structured interviews  Treatment era  Not reported  Follow-up duration  Median time since treatment 10 years, range 5-19 | Type and number of participants  N=51 childhood cancer survivors  Cancer diagnosis   - Leukaemia: N=12 (23.5%) - Hodgkin lymphoma: N=10 (19.6%) - Central nervous system tumour: N=6 (11.8%) - Extracranial tumour: N=23 (45.1%)   Age at diagnosis  Median 11.3 years, range 2-22.  Age at follow-up  Median 21.8 years, range 14-33  Cancer treatment  Not reported  Controls/normal population (if applicable)  Not applicable  Additional participant characteristics (if applicable):  Not applicable | Survivors’ views on the provision of health promotion information. | Common sources of lifestyle information and advice were reported to be the tnternet (49%), school (12%), magazines and TV (12%), friends and family (10%), or spoken/written information from hospital staff (10%).  The majority of survivors (n = 46, 90%) said that they had discussed lifestyle advice at some stage during treatment or LTFU. Approximately half of survivors (n = 28, 55%) believed that the initiative to begin a health promotion conversation during LTFU should lie with the consultant paediatric/adolescent oncologist. The remainder believed that consultants should be prepared to discuss these issues if the survivor wished but that these conversations should not be mandatory. Most survivors (n = 45, 88%) felt that a separate appointment for lifestyle advice and discussion would not be necessary.  Most survivors (n = 45, 88%) commented that if lifestyle advice were to be discussed, a hospital-based health care professional such as their consultant paediatric/adolescent oncologist or specialist nurse would be the best person to provide advice, whilst the remainder felt that their general practitioner (GP) would be the best person to ask. There was no agreement concerning when this information should be given to patients. Forty-five (88%) survivors agreed that it is important to receive further information and that they would like to know the full facts and consequences of their treatment, but also that they did not want to be continually reminded when more immediately important topics might be on their mind. |

| **Keats MR et al.** Understanding physical activity in adolescent cancer survivors: an application of the theory of planned behaviour. Psychooncology, 2007;16:448-57. | | | |
| --- | --- | --- | --- |
| **Study design**  **Treatment era**  **Years of follow-up** | **Participants** | **Variable definitions** | **Results** |
| Study design  Self-administered, mailed questionnaire in a retrospective design.  Treatment era  Not reported.  Follow-up duration  Mean follow-up after diagnosis was 31.7 months (SD 24.0). | Type and number of participants  N=59 adolescent survivors of cancer  Cancer diagnosis   - Lymphoma: N=20 (33.9%) - Leukaemia: N=15 (25.4%) - CNS tumours: N=8 (13.6%) - Osteosarcoma: N=5 (8.5%) - Ovarian/testicular: N=4 (6.8%) - Other: N=7 (11.9%)   Age at diagnosis  Between ages of 11 and 19 years. No other information reported.  Age at follow-up  Between ages of 11 and 19 years at time of diagnosis   - 15-16 years: N=14 (23.7%) - 17-18 years: N=32 (54.2%) - 19-20 years: N=13 (22.0%)   Cancer treatment  *Adjuvant therapy*   - Radiation alone: N=4 (6.8%) - Chemotherapy: N=26 (44.1%) - Radiation and chemotherapy: N=15 (25.4%) - Radiation and BMT: N=1 (1.7%) - Chemotherapy and BMT: N=1(1.7%) - Radiation chemotherapy and BMT: N=2 (3.4%) - None: N=9 (15.3%) - Missing: N=1 (1.7%)   *Surgery*   - Biopsy/tumour removal: N=25 (78.0%) - Amputation/MSK reconstructive: N=5 (15.6%) - Other: N=2 (6.3%)   Controls/normal population (if applicable)  Not applicable  Additional participant characteristics (if applicable):  BMI:   - Underweight: N=8 (13.8%) - Normal: N=36 (62.1%) - Overweight: N=11 (19.0%) - Obese: N=3 (5.2%)   N=7 participants (11.9%) were still receiving some form of maintenance therapy. | Physical activity outcomes  *Total physical activity*  Defined as frequency of physical activity reported per week within each intensity category multiplied by average reported duration, weighted by an estimate of the MET summed across all intensities, and expressed as average MET hours per week.  Other variables  Behavioural beliefs for a physically active lifestyle  Collected by asking participants to rate their belief strength and outcome evaluation. Each belief was preceded by the statement: “Since your cancer diagnosis, being physically active on a regular basis would help you …” and was rated on a 7-point scale ranging from 1 (extremely unlikely) to 7 (extremely likely).  *Normative beliefs.*  Each normative belief was preceded with the statement: “Since your cancer diagnosis, how strongly would each of the following persons approve or disapprove of your Physical Activity in Adolescent Cancer Survivors being physically active?” and was rated a 7-point scale ranging from 1 (extremely unimportant) to 7 (extremely important).  *Control beliefs*  Each control belief was preceded by the statement “Since your cancer diagnosis, how true are the following …” and was rated on a 7-point scale ranging from 1 (extremely false) to 7 (extremely true). Perceived power was assessed by the statement “Since your cancer diagnosis, how strongly does each of the following influence your physical activity levels?” and was rated on a 7-point scale ranging from 1 (extremely weak) to 7 (extremely strong). | Total physical activity  Physical activity post-treatment was regressed on intention (Step #1) followed by self-efficacy and perceived control (Step #2). β represents the increase in the odds of post-diagnosis physical activity.  Hierarchical regression analyses:  Independent correlates of post-diagnosis physical activity:   - Intention: β = 0.31, p = 0.023 - Self-efficacy: β = 0.42, p = 0.04 - Perceived control: β = -0.12, p = NS   Independent correlates of intention of physical activity:   - Affective attitude: β =0.32, p = .016 - Instrumental attitude: β =0.31, p = .037 - Self-efficacy: β = 0.20, p = NS - Perceived control: β = -0.09, p = NS - Subjective norm: β = 0.02, p = NS   Behavioural beliefs for a physically active lifestyle  The most salient beliefs for adolescent cancer survivors were:   - Keep physically fit, stay strong, and look good (N=23) - Stay healthy (N=12) - Stay busy and stay connected with friends (N=13) - Feel normal (N=11) - Weight management (N=10) - Increase energy (N=8) - Have fun, increase self-confidence and feel a sense of accomplishment (N=7) - Reduce stress, Relieve frustrations and relax (N=3) - Recover from treatment (N=1)   Control beliefs with respect to participation in physical activity:   - Felt lazy or unmotivated to be physically active (N=15) - Being too busy or not having enough time (N=12) - Experienced physical limitations, e.g. being unfit, lack of stamina or strength, poor balance, fear of injury, treatment-related side effects (N=11) - Experienced fatigue or soreness (N=6) - Lack of ability, skill or confidence (N=6) - Lack of money or access to resources, e.g. fitness facilities (N=6) - Negative parental influence, i.e. over protective or not encouraging physical activity (n=1) |

Abbreviations: MET, metabolic equivalent, NS, non-significant, SD, standard deviation

| **Arroyave et al.** Childhood cancer survivors' perceived barriers to improving exercise and dietary behaviours. Oncol Nurs Forum. 2008; 35:121-30. | | | |
| --- | --- | --- | --- |
| **Study design**  **Treatment era**  **Years of follow-up** | **Participants** | **Variable definitions** | **Results** |
| Study design  Cross-sectional survey study  Treatment era  Not reported  Follow-up duration  Not reported | Type and number of participants  N=118 childhood cancer survivors  Cancer diagnosis   - Central nervous system tumour: N=47 (39.8%) - Leukaemia: N=49 (41.5%) - Lymphoma: N=22 (18.6%)   Age at diagnosis  Mean age 8.8 years (SD 4.8 years)  Age at follow-up  Mean age 21.6 years (SD 5.8 years)  Cancer treatment  Not reported  Controls/normal population (if applicable)  Not applicable  Additional participant characteristics (if applicable):  Not applicable | Barriers to exercise  No other information reported.  Barriers to a healthful diet  A healthful diet is low in fat and rich in fruits and vegetables, whole grains, and calcium. | Barriers to physical exercise:  (% indicates agree of strongly agree)   - Being too tired (57%) - Being too busy (53%) - Do not belong to a gym (48%) - Rather prefer to watch television or read (44%) - No one to exercise with (31%) - Bad weather (30%) - Feel self-conscious (29%) - Do not enjoy it (28%) - Friends do not exercise (27%) - No willpower (25%) - No access to equipment (22%) - Worry about injury (20%) - Do not like to sweat (20%) - No support (19%) - Do not want to be sore (19%) - Unsure how to exercise (17%) - No place to exercise (9%)   Barriers to eating a healthy diet  *Barriers to eating more fruits and vegetables*   - Hard to get when dining out (30%) - Do not like the taste (19%) - Not available at home (12%) - Cost too much (11%) - Take too long to prepare (10%) - Hurt stomach (8%) - Friends do not eat them (5%) - Family does not like them (3%)   *Barriers to eating more whole grains*   - Hard to get when dining out (31%) - Do not like the taste (22%) - Family does not like them (15%) - Not available at home (14%) - Friends do not eat them (12%) - Cost too much (9%) - Take too long to prepare (5%) - Hurt stomach (4%)   *Barriers to eating more high-calcium foods*   - Hard to get when dining out (15%) - Hurt stomach (14%) - Not available at home (7%) - Do not like the taste (6%) - Friends do not eat them (5%) - Family does not like them (2%)   *Barriers to limiting high-fat foods*   - Commercials make high-fat foods tempting (58%) - Hard to get low-fat foods when dining out (50%) - Friends eat a lot of high-fat foods (50%) - Family eats a lot of high-fat foods (43%) - Low-fat foods do not taste good (42%) - No willpower (41%) - Low-fat foods do not fill me up (29%) - Do not know how to choose lower-fat foods (23%) - Low-fat foods not available at home (11%) |

Abbreviations: SD, standard deviation

| ***Marchak et al.*** Cancer-Related Barriers to Health Behaviours Among Adolescent and Young Adult Survivors of Pediatric Cancer and Their Families. J. Adolesc. Young Adult Oncol, 2023, 12(1), 118-122. | | | |
| --- | --- | --- | --- |
| **Study design**  **Treatment era**  **Years of follow-up** | **Participants** | **Variable definitions** | **Results** |
| Study design  Web-based semi-quantitative survey study  Treatment era  Not reported  Follow-up duration  Time off therapy: mean 13.1 years (SD 5.4, range 4-25) | Type and number of participants  N=27 childhood cancer survivors  Cancer diagnosis  Not reported  Age at diagnosis  Not reported  Age at follow-up  Mean age 24.0 years (SD 3.33)  Cancer treatment  Not reported  Controls/normal population (if applicable)  Not applicable  Additional participant characteristics (if applicable):  Not applicable | Cancer-related barriers to physical activity and good nutrition. | Cancer-related barriers to physical activity   - Fatigue: N=17 (63%) - Decreased strength: N=14 (51.9%) - Low motivation for exercise: N=8 (29.6%) - Exercise limitations due to physical changes: N=12 (44.4%) - Too much screen time: N=4 (14.8%) - Fears related to injury: N=9 (33.3%) - Weight gaining leading to trouble with being physically active: N=5 (18.5%) - Doctors continuing to limit physical activities: N=4(14.8%) - Other: N=2 (7.4%)   Cancer-related barriers to good nutrition:   - Picky eating: N=12 (44.4%) - Increased unhealthy foods or snacks: N=9 (33.3%) - Eating too little or getting full easily: N=5 (18.5%) - Relying on fast food or take out instead of cooking at home: N=5 (18.5%) - Limited willingness to eat fruits or vegetables: N=5 (18.5%) - Using unhealthy food as a reward: N=3 (11.1%) - Eating too much or hungry all the time: N=4 (14.8%) - Drinking sugary beverages: N=4 (14.8%) - Other: N=4 (14.8%) |

Abbreviations: SD, standard deviation

| ***Alexander et al***. Improving Food Literacy and Access Among Young Adult Cancer Survivors: A Cross-Sectional Descriptive Study. Cancer Nursing, 2022, 45: 161-166. | | | |
| --- | --- | --- | --- |
| **Study design**  **Treatment era**  **Years of follow-up** | **Participants** | **Variable definitions** | **Results** |
| Study design  Cross-sectional descriptive survey study  Treatment era  Not reported  Follow-up duration  Time since treatment completion:   - 11 months or less: N=2 (1.6%) - 1-5 years: N=20 (16.1%) - 6-10 years: N=19 (15.3%) - 11-15 years: N=31 (25.0%) - 16-20 years: N=32 (25.8%) - 21-25 years: N=12 (9.7%) - 26 years or greater: N=8 (6.5%) | Type and number of participants  N=124 young adult cancer survivors  Cancer diagnosis  Not reported  Age at diagnosis   - Less than 12 months: N=10 (8.1%) - 1-4 years: N=34 (27.4%) - 5-9 years: N=24 (19.4%) - 10-14 years: N=38 (30.6%) - 15-17 years: N=18 (14.5%)   Age at follow-up   - 18-21 years: N=44 (35.5%) - 22-26 years: N=42 (33.9%) - 27-30 years: N=22 (17.7%) - 31-35: N=8 (6.5%) - 36-39: N=8 (6.5%)   Cancer treatment  Not reported  Controls/normal population (if applicable)  Not applicable  Additional participant characteristics (if applicable):  Not applicable | *Perceived importance of nutrition and desire to improve diet*  Measured with individual Likert items, and nutrition education preferences were measured through two 5-item Likert scales, one inquiring about desired format (e.g., computer, cell phone, face-to-face, hard copy) and another focused on social support (e.g., self, other survivors, nutritionist, group meeting). | Perceived importance of nutrition and desire to improve diet.   - 32.3% are interested in group meetings - 63.7% seek the support of a nutritionist to help them acquire healthy nutrition habits - 41.9% seeks the support of another survivor to help him or her acquire healthy nutrition habits - 89.5% express the desire for self-help to improve dietary intake - 61.3% prefer the use of digital/print materials alone |

| ***Bouwman et al.*** Perceived barriers and facilitators to health behaviours in European childhood cancer survivors: A qualitative PanCareFollowUp study. Cancer Med, 2023, 12: 12749-12764. | | | |
| --- | --- | --- | --- |
| **Study design**  **Treatment era**  **Years of follow-up** | **Participants** | **Variable definitions** | **Results** |
| Study design  Focus groups and semi-structured telephone interviews  Treatment era  Before 2001.  Follow-up duration  Years since completion of treatment (years):   - Focus group participants (N=12): Median 14 years (range 3–28) - Semi-structured interviews (N=20): Median 20 years (range 9–40) | Type and number of participants  N=32 childhood cancer survivors, N=12 for focus groups and N=20 for interviews.  Cancer diagnosis  Only reported per focus group/interview group (n/n):   - Leukaemia: 5/7 - Central nervous system tumours and miscellaneous intracranial and intraspinal neoplasms: 1/3 - Lymphomas and reticuloendothelial neoplasms: 0/2. - Germ cell, trophoblastic, and other gonadal neoplasms: 2/0 - Soft tissue sarcomas:0 /2 - Malignant bone tumours: 3/3 - Renal tumours: 1/2 - Other and unspecified: 0/1   Age at diagnosis  Not reported  Age at follow-up   - Focus group participants (N=12): median age 29 years (range 21– 39) - Interview participants (N=20): median 30.5 years (range 22– 46)   Cancer treatment  Not reported  Controls/normal population (if applicable)  Not applicable  Additional participant characteristics (if applicable):  Not applicable | *Healthy behaviours*  Defined as engaging in regular physical activities, maintaining a healthy diet, limiting alcohol consumption, and avoiding tobacco and drug use. | Barriers and facilitators regarding healthy behaviours (by domain)  *Healthy lifestyle knowledge*  Barriers:   - Healthcare professionals providing insufficient knowledge on importance of health behaviours in childhood cancer survivors   Facilitators:   - Knowledge of importance health behaviours for childhood cancer survivor population - Healthcare professionals providing knowledge on importance of health behaviours for childhood cancer survivors - Healthcare professionals providing knowledge on how to engage in healthy behaviours - Knowledge of family/friends/yourself on healthy behaviours   *Consequences*  Facilitators:   - Physical health benefits as consequences of healthy behaviours - Long-term health benefits as consequences of healthy behaviours   Environmental context and resources  Barrier:   - Lack of available time for healthy behaviours   Facilitators:   - Available professional support to stimulate healthy behaviours - Work environment stimulating healthy behaviours - Social environment positively influencing healthy behaviours   *Social influences*  Barriers:   - Unhealthy behaviours by people in close environment - Lack of social support in adopting healthy behaviours - (Social) Media stimulating unhealthy behaviours   Facilitators:   - Healthy behaviours of people in close environment - Social support by people in close environment stimulating healthy behaviours - (Social) Media stimulating healthy behaviours - Dealing with negative influences from people in social environment   *Beliefs about capabilities*  Facilitators:   - Physical health benefits as consequences of healthy behaviours - Long-term health benefits as consequences of healthy behaviours   *Reinforcement*  Barrier:   - Lower motivation to engage in healthy behaviours by personal related aspects   Facilitators:   - Positive reinforcement by personal-related incentives - Positive reinforcement by social/societal incentives - Positive reinforcement by sport activity-related incentives - Positive reinforcement by distal rewarding of health behaviours - Positive reinforcement by proximal rewarding of health behaviours   *Memory, attention and decision processes*  Facilitators:   - Healthy behaviours due to conscious decision-making - Healthy behaviours embedded in memory   *Skills*  Facilitator:   - Learning how to deal with physical limitations when wanting to engage in physical activity   *Emotion*  Barrier:   - Stress negatively affecting healthy behaviours   *Behavioural regulation*  Facilitator:   - Good planning to maintain healthy behaviours |

| ***Dugan et al.*** Exploring Social Ecological Determinants of Physical Activity Among Adult Survivors of Childhood Cancer. J Adolesc Young Adult Oncol. 2021, 10(3):316-325. | | | |
| --- | --- | --- | --- |
| **Study design**  **Treatment era**  **Years of follow-up** | **Participants** | **Variable definitions** | **Results** |
| Study design  A qualitative concept elicitation survey  Treatment era  Not reported  Follow-up duration  Mean time since therapy completion: 14.35 years (SD 9.18, range 1-33 years)   - <5 years: N=3 (18%) - 5–10 years: N=5 (29%) - 11–20 years: N=5 (29%) - >20 years: N=4 (24%) | Type and number of participants  N=17 adult survivors of childhood cancer  Cancer diagnosis   - Leukaemia: N=9 (53%) - Lymphoma: N=3 (18%) - Brain/CNS: N=3 (18%) - Sarcoma: N=2 (12%) - Metastasis: N=3 (17%) - Relapse: N=5 (28%)   Age at diagnosis  Mean age: 8.49 years (SD 4.94, range 10 months - 17 years)  Age at follow-up  Mean age: 26.59 years (SD 6.60, range 19-39 years)  Cancer treatment  Not reported  Controls/normal population (if applicable)  Not applicable  Additional participant characteristics (if applicable):  Not applicable | Barriers, facilitators, and resources of physical activity. | Barriers and facilitators of physical activity by different levels:  *Individual barriers (N=24)*   - Health related (N = 9) - Time/schedule (N = 3) - Motivation (N = 3) - Life changes (N = 1)   *Microsystem barriers (N=11)*   - School/work (N = 4) - Proximity/access (N = 1) - Moving (N = 2)   *Macrosystem barriers (N=4)*   - Finances (N=1)   *Individual facilitators (N=3)*   - Time/schedule (N = 2) - Motivation (N = 1)   *Microsystem facilitators (N=19)*   - People (N = 6) - Proximity/access (N = 3) - Equipment (N = 1)   *Exosystem facilitators (N=1)*   - Places and policy (e.g. obligatory dance classes; N = 1) |

Abbreviations: SD, standard deviation

| **Le et al.** A home-based physical activity intervention using activity trackers in survivors of childhood cancer: A pilot study. Pediatr Blood Cancer, 2017, 64.2: 387-394. | | | |
| --- | --- | --- | --- |
| **Study design**  **Treatment era**  **Years of follow-up** | **Participants** | **Variable definitions** | **Results** |
| Study design  Single centre pre-post intervention study with pre-post surveys on barriers to exercise  Treatment era  Not reported  Follow-up duration  Mean years since diagnosis:  14.4 (SD 8.0, range 1.7−28.9) | Type and number of participants  N=19 childhood cancer survivors  Cancer diagnosis   - Leukaemia: N=9 (47.3%) - Lymphoma: N= 3 (15.8%) - Sarcoma: N= 5 (26.3%) - CNS tumour: N= 1 (5.3%) - Wilms tumour: N= 1 (5.3%)   Age at diagnosis  Not reported  Age at follow-up  Mean age: 24.3 years (SD 5.8, range 15-35 years)  Cancer treatment  Not reported  Controls/normal population (if applicable)  Not applicable  Additional participant characteristics (if applicable):  Not applicable | Self-reported physical activity barriers, preferences, and beliefs pre- and post-intervention. | *Barriers to exercise*   - Lack of time - Lack of support or companionship from family and friends - Lack of energy - Lack of motivation - Lack of knowledge - Lack of access to exercise resources or facilities - Fear of injury   *Beliefs*   - “I believe regular exercise can help me deal with some of the long-term side effects of my therapy” - “I believe that participating in more exercise can help me maintain my health” |

Abbreviations: SD, standard deviation
