# Supplementary material for: Barriers, facilitators, and other factors associated with health behaviors in childhood, adolescent, and young adult cancer survivors: A systematic review
Source: Cancer Med. 2024 Jun 21;13(12):e7361. doi: 10.1002/cam4.7361 (PMC11192647; doi:10.1002/cam4.7361)
Supplement: Supplementary file 2 — Appendix S2. [file CAM4-13-e7361-s001.docx]

Supplementary File B. Overview of non-significant and corresponding significant results

|  | **N** **studies** **significant** | **N** **studies** **non-significant** |
| --- | --- | --- |
| **General** **behavioural** **health** | | |
| *Decreased* *poor* *overall* *behavioural* *health* *(Klosky* *et* *al.)* | | |
| Gender | 0 | 1 |
| Race | 0 | 1 |
| Age | 0 | 1 |
| Age at diagnosis | 0 | 1 |
| Cancer diagnosis | 0 | 1 |
| CNS treatment (no vs. any) | 0 | 1 |
| Household income | 0 | 1 |
| **Sun** **exposure** | | |
| *Low* *adherence* *to* *sunbathing* *recommendations* *(Zwemer* *et* *al.)* | | |
| Age at diagnosis | 0 | 1 |
| Years from diagnosis | 0 | 1 |
| Education | 0 | 1 |
| Household income | 0 | 1 |
| Current employment | 0 | 1 |
| Current school going | 0 | 1 |
| Race/ethnicity | 0 | 1 |
| Cancer diagnosis | 0 | 1 |
| History of radiation | 0 | 1 |
| Perceived skin cancer risk | 0 | 1 |
| Tanning booth use in the last year | 0 | 1 |
| Perceived vulnerability to appearance changes | 0 | 1 |
| Perceived vulnerability to skin cancer | 0 | 1 |
| *Low* *adherence* *to* *recommendations* *during* *incidental* *sun* *exposure* *(Zwemer* *et* *al.)* | | |
| Current age | 0 | 1 |
| Age at diagnosis | 0 | 1 |
| Years from diagnosis | 0 | 1 |
| Gender | 0 | 1 |
| Education | 0 | 1 |
| Household income | 0 | 1 |
| Current employment | 0 | 1 |

| Current school going | 0 | 1 |
| --- | --- | --- |
| Race/ethnicity | 0 | 1 |
| Cancer diagnosis | 0 | 1 |
| History of radiation | 0 | 1 |
| Perceived skin cancer risk | 0 | 1 |
| Tanning booth use in the last year | 0 | 1 |
| Perceived vulnerability to skin cancer | 0 | 1 |
| *Sunscreen* *use* *(Darabos* *et* *al.;* *Cheung* *et* *al.)* | | |
| Private insurance | 0 | 1 |
| Age at diagnosis | 0 | 2 |
| Age | 0 | 1 |
| Time since diagnosis | 0 | 1 |
| Cancer diagnosis | 0 | 1 |
| Education | 1 (Cheung et al.) | 1 (Darabos et al.) |
| Time off treatment | 0 | 1 |
| Brain tumor | 0 | 1 |
| Cancer relapse | 0 | 1 |
| Baseline age | 0 | 1 |
| Treatment intensity | 0 | 1 |
| Solid tumor | 0 | 1 |
| *Sunprotective* *behaviours* *(Fluehr* *et* *al.)* | | |
| Perceived barriers to sun protection | 0 | 1 |
| Sex | 0 | 1 |
| Age | 0 | 1 |
| History of radiation | 0 | 1 |
| Overall susceptibility | 0 | 1 |
| Relative susceptibility | 0 | 1 |
| Overall risk | 0 | 1 |
| **Diet** | | |
| *Diet* *quality* *(adjusted* *means* *of* *HEI-2010* *score;* *Zhang* *et* *al.)* | | |
| Race/ethnicity | 0 | 1 |
| Alcohol consumption | 0 | 1 |
| Height | 0 | 1 |
| Any radiation | 0 | 1 |
| Brain radiation | 0 | 1 |

| Cumulative alkylating agent dose | 0 | 1 |
| --- | --- | --- |
| Cumulative anthracycline dose | 0 | 1 |
| *Vitamin* *D* *deficiency* *(Bhandari* *et* *al.)* | | |
| Other race than black/Hispanic (vs. non-Hispanic white) | 0 | 1 |
| *Not* *meeting* *fruit/vegetable* *intake* *recommendations* *(Darabos* *et* *al.)* | | |
| Ethnicity | 0 | 1 |
| Sex | 0 | 1 |
| Race | 0 | 1 |
| Private insurance | 0 | 1 |
| Age | 0 | 1 |
| Age at diagnosis | 0 | 1 |
| Treatment intensity | 0 | 1 |
| Time since diagnosis | 0 | 1 |
| Time off treatment | 0 | 1 |
| Solid tumor | 0 | 1 |
| Brain tumor | 0 | 1 |
| *Balanced* *diet* *(Cheung* *et* *al.)* | | |
| Age at diagnosis | 0 | 1 |
| Sex | 0 | 1 |
| Education | 0 | 1 |
| Private medical insurance | 0 | 1 |
| Monthly household income | 0 | 1 |
| **Smoking** | | |
| *Higher* *smoking* *rates* *(Emmons* *et* *al.)* | | |
| High school grad (vs. more than high school) | 0 | 1 |
| *Quit* *attempts* *(Emmons* *et* *al.)* | | |
| High school grad (vs. more than high school) | 0 | 1 |
| *Smoking* *cessation* *(Emmons* *et* *al.;* *Bougas* *et* *al.)* | | |
| Age | 0 | 2 |
| Sex | 0 | 1 |
| Positive feelings | 0 | 1 |
| Depressive symptoms | 0 | 1 |
| Age at diagnosis | 0 | 1 |
| Cancer type | 0 | 1 |

| Employment status | 0 | 1 |
| --- | --- | --- |
| Chemotherapy | 0 | 1 |
| Radiation on thorax | 0 | 1 |
| Cardiovascular disease | 0 | 1 |
| Quality of life scores | 0 | 1 |
| *Recent* *smoking* *(Kahalley* *et* *al.)* | | |
| Age | 0 | 1 |
| Sex | 0 | 1 |
| *Current* *smoking* *(Bougas* *et* *al.;* *Capelli* *et* *al.;* *Darabos* *et* *al.;* *Cheung* *et* *al.)* | | |
| Age at diagnosis | 0 | 3 |
| Cancer types (except CNS) | 0 | 2 |
| Employment status | 0 | 1 |
| Sex | 2 (Bougas et al., Darabos et al.) | 2 (Capelli et al.; Cheung et al.) |
| Marijuana use | 0 | 1 |
| Ethnicity | 0 | 1 |
| Private insurance | 0 | 2 |
| Age | 1 (Bougas et al.) | 2 (Cheung et al; Darabos et al.) |
| Treatment intensity | 0 | 1 |
| Time since diagnosis | 0 | 1 |
| Time off treatment | 0 | 1 |
| Solid tumor | 0 | 1 |
| Brain tumor | 0 | 1 |
| Cancer relapse | 0 | 1 |
| Monthly household income | 0 | 1 |
| **Alcohol** **use** | | |
| *Binge* *drinking* *(Cappelli* *et* *al.;* *Darabos* *et* *al.)* | | |
| Cigarette use | 0 | 1 |
| Spirituality | 0 | 1 |
| Perceived stress scale | 0 | 1 |
| Ethnicity | 0 | 1 |
| Sex | 1(Cappelli et al.) | 1 (Darabos et al.) |
| Race | 0 | 1 |
| Private insurance | 0 | 1 |
| Age at diagnosis | 0 | 1 |
| Time since diagnosis | 0 | 1 |

| Time off treatment | 0 | 1 |
| --- | --- | --- |
| Brain tumor | 0 | 1 |
| Cancer relapse | 0 | 1 |
| *Alcohol* *consumption* *(Cheung* *et* *al.)* | | |
| Age | 0 | 1 |
| Age at diagnosis | 0 | 1 |
| Cancer diagnosis | 0 | 1 |
| Private medical insurance | 0 | 1 |
| Monthly household income | 0 | 1 |
| *Heavy* *drinking* *(Lown* *et* *al.)* | | |
| Race | 0 | 1 |
| Functional impairment | 0 | 1 |
| Pain from cancer | 0 | 1 |
| **Physical** **activity** | | |
| *Physical* *activity* *(Darabos* *et* *al.)* | | |
| Age | 0 | 1 |
| Age at diagnosis | 0 | 1 |
| Sex | 0 | 1 |
| Cancer diagnosis | 0 | 1 |
| Education | 0 | 1 |
| Private medical insurance | 0 | 1 |
| Monthly household income | 0 | 1 |
| *Not* *meeting* *physical* *activity* *recommendations* *(Florin* *et* *al.;* *Ness* *et* *al.)* | | |
| Current smoking | 0 | 2 |
| Chemo only in males (vs. Chemo + RT) | 0 | 1 |
| College graduate vs. < high school | *0* | 1 |
| Unemployed/looking for work vs. working/caring home for family | 0 | 1 |
| Depression | 0 | 1 |
| Chemotherapy without anthracyclines (vs. no) in females | 0 | 1 |
| Chest radiation without cranial radiation (vs. no) in females | 0 | 1 |
| Chemotherapy with and without anthracyclines (vs. no) in males | 0 | 1 |
| Other radiation vs. no radiation in males | 0 | 1 |
| *Leisure* *time* *physical* *activity* *(Florin* *et* *al.)* | | |
| Chemo only (vs. chemo + CRT) | 0 | 1 |
| *Inactive* *lifestyle* *(Ness* *et* *al.)* / *inactivity* *(Rueegg* *et* *al.)* | | |

| Hispanic & Other ethnicity | 0 | 1 |
| --- | --- | --- |
| Being a student (vs. working/caring home for family) | 0 | 1 |
| Overweight (vs. normal weight) | 0 | 1 |
| Ever smoking (vs. never) | 0 | 1 |
| Other radiation & chest radiation without cranial radiation in females and  males (vs. no) | 0 | 1 |
| Chemotherapy without anthracyclines in males (vs. no chemotherapy) | 0 | 1 |
| Age | 0 | 1 |
| Migration background | 0 | 1 |
| Civil status | 0 | 1 |
| Having children | 0 | 1 |
| Smoking | 0 | 1 |
| Treatment | 0 | 1 |
| Age at diagnosis | 0 | 1 |
| Cancer diagnosis | 0 | 1 |
| Self-reported late effects | 0 | 1 |
| *Engaging* *in* *active* *transportation* *(Slater* *et* *al.)* | | |
| Age | 0 | 1 |
| BMI | 0 | 1 |
| Vehicles per driver | 0 | 1 |
| Environmental barriers | 0 | 1 |
| Health barriers | 0 | 1 |
| Objective walkability | 0 | 1 |
| *No* *sports* *(Rueegg* *et* *al.)* | | |
| Age | 0 | 1 |
| Sex | 0 | 1 |
| Migration background | 0 | 1 |
| Civil status | 0 | 1 |
| Treatment | 0 | 1 |
| Age at diagnosis | 0 | 1 |
| Cancer diagnosis | 0 | 1 |
| Self-reported late effects | 0 | 1 |

NB: not all significant results in Table 4 have corresponding NS results reported in this table (e.g., physical activity – sex: this is found to be a significant factor for 3 outcomes in 3 different studies, but for those 3 outcomes there were no NS results and thus no separate row in this table).
